# Effectiveness of interventions for preventing road traffic injuries: A systematic review in low-, middle- and high-income countries

**DOI:** 10.1371/journal.pone.0312428

**Published:** 2024-12-05

**Authors:** Maryam Akbari, Seyed Taghi Heydari, Alireza Razzaghi, Mohebat Vali, Reza Tabrizi, Kamran Bagheri Lankarani

**Affiliations:** 1 Institute of Health, Health Policy Research Center, Shiraz University of Medical Sciences, Shiraz, Iran; 2 Research Institute for Prevention of Non-Communicable Diseases, Children Growth Research Center, Qazvin University of Medical Sciences, Qazvin, Iran; 3 Department of Epidemiology, Assistant Professor of Epidemiology, School of Health, Shiraz University of Medical Sciences, Shiraz, Iran; 4 Noncommunicable Diseases Research Center, Fasa University of Medical Science, Fasa, Iran; Jazan University College of Applied Medical Science, SAUDI ARABIA

## Abstract

**Background:**

Road traffic collisions (RTCs) are the primary cause of death, which usually occur during the most crucial years of life, resulting in significant damage to health, society, and the economy. A wide variety of strategies and policies have been implemented around the world to minimize injuries and fatalities resulting from RTCs. This study aimed to systematically evaluate the effectiveness of interventions to reduce road traffic injuries (RTIs) in low-, middle-, and high-income countries.

**Methods:**

The researchers looked for articles in many databases (PubMed, Web of Science, Google Scholar, Scopus, Embase, PsycInfo, OpenGrey, EconLit, IMEMR, AIM, Cochrane Injuries Group’s specialized register, Transport Research International Documentation (TRID), Transportation Research Information Services (TRIS) Database and the OECD’s Joint Transport Research Centre’s International Transport Research Documentation (ITRD)) about ways to reduce RTIs and included articles published up to December 2023. The study area did not matter; only RTI reduction methods were considered. Two people checked the articles to ensure being relevant and qualified and summarized what they found in the articles.

**Results:**

A total of 852 articles were included in this systematic review. Most interventions were related to legislation (26.4%) and enforcement (17.0%), and the minor interventions were related to social marketing (4.9%) and traffic user safety (2.2%). Regarding income level (based on the latest classification of the World Bank—2020), more than half of the interventions (83.7%) took place in developed and high-income countries. Regarding intervention types, legislative and road safety interventions effectively reduced road traffic crashes by 26% and 16.7%, respectively. The results indicated that interventions in high-income countries were more likely to minimize RTCs than other countries. However, this difference was not statistically significant (p-value = 0.982). Most effective interventions (36.1%) were reported during the Decade of Action for Road Safety (2011–2020).

**Conclusion:**

Current road safety efforts prioritize changing how people behave on the roads (training, laws, enforcement) over making the roads safer. Focusing on fixing the entire system rather than blaming drivers ("system repair") is necessary for a complete picture.

## Introduction

According to the World Health Organization (WHO), a road traffic accident happens when a moving vehicle crashes on a public or private road and injures or kills at least one person [1]. Crashes on the road can be terrible news, causing financial burdens, injuries, or even death. WHO defines a *road traffic accident* as a public road crash involving at least one moving vehicle resulting in injury or death [2].

Road Traffic Crashes (RTCs) have surpassed HIV/AIDS, tuberculosis, and diarrhea to become the eighth most common cause of death across all age groups [3]. According to the WHO Global Status Report on Road Safety (GSRRS) in 2023, RTCs claim the lives of approximately 1.19 million individuals annually while leaving 20 to 50 million others with non-fatal injuries. RTCs are the primary cause of death among children and young adults aged 5 to 29 worldwide. More than 85% of all injury-related deaths worldwide are attributed to low- and middle-income countries (LMICs) that lack adequate healthcare resources [4]. The WHO highlights that road traffic injuries (RTIs) cause immense human suffering and substantial economic burdens for individuals, their families, and governments. These financial losses encompass the expenses associated with healthcare services, such as rehabilitation and accident investigations, as well as the reduced or lost productivity of individuals who have either died or become disabled due to injuries. Moreover, family members are often compelled to balance work or education with caregiving responsibilities for the injured. Addressing road traffic fatalities and injuries is crucial worldwide due to representing a significant public health challenge that demands comprehensive and sustainable solutions [4].

Numerous nations have proposed various measures and laws to decrease RTCs and prevent fatalities and injuries. In the Netherlands, the Sustainable Safety Outlook was introduced in 1992 as a prime example. Similarly, the British government unveiled its roadmap for enhancing road safety in March 2000, entitled "Roads Tomorrow: Safer for All, outlining their objectives for the next decade" [5]. The United Nations General Assembly passed Resolution 255/64 in March 2010, declaring ten years of action on road safety from 2011 to 2020. International road accident fatalities and injuries were reduced by strengthening national, regional, and global measures. The primary objective was stabilizing and gradually decreasing the situation [6]. The Bloomberg Global Road Safety Program (BIGRS) was launched from 2015 to 2019 to address the significant expenses associated with RTCs in LMICs. This initiative has been implemented in several LMICs, focusing on enforcing national road safety regulations and implementing proven strategies to mitigate road accidents. The ultimate goal is to reduce the number of deaths and injuries resulting from road accidents in LMICs [7].

Developing countries face a lot of road accidents (RTCs), requiring prevention efforts. The attempts to cut down on RTCs are well-organized plans with clear goals and actions to make roads safer and reduce crashes [8]. These RTC prevention strategies can benefit drivers, pedestrians, cyclists, and other road users. Extensive research has been conducted worldwide to assess the effectiveness of RTC reduction measures. Rigorous studies have focused on reducing RTCs in various subpopulations. In a rapid review of systematic studies, legislation is the most common intervention that yields significant results, particularly when combined with solid executive efforts [9,10]. Additionally, graduate driving licenses (GDLs) and other interventions have been found to enhance pedestrian and bicycle visibility and slow down traffic when implemented in a coordinated manner [11–14].

A decline in traffic accident fatalities occurred in 48 middle- and high-income countries between 2013 and 2016 but not in any low-income nation [3]. A comprehensive evaluation and a detailed account of evidence from systematic studies are imperative because many interventions have been implemented and proven effective, while road traffic accidents are continuously increasing. This evaluation should also assess the quality of these studies and identify gaps in evidence to determine the efficacy of interventions. The present study was conducted without restrictions, considering all interventions in all countries on road users. Measures to prevent road traffic accidents encompass legislation, enforcement, public awareness campaigns, driver training, and speed control devices like speed cameras. These interventions can target individuals or groups, including drivers, motorcyclists, cyclists, pedestrians, passengers, and even non-motorized vehicles such as handcarts. Therefore, this study aims to systematically assess the effectiveness of interventions designed to reduce road traffic injuries (RTIs) in low-, middle-, and high-income countries.

## Materials and methods

The research was carefully examined following well-established guidelines for thorough reviews known as PRISMA (Preferred Reporting Items for Systematic Reviews and Meta-Analyses) [15]. This study looked at traffic safety for people at risk on the roads, like pedestrians, drivers, passengers, cyclists, and motorcyclists. People of all ages from around the world, regardless of whether their country is wealthy, moderately wealthy, or less wealthy, were included.

This research proved that preventing accidents helps people who are more at risk on the roads. Based on the World Health Organization’s recommendations for making roads safer, eight main preventive measures included stricter laws, more vigorous enforcement, safer road designs, vehicle improvements, programs to teach people how to stay safe on the roads, educational campaigns, and multiple approaches [16–19]. Moreover, interventions were classified into four groups based on their levels: international, national, subnational, and special group interventions. Subsequently, the outcomes of each intervention were evaluated, considering the time of evaluation. The evaluation was divided into two groups: short-term effects (lasting one year or less) and long-term effects (lasting more than one year).

### Information sources and search strategy

A systematic literature search of PubMed, Web of Science, Google Scholar, Scopus, Embase, PsycInfo, OpenGrey, EconLit, IMEMR, AIM, Cochrane Injuries Group’s specialized register, Transport Research International Documentation (TRID), Transportation Research Information Services (TRIS) Database and the OECD’s Joint Transport Research Centre’s International Transport Research Documentation (ITRD) was performed by two independent authors (M.A. and M.V).

The search used Basic keywords such as Injury, Accident, Traffic, Vehicles, and particular keywords with search operators AND, OR, and NOT. S1 Table demonstrates the search strategy used in PubMed and Scopus databases. This review included all articles published from the first year of publication until December 2023 that met the eligibility. Randomized controlled trials, interrupted time-series studies, controlled before-after studies, pretest/post-test studies, and other epidemiologic studies were considered with a control group.

### Inclusion criteria and study selection

Original studies evaluating the effects of road traffic safety interventions implemented worldwide were included. Articles had to be related to road traffic injury, evaluate a prevention-related intervention, evaluate an outcome (crash, injury, or death), and be peer-reviewed and published in English. Regarding the comparator, studies comparing the intervention to no intervention and other road traffic interventions were considered.

The inclusion criteria included: 1) all articles and reports published in English, 2) articles that contain interventions against road traffic accidents in the world, 3) studies that vulnerable users, including pedestrians, drivers, passengers, cyclists, and motorcyclists, 4) Studies that have obtained the minimum quality score according to systematic review quality assessment tools.

Articles were excluded in the case of being abstracts, literature or systematic reviews, meta-analyses, commentaries, invitro, or simulation studies. Two independent authors (M.A. and M.V.) reviewed selected studies for eligibility, and any discrepancies between reviewers were resolved by consulting a third investigator (KB.L.).

Data were collected from all studies meeting the inclusion criteria on study designs, study populations, characteristics of the intervention and comparison groups, statistical analysis, and outcomes. The World Bank income group classifications are used for the fiscal year 2024, in which 219 countries are stratified by GNI per capita: lower-middle-income economies are those with a GNI per capita between $1,046 and $4,095; upper-middle-income economies are those with a GNI per capita between $4,096 and $12,695; high-income economies are those with a GNI per capita of $12,696 or more [20].

### Quality of studies

The present systematic review included studies of varying designs (randomized control trials, non-randomized interventions, longitudinal studies with stepped wedge designs, interrupted time series, secondary data, or cross-sectional studies with before and after comparisons), so a data quality assessment was performed using a variety of approaches based on the study design: (a) STROBE indicators for reporting observational studies; (b) two scales for non-randomized studies: the ACROBAT-NRS [21] and NOS [22]; (c) Cochrane’s GRADE [23] mechanism for randomized studies; and (d) EPOC suggested risk of bias indicators for interrupted time series studies (EPOC) [24]. The risk of bias (low, moderate, and high risk) was assigned as suggested by the Cochrane Handbook by study design [25]. Studies were classified as high, moderate, and low risk of bias as such: (a) high risk when more than one indicator measure of bias was present across scales; (b) moderate risk when there is one indicator for bias across scales; (c) low risk when all indicators measure low or absence of bias. No studies were excluded from extraction based on the risk of bias, but studies’ contributions to the review results were analyzed in the context of their risk of bias level.

### Data extraction

The primary and secondary outcome measures were the crash, injury, death, change in speeding behaviors, hazard perception, and avoidance due to road traffic crashes. The studies’ general characteristics were gathered, including publication year, study location, intervention type, design type, target population, intervention duration, and outcome type. Furthermore, data on health-related outcome measures, prevention type, prevention impact, and effectiveness as an effect size of outcome were extracted.

In studies of missing data, we made an effort to reach out to the original investigators via email to request complete information whenever possible. In instances where access to the data was not feasible, analyses were conducted solely on the available data.

### Data analysis

Given the significant methodological heterogeneity among the selected studies meaning the differences in study design, analysis methods, and reporting of findings we determined that a traditional meta-analysis was not feasible. Instead, we employed a systematic review approach, which, while sharing some similarities with narrative synthesis, is distinct in its structured aggregation of quantitative findings. In this approach, we systematically categorized and aggregated the quantitative results from the included studies into predefined themes aligned with our study objectives. Specifically, we focused on the effectiveness of interventions in preventing road traffic injuries. Each study’s main results, including the types of interventions and the primary outcomes evaluated, were summarized and organized into these thematic categories.. To quantify the prevalence of specific themes across the studies, we calculated the percentage of studies that addressed each theme by dividing the number of studies within each theme by the total number of included studies. This method allowed us to identify common patterns and the overall impact of different interventions on various outcomes. Finally, the results were organized according to the types of intended interventions and their corresponding outcomes, providing a clear overview of the effectiveness of various interventions in reducing road traffic injuries.

## Results

### Study selection

**Fig 1:**
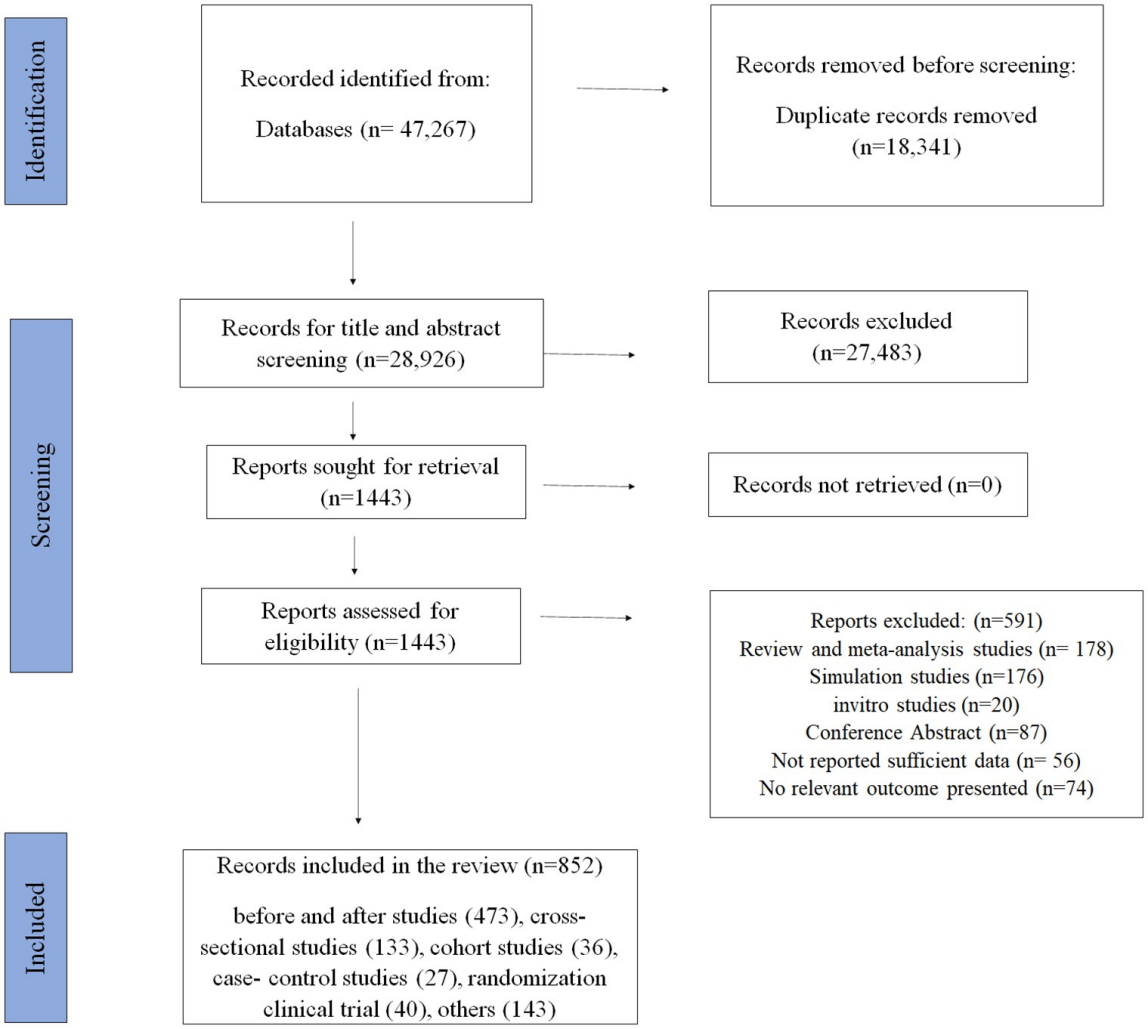
PRISMA Flow diagram; selection of relevant studies via databases.

This review encompasses studies that examined interventions for preventing RTIs worldwide to describe and summarize the findings from effective interventions. Out of 47,267 reports, 1443 full papers were retrieved to assess eligibility after removing 18,341 duplicates and 27,483 irrelevant articles in screening by title and abstract. Finally, 852 articles were included in this systematic review (S1 File). Fig 1 shows the flowchart of the step-by-step article identification and selection process from initial search findings with the reason and number for removing reports (S2 Table).

### Quality assessment of studies

Based on the criteria outlined in the methodology section, 852 studies were evaluated using the relevant checklists. The risk of bias in these studies was categorized as high in 21 studies, moderate in 115 studies, and low in 716 studies (S3 Table).

### Description of included studies

**Fig 2:**
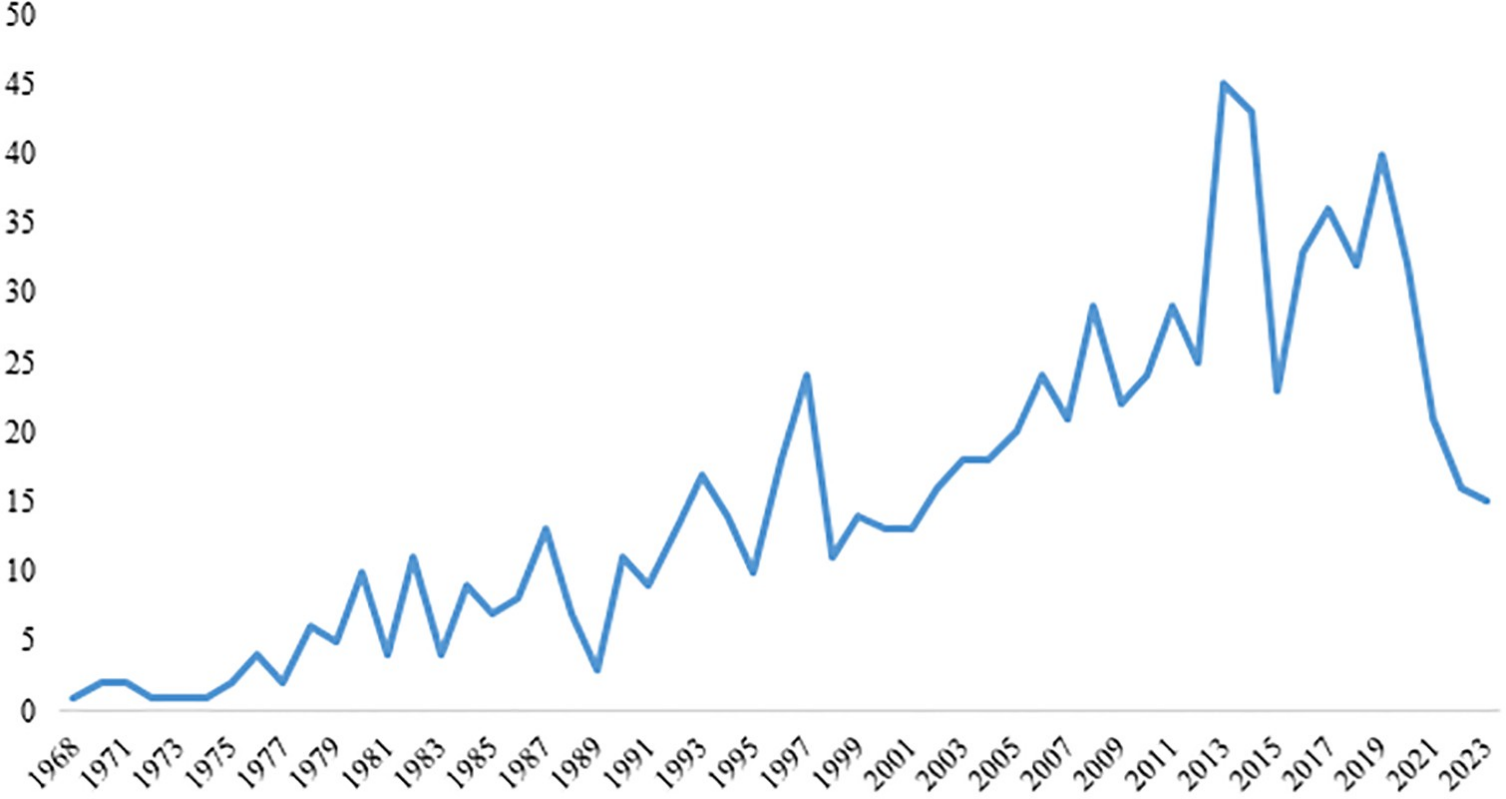
The distribution of published year of articles.

Studies were published between 1968 and 2023, and most of the interventions were implemented during the first decade of road safety action (2013–2014) and then in 2019 (Fig 2).

**Fig 3:**
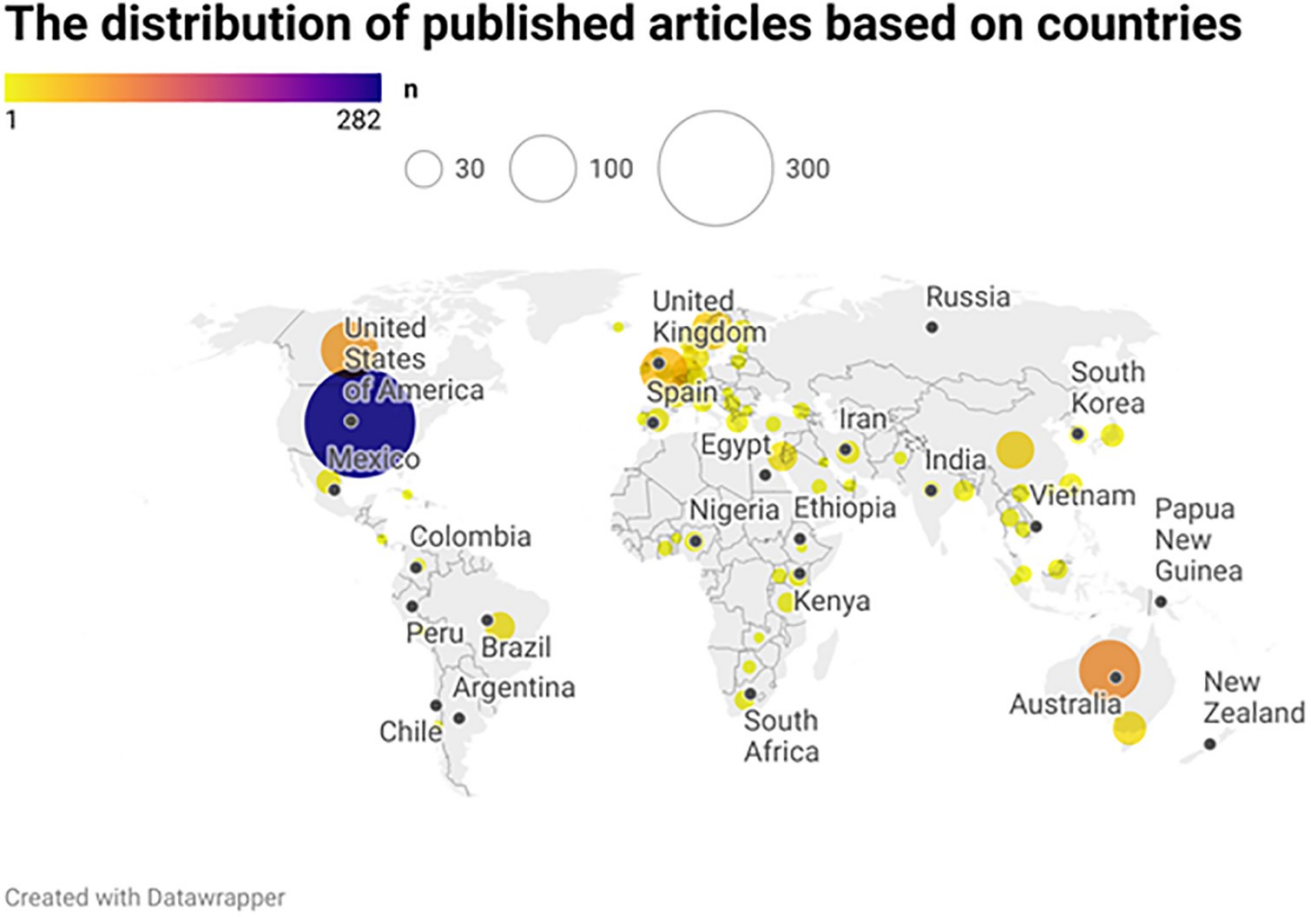
The distribution of published articles based on countries.

Regarding the income level (based on the latest classification of the World Bank—2020), more than half of the interventions (83.7%) took place in developed and high-income countries and then in upper-middle, lower-middle, and low-income countries, respectively (S1 Fig). A total of 351 studies (41.2%) were from North America, 180 (% 21.12) from East Asia and Pacific, 210 (24.6%) from Europe and Central Asia, 34 (4.0%) from Sub-Saharan Africa, 32 (3.8%) from the Middle East, 33 (3.9%) from Latin America and the Caribbean and six from South Asia (Figs 3 and S2).

**Fig 4:**
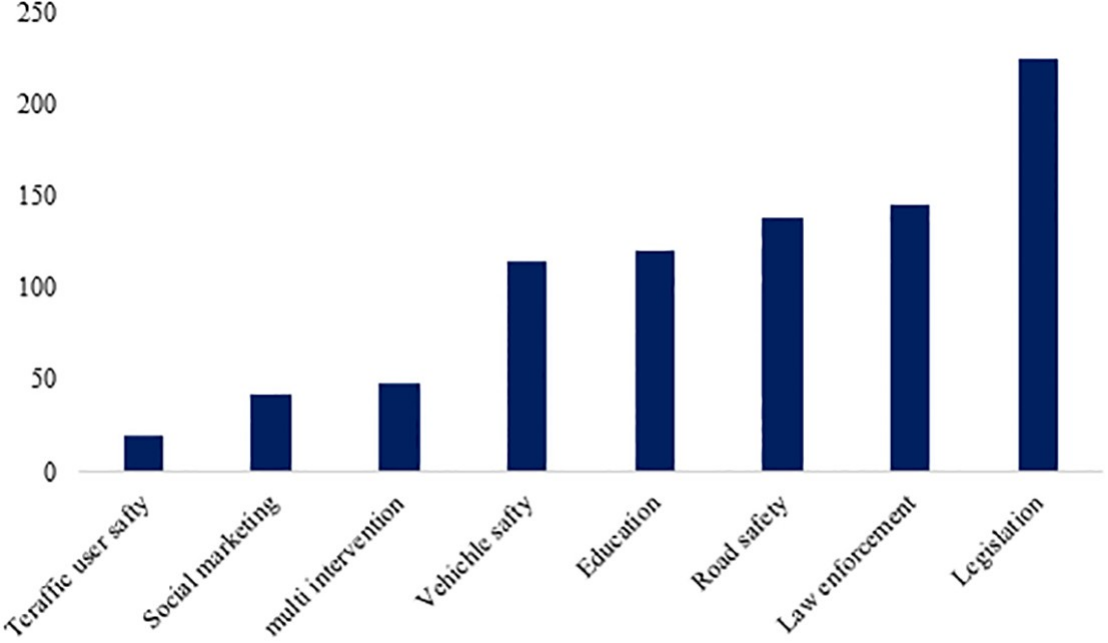
The frequency of articles related to types of interventions.

Eight intervention types were extracted from the 852 included studies (Fig 4). Most interventions were related to legislation (26.4%) and enforcement (17.0%), road safety (16.2%), education (14.1%), vehicle safety (13.5%), then multi intervention (5.6%), social marketing (4.9%) and traffic user safety (2.2%).

Nearly half (45.9%) of the interventions were performed on drivers, while over a fifth (21.4%) were performed on all road users. Pedestrian safety interventions had the lowest share at 4.7%.

The most and the least studies were used before and after non-case-control (55.5%) and case-control studies (3.2%), respectively. In addition, 81.6% of interventions were proven effective in decreasing road safety, and only 18.4% were ineffective.

**Fig 5:**
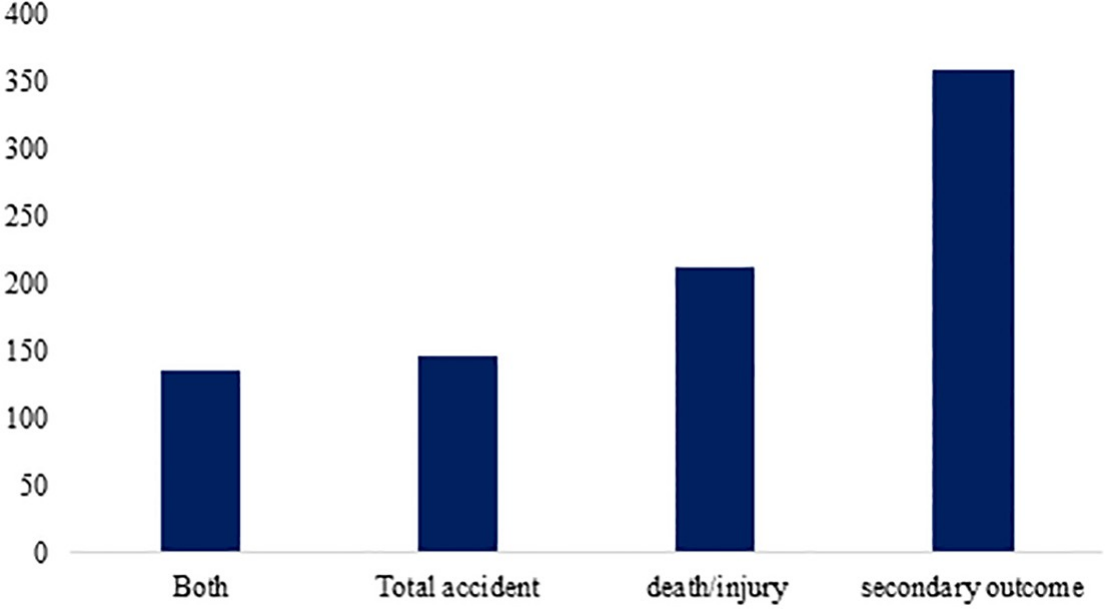
The frequency of articles related to measuring the outcome of interventions.

About 24.9% of studies reported a reduction in mortality or injuries as a direct outcome, 17.1% found a decrease in the total Accidents, and 40.2% revealed a reduction in Secondary Outcomes, such as a change in behaviors, knowledge and attitude, hazard perception, and violations as an indirect outcome (Fig 5).

Regarding the level of intervention, 54.3% were at the sub-national level, 42.3% at the national level, and 3.4% at the international level (S3 Fig).

Concerning intervention effects over time, 60.6% were long-term effects (Over one year), 33.0% were short-term effects (A year or less), and the time of intervention effect was unknown in 6.5% of studies (S4 Fig).

### Effectiveness of interventions

The most common type of prevention was "legislation," with 26.4% of all interventions, followed by "education" with 14.1%, "enforcement" with 17.0%, "traffic user safety" with 2.2%, "social marketing" with 4.9%, "vehicle safety" with 13.5%, "road safety" with 16.2%, and "multiple interventions" with 5.6%. In terms of effectiveness, "legislation" was the most effective type of prevention (26.0%). This was followed by "education" with 14.0%, "road safety" with 16.7%, "enforcement" with 16.7%, "vehicle safety" with 14.0%, "social marketing" with 4.7%, "multiple interventions" with 5.3%, and "traffic user safety" with 2.6%. The least effective type of prevention was "traffic user safety" (0.6%) (P-value = 0.076) (S4 Table).

The results showed that interventions in high-income countries (83.6%) are more effective in reducing RTCs than others, but it was insignificant (P-value = 0.982) (S5 Table).

These studies have the most impact on drivers (45%), followed by all road users (21.2%) and pedestrians (4.5%) (S6 Table). In addition, the effective interventions implemented at the subnational level were more effective (55.3%) than the interventions at the national level (41.4.%) and the international level with 3.3% (P-value = 0.531) (S7 Table).

Most effective interventions (36.1%) were implemented during the Decade of Action for Road Safety (2011–2020) (S8 Table). Further analysis considering both intervention type and year of implementation revealed a shift in focus. Before 1999 and throughout the 2000s, legislation and law enforcement dominated as the most common intervention strategy. However, the focus shifted towards road safety interventions between 2011 and 2020. Since 2020, education has emerged as the predominant intervention type (S9 Table).

The results showed that except for legislation, which had the most significant impact on death/injury (48.4%), among other interventions, law enforcement (35.2%), education (76.7%), road safety (40.6%), and vehicle safety (33.9%) significantly affected the secondary outcome (Table 1).

**Table 1:** Evaluation of the most important factors based on the type of intervention.

Intervention type	Outcome measures	P.value
**Law enforcement**	Secondary outcome(35.2%)	**≤0.001**
**Legislation**	Death/injury(48.4%)	
**Education**	Secondary outcome(76.7%)	
**Road safety**	Secondary outcome(40.6%)	
**Vehicle safety**	Secondary outcome(33.9%)	

## Discussion

Analyzing existing reviews and summarizing past studies is essential in making informed decisions about future research, policy, and practices. This review looked at research that compared various interventions to lower RTCs. Most interventions focused on drivers and road users in all studies. The least number of interventions were performed on pedestrians. The interventions were categorized into three categories based on their overall outcome: effective, non-effective, and multi-outcome. The results showed that most of these interventions effectively reduced traffic accidents, including deaths or injuries, and a few were ineffective. The interventions have reduced traffic accidents, especially in developed countries [26]. However, the study methodology and factors affecting the magnitude of the impact should also be considered along with these effects.

A more detailed analysis of the results of interventions revealed that interventions aimed at reducing all accidents, fatalities, and injuries and those aimed at reducing secondary outcomes, such as changes in perspective or attitude and traffic literacy, were reviewed. Regarding intervention levels, the interventions were classified into four groups: international, national, sub-national, and special groups. However, it is important to note that the focus of traffic safety interventions can differ significantly between developed and developing countries. In developed countries, interventions often focus on issues like drink driving and restraint use, while in developing countries, interventions may prioritize infrastructure improvements and helmet use. This difference in focus stems from the varying road safety challenges and priorities in each context. As a result, directly comparing the effectiveness of interventions across these different settings may not fully capture the complexities involved. Instead, it may be more appropriate to evaluate the effectiveness of interventions within their respective contexts, considering the unique challenges and needs of each region.

According to the present study, past efforts to improve road safety have primarily focused on influencing people’s behavior. Various methods are employed to accomplish this goal, including enforcing traffic laws, enacting new regulations, and promoting education and public awareness [27–29]. There has been no shift in focus from blaming road users to emphasizing how the system is designed despite the "Decade of Action." This means those responsible for designing the system, like policymakers, road planners, law enforcement, politicians, and even healthcare and education professionals, have not been included in efforts to improve road safety [6]. Like previous research, the present study found a surprising absence of robust scientific data to support certain road safety practices, including road infrastructure design and vehicle safety features. Despite decades of efforts, these areas have not seen significant changes based on solid evidence [28].

This research identified another important point: most scientific evidence on road safety in LMICs examines individual interventions in isolation. There was a gap in understanding how these interventions work with other parts of the road transport system. There was also a lack of consideration for the bigger picture in current research instead of looking at isolated components. As a result, underlying factors contributing to accidents were overlooked, and the blame fell solely on road users [30]. However, a system is not just a set of individual components, and road transport systems have the characteristics of complex adaptive systems [31]. A critical part of improving road transport systems was to consider the connections between different parts and stakeholders (stakeholders), how the system behaves (emerging behaviors), and the particular circumstances and history that shape these systems [32]. Individual interventions offered temporary benefits, but these positive effects faded, aligning with the argument by Salmon and Lenne: solely targeting one aspect of the road system without considering how it interacts with others weakens the intervention’s long-term impact [30]. In addition, the effectiveness of individual interventions is reduced unless they are complemented by other interventions. Bambach et al. suggested combining different road safety measures, like guardrails, helmet laws, speed limits, and drunk driving bans, creates a more powerful impact than any single intervention alone [33].

These findings were aligned with past research: wealthier countries produce more scientific evidence on road safety. There is a lack of studies from low-income and lower-middle-income countries. Perel et al. found that only a small fraction (6 out of 236) of reviewed road safety studies addressed these issues [34]. In addition, Zou et al. showed that among the top ten countries that produce 80.56% of the road safety literature, there is only one LMIC–China [35]. This lack of representation may be partly explained by factors related to the research process in LMICs and scientific publishing systems [36]. Understanding how well road safety measures work requires data on accidents. Many low- and middle-income countries lack dependable systems for collecting this crucial information [37]. Another critical factor influencing research is the limited global financial opportunities for road safety. Current funds are granted by donors and dominated by HICs [38]. The lack of LMIC representation is not limited to road safety research. Researchers in these countries also face difficulties getting their work published. A review by the World Health Organization publications found that a very small portion (only 24%) of editors were from low- and middle-income countries, and none were from the poorest countries [39]. This lack of representation could result from institutional racism in the dissemination process [40]. Financial constraints often challenge research from low- and middle-income countries (LMICs), forcing them to publish in journals not included in major Western databases. As a result of this uneven playing field, researchers from wealthier countries (HICs) are unfairly favored in the sharing of research.

As mentioned earlier, the Global Decade Action Plan encourages countries to prioritize vulnerable road users (pedestrians, cyclists, and two- and three-wheelers), who account for half of all road accident deaths worldwide [41]. These users benefit more from speed management, alcohol control, and enhanced vision measures [42]. The present review found that interventions such as lowering the BAC limit, traffic calming measures, traffic signs, and mandatory helmet laws directly assess vulnerable road users. However, no studies evaluated the effectiveness of proven interventions such as alcohol combustion locks [43], street lighting [44], conspicuity aids, and separate bike lanes [45] for vulnerable road users.

The interventions of high-income countries had a more significant impact on the outcome of traffic accidents, although it was not significant. Legislation was the most effective form of prevention. Then, road safety and law enforcement were placed, and vehicle safety and education were the most effective interventions. The least effective type of prevention was the safety of traffic users. Traffic accidents can be effectively reduced when the legislation is strictly enforced. For example, mandatory seat belt laws, drunk driving bans, speed limits, and child seat requirements have been shown to reduce the number and severity of traffic accidents [46,47]. However, legislation alone may not be sufficient to prevent all traffic accidents. Factors such as individual behavior, attitudes, and risk perception also play an essential role. For example, driving under the influence of alcohol, despite legal penalties, is a significant risk factor for road accidents [48]. Vehicle safety is essentially a matter for car manufacturers. Car manufacturers can affect vehicle safety by designing the vehicle and the other is installing the appropriate safety equipment. Some safety equipment, such as child seats, may later be purchased separately by the car user. Vehicle design and safety equipment cover two types or areas of safety solutions. Some are related to active solutions that reduce the number of accidents in situations where an accident is possible. Vehicle rear sensors and anti-lock brakes are in this category. Passive or reactive solutions such as seat belts and airbags reduce the severity of injuries in the event of an accident [49]. Today, road safety is decreasing with the increasing growth of vehicles, especially in developing countries, and the statistics for accidents and human casualties show an expanding growth. Road defects are one of the reasons for accidents. This defect indicates that the executors in the stages of feasibility, design, construction, and operation of road projects have not observed the cases and standards related to road safety, which forms accident-prone sections and points in the national road network [50].

In addition, the interventions implemented at the subnational level have significantly impacted reducing traffic accidents more than other cases. Therefore, increasing vehicle safety, Road safety, and law enforcement have significantly reduced secondary outcomes. Legislation has played the most significant role in reducing death/injury, but education in traffic accidents has had a more significant impact on changing attitudes, literacy levels, and risk perception (as secondary consequences). The most effective is law enforcement in the target group of drivers.

Global studies have shown that three factors-humans, roads and vehicles- are recognized as the main components of road accidents. Among these, man is the most unknown and essential factor in events to the extent that some studies attribute more than 70% of accidents to this factor [51–53]. Six interventions are listed in the WHO list for effective and proven interventions that reduce traffic accidents and the frequency and severity of injuries resulting from traffic accidents. Of these six interventions, four are related to human factors, such as the permitted speed limit, helmet use, and safety equipment for children. These interventions can only be effective through legislation and law enforcement [1,54]. According to a 2013 WHO report on the importance and role of legislation in preventing traffic accidents, legislation is an essential component of a comprehensive road safety strategy that aims to reduce injuries and deaths and achieve overall road safety goals [55].

As mentioned, most of the interventions were conducted on drivers due to the essential role of drivers in traffic accidents from a human point of view, prioritizing them to implement interventions and fundamental principles in traffic accident prevention policies.

There were some limitations in this study. In specific analyses, the number of participants in each group was small, meaning the results might not be reliable. While focusing on low- and middle-income countries (LMICs) provided valuable insights but presented a challenge. LMICs encompass various situations and factors that could influence our topic. However, the term "LMIC" was used to highlight these countries’ general trends and emphasize the lack of research comparing them to wealthier nations.

## Conclusion

A common mistake is to study different parts of the road system in isolation without considering their interaction. Most efforts focus on changing driver behavior rather than making roads safer. Moving from blaming drivers to fixing the whole system is the best way to understand the entire system. Getting good accident data requires collaboration with relevant parties and improving data collection. The lack of data systems in some countries can be overcome by combining information from different sources and using new methods to ensure the missing data is accounted for. Modern tools can also improve how road deaths are recorded in low- and middle-income countries [56]. Closing the research gap in low-income countries (LICs) is essential, but it will take time. Building research networks that include LICs is crucial. The scientific community needs to do more to support research from LIC scientists by offering funding and helping them publish their findings. Global road safety efforts are entering their second decade, and our study can help create safe, inclusive, and sustainable communities worldwide. A holistic "systems thinking" approach is needed instead of focusing on individual components. Road safety in LICs depends on studying the entire road system, identifying the players involved, and improving regional, national, and local accountability.

## Supporting information

S1 Fig The frequency of articles related to level income. (DOCX)

S2 Fig Regional distribution (continent). (DOCX)

S3 Fig The frequency of articles related to level interventions. (DOCX)

S4 Fig The frequency of articles related to time interventions. (DOCX)

S1 Table Search strategy. (DOCX)

S2 Table Reasons for exclusion. (DOCX)

S3 Table Risk of bias findings. (DOCX)

S4 Table Relationship between prevention type and intervention outcomes (Chi Square Test). (DOCX)

S5 Table Relationship between level income and intervention outcomes (Chi Square Test). (DOCX)

S6 Table Relationship between target population and intervention outcomes (Chi Square Test). (DOCX)

S7 Table Relationship between Level of implementation and intervention outcomes (Chi Square Test). (DOCX)

S8 Table Relationship between year of the study and intervention outcomes (Chi Square Test). (DOCX)

S9 Table Relationship between year of the study and intervention type (Chi Square Test). (DOCX)

S1 File Data availability as a supplementry file. (XLSX)
