# Supplementary material for: Effectiveness of interventions for preventing road traffic injuries: A systematic review in low-, middle- and high-income countries
Source: PLoS One. 2024 Dec 5;19(12):e0312428. doi: 10.1371/journal.pone.0312428 (PMC11620428; doi:10.1371/journal.pone.0312428)
Supplement: S1 Table — (DOCX) [file pone.0312428.s005.docx]

**S1 Table. Search Strategy**

PubMed database

| **Set** | **Strategy** | **Results** | **#** |
| --- | --- | --- | --- |
| **#1** | "Accidents, Traffic"[Mesh] OR (("Motor Vehicles"[Mesh:NoExp] OR "Automobiles"[Mesh] OR "Motorcycles"[Mesh] OR "[cyclist](https://www.google.com/search?q=cyclist&tbm=isch&tbo=u&source=univ&sa=X&ved=0ahUKEwjpp8nSksPaAhVEVSwKHZhSDpEQsAQIVQ) "[Mesh] OR traffic[tiab] OR vehicle[tiab] OR vehicular[tiab] OR car[tiab] OR cars[tiab] OR automobile[tiab] OR automobiles[tiab] OR motorcycle[tiab] OR motorcycles[tiab] OR taxi[tiab] OR cab[tiab] cycle[tiab] OR bicycle[tiab] OR bike[tiab] OR cyclist[tiab] OR road[tiab] OR pedestrian[tiab] OR pedestrians[tiab]) AND (accident[tiab] OR accidents[tiab] OR injury[tiab] OR injuries[tiab] OR "Wounds and Injuries"[Mesh] OR "injuries" [Subheading])) | **base** | **47754** |
| **#2** | “Developed Countries”[Mesh] OR "Latin America"[Mesh] OR "Europe"[Mesh] OR "Central Asia"[Mesh] OR "Caribbean"[Mesh] OR "Andorra"[Mesh] OR "Antigua"[Mesh] OR "Barbuda"[Mesh] OR "Aruba"[Mesh] OR "Australia"[Mesh] OR "Austria"[Mesh] OR "Bahamas"[Mesh] OR "Bahrain"[Mesh] OR "Barbados"[Mesh] OR "Belgium" [Mesh] OR "Bermuda"[Mesh] OR "British"[Mesh] OR "Brunei"[Mesh] OR "Canada"[Mesh] OR "Cayman Islands"[Mesh] OR "Channel Islands"[Mesh] OR "Chile"[Mesh] OR "Croatia"[Mesh] OR "Curaçao"[Mesh] OR "Cyprus"[Mesh] OR "Czech Republic"[Mesh] OR "Denmark"[Mesh] OR "Estonia"[Mesh] OR "Faroe Islands"[Mesh] OR "France"[Mesh] OR "Palau"[Mesh] OR "French Polynesia"[Mesh] OR "Germany"[Mesh] OR "Gibraltar"[Mesh] OR "Greece"[Mesh] OR "Greenland"[Mesh] OR "Guam"[Mesh] OR "Hong Kong"[Mesh] OR "Hungary"[Mesh] OR "Iceland"[Mesh] OR "Ireland"[Mesh] OR "Isle of Man"[Mesh] OR "Israel"[Mesh] OR "Italy"[Mesh] OR "Japan"[Mesh] OR "Korea"[Mesh] OR "Kuwait"[Mesh] OR "Latvia"[Mesh] OR "Liechtenstein"[Mesh] OR "Lithuania"[Mesh] OR "Luxembourg" [Mesh] OR "Macau"[Mesh] OR "Malta"[Mesh] OR "Monaco"[Mesh] OR "Netherlands"[Mesh] OR "New Caledonia"[Mesh] OR "New Zealand"[Mesh] OR "Norway"[Mesh] OR "Oman"[Mesh] OR "Poland Portugal"[Mesh] OR "Puerto Rico"[Mesh] OR "Qatar"[Mesh] OR "San Marino"[Mesh] OR "Saudi Arabia"[Mesh] OR "Seychelles"[Mesh] OR "Singapore"[Mesh] OR "Sint Maarten"[Mesh] OR "Slovak Republic"[Mesh] OR "Slovenia"[Mesh] OR "Spain"[Mesh] OR "Martin"[Mesh] OR " Sweden"[Mesh] OR "Switzerland"[Mesh] OR "Taiwan"[Mesh] OR "Trinidad"[Mesh] OR "Turks"[Mesh] OR "United Arab"[Mesh] OR "Emirates"[Mesh] OR "United Kingdom"[Mesh] OR "United States"[Mesh] OR "Uruguay "[Mesh] OR "Virgin Islands "[Mesh] OR "Latin America"[tiab] OR "Europe"[tiab] OR "Middle East"[tiab] OR "North Africa"[ tiab] OR "Central Asia"[tiab] OR "Caribbean"[tiab] OR "Andorra"[tiab] OR "Antigua"[tiab] OR "Barbuda"[tiab] OR " Aruba"[tiab] OR "Australia"[tiab] OR "Austria"[tiab] OR "Bahamas"[tiab] OR "Bahrain"[tiab] OR "Barbados"[tiab] OR "Belgium" [tiab] OR "Bermuda"[tiab] OR "British"[tiab] OR "Brunei"[tiab] OR "Canada"[tiab] OR "Cayman Islands"[tiab] OR "Channel Islands"[tiab] OR "Chile"[tiab] OR "Croatia"[tiab] OR "Curaçao"[tiab] OR "Cyprus"[tiab] OR "Czech Republic"[tiab] OR "Denmark"[tiab] OR "Estonia"[tiab] OR "Faroe Islands"[tiab] OR "France"[tiab] OR "Palau"[tiab] OR "French Polynesia"[tiab] OR "Germany"[tiab] OR "Gibraltar"[tiab] OR "Greece"[tiab] OR "Greenland"[tiab] OR "Guam"[tiab] OR "Hong Kong"[tiab] OR "Hungary"[tiab] OR "Iceland"[tiab] OR "Ireland "[tiab] OR "Isle of Man"[tiab] OR "Israel"[tiab] OR "Italy"[tiab] OR "Japan"[tiab] OR "Korea"[tiab] OR "Kuwait"[tiab] OR "Latvia"[tiab] OR "Liechtenstein"[tiab] OR "Lithuania"[tiab] OR "Luxembourg" [tiab] OR "Macau"[tiab] OR "Malta"[tiab] OR "Monaco"[tiab] OR "Netherlands"[tiab] OR "New Caledonia"[tiab] OR "New Zealand"[tiab] OR "Norway"[tiab] OR "Oman"[tiab] OR "Poland Portugal"[tiab] OR "Puerto Rico"[tiab] OR "Qatar"[tiab] OR "San Marino"[tiab] OR "Saudi Arabia"[tiab] OR "Seychelles"[tiab] OR "Singapore"[tiab] OR "Sint Maarten"[tiab] OR "Slovak Republic"[tiab] OR "Slovenia"[tiab] OR "Spain"[tiab] OR "Martin"[tiab] OR "Sweden"[tiab] OR "Switzerland"[tiab] OR "Taiwan"[tiab] OR "Trinidad"[tiab] OR "Turks"[tiab] OR "United Arab"[tiab] OR "Emirates"[tiab] OR "United Kingdom"[tiab] OR "United States"[tiab] OR "Uruguay "[tiab] OR "Virgin Islands "[tiab] OR "high resource"[tiab] OR "Developed Nation"[tiab] OR "Industrialized Nations"[tiab] OR "high-developed"[tiab] OR "developed country"[tiab] OR (high[tiab] AND income[tiab]) | **High income** | **446264** |
| **#3** | “Developing Countries”[Mesh] OR "Middle East"[Mesh] OR "Albania"[Mesh] OR "Algeria"[Mesh] OR "American Samoa"[Mesh] OR "Angola"[Mesh] OR "Nauru"[Mesh] OR "Tonga"[Mesh] OR "Argentina"[Mesh] OR "Azerbaijan"[Mesh] OR "Belarus"[Mesh] OR "Belize"[Mesh] OR "Samoa"[Mesh] OR "Bosnia"[Mesh] OR "Botswana"[Mesh] OR "Brazil"[Mesh] OR "Bulgaria"[Mesh] OR "China"[Mesh] OR "Colombia"[Mesh] OR "Costa Rica"[Mesh] OR "Cuba"[Mesh] OR "Dominica"[Mesh] OR "Dominican Republic"[Mesh] OR "Equatorial Guinea"[Mesh] OR "Ecuador"[Mesh] OR "Fiji"[Mesh] OR "Gabon"[Mesh] OR "Georgia"[Mesh] OR "Grenada"[Mesh] OR "Jamaica"[Mesh] OR "Jordan"[Mesh] OR "Kazakhstan"[Mesh] OR "Guyana"[Mesh] OR "Iran"[Mesh] OR "Iraq"[Mesh] OR "Lebanon"[Mesh] OR "Libya"[Mesh] OR "Macedonia"[Mesh] OR "Malaysia"[Mesh] OR "Maldives"[Mesh] OR "Marshall Islands"[Mesh] OR "Mauritius"[Mesh] OR "Mexico"[Mesh] OR "Montenegro"[Mesh] OR "Namibia"[Mesh] OR "Panama"[Mesh] OR "Paraguay"[Mesh] OR "Peru"[Mesh] OR "Romania"[Mesh] OR "Russian Federation"[Mesh] OR "Serbia"[Mesh] OR "South Africa"[Mesh] OR "Lucia"[Mesh] OR "Vincent" [Mesh] OR "Suriname"[Mesh] OR "Thailand"[Mesh] OR "Turkey"[Mesh] OR "Turkmenistan"[Mesh] OR "Tuvalu"[Mesh] OR "Venezuela"[Mesh] OR “Developing Countries”[tiab] OR "Middle East"[tiab] OR "Albania"[tiab] OR "Algeria"[tiab] OR "American Samoa"[tiab] OR "Angola"[tiab] OR "Nauru"[tiab] OR "Tonga"[tiab] OR "Argentina"[tiab] OR "Azerbaijan"[tiab] OR "Belarus"[tiab] OR "Belize"[tiab] OR "Samoa"[tiab] OR "Bosnia"[tiab] OR "Botswana"[tiab] OR "Brazil"[tiab] OR "Bulgaria"[tiab] OR "China"[tiab] OR "Colombia"[tiab] OR "Costa Rica"[tiab] OR "Cuba"[tiab] OR "Dominica"[tiab] OR "Dominican Republic"[tiab] OR "Equatorial Guinea"[tiab] OR "Ecuador"[tiab] OR "Fiji"[tiab] OR "Gabon"[tiab] OR "Georgia"[tiab] OR "Grenada"[tiab] OR "Jamaica"[tiab] OR "Jordan"[tiab] OR "Kazakhstan"[tiab] OR "Guyana "[tiab] OR "Iran "[tiab] OR "Iraq"[tiab] OR "Lebanon"[tiab] OR "Libya"[tiab] OR "Macedonia"[tiab] OR "Malaysia"[tiab] OR "Maldives"[tiab] OR "Marshall Islands"[tiab] OR "Mauritius"[tiab] OR "Mexico"[tiab] OR "Montenegro"[tiab] OR "Namibia"[tiab] OR "Panama"[tiab] OR "Paraguay"[tiab] OR "Peru"[tiab] OR "Romania"[tiab] OR "Russian Federation"[tiab] OR "Serbia"[tiab] OR "South Africa"[tiab] OR "Lucia"[tiab] OR "Vincent"[tiab] OR "Suriname"[tiab] OR "Thailand"[tiab] OR "Turkey"[tiab] OR "Turkmenistan"[tiab] OR "Tuvalu"[tiab] OR "Venezuela"[tiab] OR "Developing Nation"[tiab] OR "Upper-middle"[tiab] OR "developing country"[tiab] OR (Upper-middle[tiab] AND income[tiab]) | **Upper-middle-income** | **1319256** |
|  | “Developing Countries”[Mesh] OR "Armenia"[Mesh] OR "Bangladesh"[Mesh] OR " Bhutan"[Mesh] OR "Bolivia"[Mesh] OR "Cabo Verde"[Mesh] OR "Cambodia"[Mesh] OR "Cameroon"[Mesh] OR "Congo"[Mesh] OR "Djibouti"[Mesh] OR "Egypt"[Mesh] OR "El Salvador"[Mesh] OR "Ghana"[Mesh] OR "Guatemala"[Mesh] OR "Honduras"[Mesh] OR "India"[Mesh] OR "Indonesia"[Mesh] OR "Kenya"[Mesh] OR "Kiribati"[Mesh] OR "Kosovo"[Mesh] OR "Kyrgyz"[Mesh] OR "Lao"[Mesh] OR "Lesotho"[Mesh] OR "Mauritania"[Mesh] OR "Micronesia"[Mesh] OR "Moldova"[Mesh] OR "Mongolia"[Mesh] OR "Morocco"[Mesh] OR "Myanmar"[Mesh] OR "Nicaragua"[Mesh] OR "Nigeria"[Mesh] OR "Pakistan"[Mesh] OR "Papua New"[Mesh] OR "Philippines"[Mesh] OR "Sao Tome"[Mesh] OR "Solomon Islands"[Mesh] OR "Sri Lanka"[Mesh] OR "Sudan"[Mesh] OR "Swaziland"[Mesh] OR "Syrian Arab Republic"[Mesh] OR "Tajikistan"[Mesh] OR "Timor Leste"[Mesh] OR "Tunisia"[Mesh] OR "Ukraine"[Mesh] OR "Uzbekistan"[Mesh] OR "Vanuatu"[Mesh] OR "Vietnam"[Mesh] OR "Gaza"[Mesh] OR "Yemen"[Mesh] OR "Zambia"[Mesh] OR “Developing Countries”[tiab] OR "Armenia"[tiab] OR "Bangladesh"[tiab] OR "Bhutan"[tiab] OR "Bolivia"[tiab] OR "Cabo Verde"[tiab] OR "Cambodia"[tiab] OR "Cameroon"[tiab] OR "Congo"[tiab] OR "Djibouti"[tiab] OR "Egypt"[tiab] OR "El Salvador"[tiab] OR "Ghana"[tiab] OR "Guatemala"[tiab] OR "Honduras"[tiab] OR "India"[tiab] OR "Indonesia"[tiab] OR "Kenya"[tiab] OR "Kiribati"[tiab] OR "Kosovo"[tiab] OR "Kyrgyz"[tiab] OR "Lao"[tiab] OR "Lesotho"[tiab] OR "Mauritania"[tiab] OR "Micronesia"[tiab] OR "Moldova"[tiab] OR "Mongolia"[tiab] OR "Morocco"[tiab] OR "Myanmar"[tiab] OR "Nicaragua"[tiab] OR "Nigeria"[tiab] OR "Pakistan"[tiab] OR "Papua New"[tiab] OR "Philippines"[tiab] OR "Sao Tome"[tiab] OR "Solomon Islands"[tiab] OR "Sri Lanka"[tiab] OR "Sudan"[tiab] OR "Swaziland"[tiab] OR "Syrian Arab Republic"[tiab] OR "Tajikistan "[tiab] OR "Timor Leste"[tiab] OR "Tunisia"[tiab] OR "Ukraine"[tiab] OR "Uzbekistan"[tiab] OR "Vanuatu"[tiab] OR "Vietnam"[tiab] OR "Gaza"[tiab] OR "Yemen"[tiab] OR "Zambia"[tiab] OR "Lower-middle Country"[tiab] OR (Lower-middle[tiab] AND income[tiab])  “South Asia”[Mesh] OR "Afghanistan"[Mesh] OR "Benin"[Mesh] OR "Burkina"[Mesh] OR "Faso"[Mesh] OR "Burundi"[Mesh] OR "Central African Republic"[Mesh] OR "Chad"[Mesh] OR "Comoros"[Mesh] OR "Congo"[Mesh] OR "Eritrea"[Mesh] OR "Ethiopia"[Mesh] OR "Gambia"[Mesh] OR "Guinea-Bissau"[Mesh] OR "Haiti"[Mesh] OR "Korea"[Mesh] OR "Liberia"[Mesh] OR "Madagascar"[Mesh] OR "Malawi"[Mesh] OR "Mali"[Mesh] OR "Mozambique"[Mesh] OR "Nepal"[Mesh] OR "Niger"[Mesh] OR "Rwanda"[Mesh] OR "Senegal"[Mesh] OR "Sierra Leone"[Mesh] OR "Somalia"[Mesh] OR "South Sudan"[Mesh] OR "Tanzania"[Mesh] OR "Togo"[Mesh] OR "Uganda"[Mesh] OR "Zimbabwe"[Mesh] OR "South Asia"[tiab] OR "Afghanistan"[tiab] OR "Benin"[tiab] OR "Burkina"[tiab] OR "Faso"[tiab] OR "Burundi"[tiab] OR "Central African Republic"[tiab] OR "Chad"[tiab] OR "Comoros"[tiab] OR "Congo"[tiab] OR "Eritrea"[tiab] OR "Ethiopia"[tiab] OR "Gambia"[tiab] OR "Guinea-Bissau"[tiab] OR "Haiti"[tiab] OR "Korea"[tiab] OR "Liberia"[tiab] OR "Madagascar"[tiab] OR "Malawi"[tiab] OR "Mali"[tiab] OR "Mozambique"[tiab] OR "Nepal"[tiab] OR "Niger"[tiab] OR "Rwanda"[tiab] OR "Senegal"[tiab] OR "Sierra Leone"[tiab] OR "Somalia"[tiab] OR "South Sudan"[tiab] OR "Tanzania"[tiab] OR "Togo"[tiab] OR "Uganda"[tiab] OR "Zimbabwe"[tiab] OR "under-resourced"[tiab] OR "resource poor"[tiab] OR "under-developed"[tiab] OR "underdeveloped"[tiab] OR "Low resource "[tiab] OR (Low[tiab] AND income[tiab]) | **Low income** | **409137** |
|  | #1 AND ( #2 OR #3 OR #4) |  | **8012** |

Scopus database

| Set | Strategy | Results | # |
| --- | --- | --- | --- |
| #1 | TITLE-ABS-KEY( (("Motor Vehicles" OR Automobiles OR Motorcycles OR traffic OR vehicle OR vehicular OR car OR cars OR automobile OR motorcycle OR taxi OR cab bicycle OR bike OR cyclist OR road OR pedestrian OR pedestrians) AND (accident OR accidents OR injury OR injuries))) | base | 57225 |
| #2 | TITLE-ABS-KEY (“Developed Countries” OR "Latin America" OR "Europe" OR "Central Asia" OR "Caribbean" OR "Andorra" OR "Antigua" OR "Barbuda" OR "Aruba" OR "Australia" OR "Austria" OR "Bahamas" OR "Bahrain" OR "Barbados" OR "Belgium" OR "Bermuda" OR "British" OR "Brunei" OR "Canada" OR "Cayman Islands" OR "Channel Islands" OR "Chile" OR "Croatia"] OR "Curaçao" OR "Cyprus" OR "Czech Republic" OR "Denmark" OR "Estonia" OR "Faroe Islands" OR "France" OR "Palau" OR "French Polynesia" OR "Germany" OR "Gibraltar" OR "Greece" OR "Greenland" OR "Guam" OR "Hong Kong" OR "Hungary" OR "Iceland" OR "Ireland" OR "Isle of Man" OR "Israel" OR "Italy" OR "Japan" OR "Korea" OR "Kuwait" OR "Latvia" OR "Liechtenstein" OR "Lithuania" OR "Luxembourg" OR "Macau" OR "Malta" OR "Monaco" OR "Netherlands" OR "New Caledonia" OR "New Zealand" OR "Norway" OR "Oman" OR "Poland Portugal" OR "Puerto Rico" OR "Qatar" OR "San Marino" OR "Saudi Arabia" OR "Seychelles" OR "Singapore" OR "Sint Maarten" OR "Slovak Republic" OR "Slovenia" OR "Spain" OR "Martin" OR "Sweden" OR "Switzerland" OR "Taiwan" OR "Trinidad" OR "Turks" OR "United Arab" OR "Emirates" OR "United Kingdom" OR "United States" OR "Uruguay " OR "Virgin Islands " OR "high resource" OR "Developed Nation" OR "Industrialized Nations" OR "high-developed" OR "developed country" OR (high AND income)( | High income | 9,223,286 |
| #3 | TITLE-ABS-KEY (“Developing Countries” OR "Middle East" OR "Albania" OR "Algeria" OR "American Samoa" OR "Angola" OR "Nauru" OR "Tonga" OR "Argentina" OR "Azerbaijan" OR "Belarus" OR "Belize" OR "Samoa" OR "Bosnia" OR "Botswana" OR "Brazil" OR "Bulgaria" OR "China" OR "Colombia" OR "Costa Rica" OR "Cuba" OR "Dominica" OR "Dominican Republic" OR "Equatorial Guinea" OR "Ecuador" OR "Fiji" OR "Gabon" OR "Georgia" OR "Grenada" OR "Jamaica" OR "Jordan" OR "Kazakhstan" OR "Guyana" OR "Iran" OR "Iraq" OR "Lebanon" OR "Libya" OR "Macedonia" OR "Malaysia" OR "Maldives" OR "Marshall Islands" OR "Mauritius" OR "Mexico" OR "Montenegro" OR "Namibia" OR "Panama" OR "Paraguay" OR "Peru" OR "Romania" OR "Russian Federation" OR "Serbia" OR "South Africa" OR "Lucia" OR "Vincent" OR "Suriname" OR "Thailand" OR "Turkey" OR "Turkmenistan" OR "Tuvalu" OR "Venezuela" OR "Developing Nation" OR "Upper-middle" OR "developing country" OR (Upper-middle AND income)) | Upper-middle-income | 4,016,787 |
|  | TITLE-ABS-KEY ("Developing Countries" OR "Armenia" OR "Bangladesh" OR "Bhutan" OR "Bolivia" OR "Cabo Verde" OR "Cambodia" OR "Cameroon" OR "Congo" OR "Djibouti" OR "Egypt" OR "El Salvador" OR "Ghana" OR "Guatemala" OR "Honduras" OR "India" OR "Indonesia" OR "Kenya" OR "Kiribati" OR "Kosovo" OR "Kyrgyz" OR "Lao" OR "Lesotho" OR "Mauritania" OR "Micronesia" OR "Moldova" OR "Mongolia" OR "Morocco" OR "Myanmar" OR "Nicaragua" OR "Nigeria" OR "Pakistan" OR "Papua New" OR "Philippines" OR "Sao Tome" OR "Solomon Islands" OR "Sri Lanka" OR "Sudan" OR "Swaziland" OR "Syrian Arab Republic" OR "Tajikistan" OR "Timor Leste" OR "Tunisia" OR "Ukraine" OR "Uzbekistan" OR "Vanuatu" OR "Vietnam" OR "Gaza" OR "Yemen" OR "Zambia" OR "Lower-middle Country" OR (Lower-middle AND income)) |  | 2,021,676 |
|  | #1 AND ( #2 OR #3 OR #4) |  | 22,406 |
