# Supplementary material for: Effectiveness of interventions for preventing road traffic injuries: A systematic review in low-, middle- and high-income countries
Source: PLoS One. 2024 Dec 5;19(12):e0312428. doi: 10.1371/journal.pone.0312428 (PMC11620428; doi:10.1371/journal.pone.0312428)
Supplement: S2 Table — (DOCX) [file pone.0312428.s006.docx]

| **S2 Table. Reasons for Exclusion** | |
| --- | --- |
| **Reports excluded: (n=591)** | **References** |
| Review and meta-analysis studies: (n= 178) | [1-178] |
| Simulation studies: (n= 176) | [179-354] |
| Invitro studies: (n= 20) | [355-374] |
| Conference Abstract: (n= 87) | [375-461] |
| Not reported sufficient data: (n= 56) | [462-517] |
| No relevant outcome presented: (n= 74) | [518-591] |

**References:**

1. Grime G. A review of research on the protection afforded to occupants of cars by seat belts which provide upper torso restraint. Accident Analysis & Prevention. 1979;11(4):293-306.

2. Mann RE, Leigh G, Vingilis ER, De Genova K. A critical review on the effectiveness of drinking-driving rehabilitation programmes. Accident Analysis & Prevention. 1983;15(6):441-61.

3. Lund AK, Williams AF. A review of the literature evaluating the defensive driving course. Accident Analysis & Prevention. 1985;17(6):449-60.

4. Struckman-Johnson DL, Lund AK, Williams AF, Osborne DW. Comparative effects of driver improvement programs on crashes and violations. Accident Analysis & Prevention. 1989;21(3):203-15.

5. Elvik R. Meta-analysis of evaluations of public lighting as accident countermeasure. Transportation Research Record. 1995;1485(1):12-24.

6. Elvik R. The safety value of guardrails and crash cushions: a meta-analysis of evidence from evaluation studies. Accident Analysis & Prevention. 1995;27(4):523-49.

7. WELLS‐PARKER E, BANGERT‐DROWNS R, McMillen R, Williams M. Final results from a meta‐analysis of remedial interventions with drink/drive offenders. Addiction. 1995;90(7):907-26.

8. Dowswell T, Towner E, Simpson G, Jarvis S. Preventing childhood unintentional injuries--what works? A literature review. Injury Prevention. 1996;2(2):140-9.

9. Elvik R. A meta-analysis of studies concerning the safety effects of daytime running lights on cars. Accident Analysis & Prevention. 1996;28(6):685-94.

10. Forjuoh SN, Guohua L. A review of successful transport and home injury interventions to guide developing countries. Social science & medicine. 1996;43(11):1551-60.

11. Siskind V. Does license disqualification reduce reoffence rates? Accident Analysis & Prevention. 1996;28(4):519-24.

12. Thompson DC, Rivara F, Thompson R, Group CI. Helmets for preventing head and facial injuries in bicyclists. Cochrane database of systematic reviews. 1996;2010(1).

13. Charman WN. Vision and driving‐a literature review and commentary. Ophthalmic and Physiological Optics. 1997;17(5):371-91.

14. Hagenzieker MP, Bijleveld FD, Davidse RJ. Effects of incentive programs to stimulate safety belt use: a meta-analysis. Accident Analysis & Prevention. 1997;29(6):759-77.

15. Jonah BA. Sensation seeking and risky driving: a review and synthesis of the literature. Accident Analysis & Prevention. 1997;29(5):651-65.

16. Dinh-Zarr T, Diguiseppi C, Heitman E, Roberts I. Preventing injuries through interventions for problem drinking: a systematic review of randomized controlled trials. Alcohol and Alcoholism. 1999;34(4):609-21.

17. Elvik R. The effects on accidents of studded tires and laws banning their use: a meta-analysis of evaluation studies. Accident Analysis & Prevention. 1999;31(1-2):125-34.

18. Zwerling C, Jones MP. Evaluation of the effectiveness of low blood alcohol concentration laws for younger drivers. American Journal of Preventive Medicine. 1999;16(1):76-80.

19. Attewell RG, Glase K, McFadden M. Bicycle helmet efficacy: a meta-analysis. Accident Analysis & Prevention. 2001;33(3):345-52.

20. Elvik R. Area-wide urban traffic calming schemes: a meta-analysis of safety effects. Accident Analysis & Prevention. 2001;33(3):327-36.

21. Mann RE, Macdonald S, Stoduto G, Bondy S, Jonah B, Shaikh A. The effects of introducing or lowering legal per se blood alcohol limits for driving: an international review. Accident Analysis & Prevention. 2001;33(5):569-83.

22. Zaza S, Sleet DA, Thompson RS, Sosin DM, Bolen JC, Services TFoCP. Reviews of evidence regarding interventions to increase use of child safety seats. American journal of preventive medicine. 2001;21(4):31-47.

23. Duperrex O, Bunn F, Roberts I. Safety education of pedestrians for injury prevention: a systematic review of randomised controlled trials. Bmj. 2002;324(7346):1129.

24. Bunn F, Collier T, Frost C, Ker K, Roberts I, Wentz R. Traffic calming for the prevention of road traffic injuries: systematic review and meta-analysis. Injury prevention. 2003;9(3):200-4.

25. Bunn F, Collier T, Frost C, Ker K, Roberts I, Wentz R. Area-wide traffic calming for preventing traffic related injuries (Cochrane Review). The Cochrane Library. 2003;(1).

26. Curnow WJ. The efficacy of bicycle helmets against brain injury. Accident Analysis & Prevention. 2003;35(2):287-92.

27. Forjuoh SN. Traffic-related injury prevention interventions for low-income countries. Injury control and safety promotion. 2003;10(1-2):109-18.

28. Morrison DS, Petticrew M, Thomson H. What are the most effective ways of improving population health through transport interventions? Evidence from systematic reviews. Journal of Epidemiology & Community Health. 2003;57(5):327-33.

29. Elder RW, Shults RA, Sleet DA, Nichols JL, Thompson RS, Rajab W, et al. Effectiveness of mass media campaigns for reducing drinking and driving and alcohol-involved crashes: a systematic review. American journal of preventive medicine. 2004;27(1):57-65.

30. Hartling L, Wiebe N, Russell KF, Petruk J, Spinola C, Klassen TP. Graduated driver licensing for reducing motor vehicle crashes among young drivers. Cochrane Database of Systematic Reviews. 2004;(2).

31. Kwan I, Mapstone J. Visibility aids for pedestrians and cyclists: a systematic review of randomised controlled trials. Accident Analysis & Prevention. 2004;36(3):305-12.

32. Ogilvie D, Egan M, Hamilton V, Petticrew M. Promoting walking and cycling as an alternative to using cars: systematic review. Bmj. 2004;329(7469):763.

33. Sassani A, Findley LJ, Kryger M, Goldlust E, George C, Davidson TM. Reducing motor-vehicle collisions, costs, and fatalities by treating obstructive sleep apnea syndrome. Sleep. 2004;27(3):453-8.

34. Shults RA, Elder RW, Sleet DA, Thompson RS, Nichols JL. Primary enforcement seat belt laws are effective even in the face of rising belt use rates. Accident Analysis & Prevention. 2004;36(3):491-3.

35. Shults RA, Nichols JL, Dinh-Zarr TB, Sleet DA, Elder RW. Effectiveness of primary enforcement safety belt laws and enhanced enforcement of safety belt laws: a summary of the Guide to Community Preventive Services systematic reviews. Journal of Safety Research. 2004;35(2):189-96.

36. Van Driel CJ, Davidse RJ, van Maarseveen MF. The effects of an edgeline on speed and lateral position: a meta-analysis. Accident Analysis & Prevention. 2004;36(4):671-82.

37. Waters H, Hyder AA, Phillips T. Economic evaluation of interventions to reduce road traffic injuries-a review of the literature with applications to low and middie-income countries. Asia Pacific Journal of Public Health. 2004;16(1):23-31.

38. A Fletcher KM, S D Baulk, D Dawson. Countermeasures to driver fatigue: a review of public awareness campaigns and legal approaches. Australian and New Zealand journal of public health. 2005;29(5):471-6.

39. A S Aeron-Thomas SH. Red-light cameras for the prevention of road traffic crashes. The Cochrane database of systematic reviews. 2005;18(2).

40. Blais E, Dupont B. Assessing the capability of intensive police programmes to prevent severe road accidents: A systematic review. British Journal of Criminology. 2005;45(6):914-37.

41. Curnow WJ. The Cochrane Collaboration and bicycle helmets. Accident Analysis & Prevention. 2005;37(3):569-73.

42. Ditter SM, Elder RW, Shults RA, Sleet DA, Compton R, Nichols JL. Effectiveness of designated driver programs for reducing alcohol-impaired driving: a systematic review. American journal of preventive medicine. 2005;28(5):280-7.

43. Elder RW, Nichols JL, Shults RA, Sleet DA, Barrios LC, Compton R. Effectiveness of school-based programs for reducing drinking and driving and riding with drinking drivers: A systematic review. American journal of preventive medicine. 2005;28(5):288-304.

44. Elvik R, Greibe P. Road safety effects of porous asphalt: a systematic review of evaluation studies. Accident Analysis & Prevention. 2005;37(3):515-22.

45. Ker K, Roberts I, Collier T, Beyer F, Bunn F, Frost C. Post-licence driver education for the prevention of road traffic crashes: a systematic review of randomised controlled trials. Accident Analysis & Prevention. 2005;37(2):305-13.

46. Pilkington P, Kinra S. Effectiveness of speed cameras in preventing road traffic collisions and related casualties: systematic review. Bmj. 2005;330(7487):331-4.

47. Spinks A, Turner C, McClure R, Acton C, Nixon J. Community-based programmes to promote use of bicycle helmets in children aged 0–14 years: a systematic review. International journal of injury control and safety promotion. 2005;12(3):131-42.

48. Tippetts AS, Voas RB, Fell JC, Nichols JL. A meta-analysis of. 08 BAC laws in 19 jurisdictions in the United States. Accident Analysis & Prevention. 2005;37(1):149-61.

49. Turner C, McClure R, Nixon J, Spinks A. Community-based programs to promote car seat restraints in children 0–16 years–a systematic review. Accident Analysis & Prevention. 2005;37(1):77-83.

50. Dragutinovic N, Twisk D. The effectiveness of road safety education: A literature review. SWOV Institute for Road Safety Research. 2006.

51. Ehiri JE, Ejere HO, Hazen AE, Emusu D, King WD, Osberg SJ. Interventions to increase children’s booster seat use: a review. American journal of preventive medicine. 2006;31(2):185-92.

52. Karkhaneh M, Kalenga J-C, Hagel BE, Rowe BH. Effectiveness of bicycle helmet legislation to increase helmet use: a systematic review. Injury Prevention. 2006;12(2):76-82.

53. Kwan I, Mapstone J. Interventions for increasing pedestrian and cyclist visibility for the prevention of death and injuries. Cochrane Database of Systematic Reviews. 2006;(4).

54. Stallard P, Velleman R, Salter E, Howse I, Yule W, Taylor G. A randomised controlled trial to determine the effectiveness of an early psychological intervention with children involved in road traffic accidents. Journal of Child Psychology and Psychiatry. 2006;47(2):127-34.

55. Wilson C, Willis C, Hendrikz JK, Bellamy N. Speed enforcement detection devices for preventing road traffic injuries. Cochrane Database of Systematic Reviews. 2006;(2).

56. Dellinger AM, Sleet DA, Shults RA, Rinehart CF. Interventions to prevent motor vehicle injuries. Handbook of injury and violence prevention: Springer; 2007. p. 55-79.

57. Kua A, Korner-Bitensky N, Desrosiers J, Man-Son-Hing M, Marshall S. Older driver retraining: A systematic review of evidence of effectiveness. Journal of Safety Research. 2007;38(1):81-90.

58. Man‐Son‐Hing M, Marshall SC, Molnar FJ, Wilson KG. Systematic review of driving risk and the efficacy of compensatory strategies in persons with dementia. Journal of the American Geriatrics Society. 2007;55(6):878-84.

59. Royal S, Kendrick D, Coleman T. Promoting bicycle helmet wearing by children using non-legislative interventions: systematic review and meta-analysis. Injury Prevention. 2007;13(3):162-7.

60. Caird JK, Willness CR, Steel P, Scialfa C. A meta-analysis of the effects of cell phones on driver performance. Accident Analysis & Prevention. 2008;40(4):1282-93.

61. Desapriya E, Subzwari S, Scime G, Pike I. The effectiveness of intervention strategies to reduce motor vehicle crashes involving older drivers: systematic reviews and meta-analyses. 2008.

62. Erke A. Effects of electronic stability control (ESC) on accidents: A review of empirical evidence. Accident Analysis & Prevention. 2008;40(1):167-73.

63. Goss CW, Van Bramer LD, Gliner JA, Porter TR, Roberts IG, DiGuiseppi C. Increased police patrols for preventing alcohol‐impaired driving. Cochrane Database of Systematic Reviews. 2008;(4).

64. Liu BC, Ivers R, Norton R, Boufous S, Blows S, Lo SK. Helmets for preventing injury in motorcycle riders. Cochrane database of systematic reviews. 2008;(1).

65. Small K. Interventions to prevent adolescent motor vehicle crashes: A literature review. Orthopaedic nursing. 2008;27(5):283-90.

66. Beyer FR, Ker K. Street lighting for preventing road traffic injuries. Cochrane database of systematic reviews. 2009;(1).

67. Erke A. Red light for red-light cameras?: A meta-analysis of the effects of red-light cameras on crashes. Accident Analysis & Prevention. 2009;41(5):897-905.

68. Erke A, Goldenbeld C, Vaa T. The effects of drink-driving checkpoints on crashes—A meta-analysis. Accident Analysis & Prevention. 2009;41(5):914-23.

69. Hatfield J, Murphy S, Job RS, Du W. The effectiveness of audio-tactile lane-marking in reducing various types of crash: a review of evidence, template for evaluation, and preliminary findings from Australia. Accident Analysis & Prevention. 2009;41(3):365-79.

70. Novoa AM, Pérez K, Borrell C. Evidence-based effectiveness of road safety interventions: a literature review. Gaceta Sanitaria. 2009;23(6):553. e1-14.

71. Reynolds CC, Harris MA, Teschke K, Cripton PA, Winters M. The impact of transportation infrastructure on bicycling injuries and crashes: a review of the literature. Environmental health. 2009;8:1-19.

72. Subzwari S, Desapriya E, Babul‐Wellar S, Pike I, Turcotte K, Rajabali F, et al. Vision screening of older drivers for preventing road traffic injuries and fatalities. Cochrane database of systematic reviews. 2009;(1).

73. E Desapriya SS, D Sasges, A Basic, A Alidina, K Turcotte, I Pike. Do light truck vehicles (LTV) impose greater risk of pedestrian injury than passenger cars? A meta-analysis and systematic review. Traffic injury prevention. 2010;11(1):48-56.

74. Høye A. Are airbags a dangerous safety measure? A meta-analysis of the effects of frontal airbags on driver fatalities. Accident Analysis & Prevention. 2010;42(6):2030-40.

75. Jorge M Rodríguez-Hernández JCC-R. Primary prevention measures for controlling pedestrian injuries and deaths and improving road safety. Revista de salud pública. 2010;12(3):497-509.

76. Katina Kardamanidis AM, Rebecca Q Ivers, Mark R Stevenson, Katrina Thistlethwaite. Motorcycle rider training for the prevention of road traffic crashes. The Cochrane database of systematic reviews. 2010;6(10):5240.

77. Krahl PL, Jankosky CJ, Thomas RJ, Hooper TI. Systematic review of military motor vehicle crash–related injuries. American journal of preventive medicine. 2010;38(1):S189-S96.

78. Wilson C, Willis C, Hendrikz JK, Le Brocque R, Bellamy N. Speed cameras for the prevention of road traffic injuries and deaths. Cochrane database of systematic reviews. 2010;(11).

79. Abbas AK, Hefny AF, Abu-Zidan FM. Seatbelts and road traffic collision injuries. World journal of emergency surgery. 2011;6:1-6.

80. Antonopoulos CN, Sergentanis TN, Daskalopoulou SS, Petridou ET. Nasal continuous positive airway pressure (nCPAP) treatment for obstructive sleep apnea, road traffic accidents and driving simulator performance: a meta-analysis. Sleep medicine reviews. 2011;15(5):301-10.

81. Elder RW, Voas R, Beirness D, Shults RA, Sleet DA, Nichols JL, et al. Effectiveness of ignition interlocks for preventing alcohol-impaired driving and alcohol-related crashes: a Community Guide systematic review. American journal of preventive medicine. 2011;40(3):362-76.

82. Elvik R. Publication bias and time-trend bias in meta-analysis of bicycle helmet efficacy: a re-analysis of Attewell, Glase and McFadden, 2001. Accident Analysis & Prevention. 2011;43(3):1245-51.

83. Phillips RO, Ulleberg P, Vaa T. Meta-analysis of the effect of road safety campaigns on accidents. Accident Analysis & Prevention. 2011;43(3):1204-18.

84. Skorga P, Young C. Motorcycle rider training for the prevention of road traffic crashes: A review synopsis. Public health nursing. 2011;28(5).

85. Meuser TM, Berg-Weger M, Niewoehner PM, Harmon AC, Kuenzie JC, Carr DB, et al. Physician input and licensing of at-risk drivers: a review of all-inclusive medical evaluation forms in the US and Canada. Accident Analysis & Prevention. 2012;46:8-17.

86. Polinder S, Segui-Gomez M, Toet H, Belt E, Sethi D, Racioppi F, et al. Systematic review and quality assessment of economic evaluation studies of injury prevention. Accident Analysis & Prevention. 2012;45:211-21.

87. Sai Ma QL, Maigeng Zhou, Leilei Duan, David Bishai. Road traffic injury in China: a review of national data sources. Traffic injury prevention. 2012;13:57-63.

88. Dugan E, Barton KN, Coyle C, Lee CM. US policies to enhance older driver safety: a systematic review of the literature. Journal of Aging & Social Policy. 2013;25(4):335-52.

89. Høye A. Still red light for red light cameras? An update. Accident Analysis & Prevention. 2013;55:77-89.

90. Marino M, De Belvis A, Basso D, Avolio M, Pelone F, Tanzariello M, et al. Interventions to evaluate fitness to drive among people with chronic conditions: systematic review of literature. Accident Analysis & Prevention. 2013;50:377-96.

91. Martin AJ, Marottoli R, O'neill D. Driving assessment for maintaining mobility and safety in drivers with dementia. Cochrane database of systematic reviews. 2013;(5).

92. Soole DW, Watson BC, Fleiter JJ. Effects of average speed enforcement on speed compliance and crashes: A review of the literature. Accident Analysis & Prevention. 2013;54:46-56.

93. Strand MC, Fjeld B, Arnestad M, Mørland J. Can patients receiving opioid maintenance therapy safely drive? A systematic review of epidemiological and experimental studies on driving ability with a focus on concomitant methadone or buprenorphine administration. Traffic Injury Prevention. 2013;14(1):26-38.

94. Bonaccorsi G, Porchia B, Baldasseroni A, Dellisanti C, Lorini C. A systematic review on the effectiveness of two interventions in preventing traffic accidents: Guglielmo Bonaccorsi. European Journal of Public Health. 2014;24(suppl_2):cku166-46.

95. Høye A. Speed cameras, section control, and kangaroo jumps–a meta-analysis. Accident Analysis & Prevention. 2014;73:200-8.

96. Ishikawa T, Oudie E, Desapriya E, Turcotte K, Pike I. A systematic review of community interventions to improve Aboriginal child passenger safety. American journal of public health. 2014;104(S3):e1-e8.

97. Megan M Short CJM, Michel Bédard. Interventions for motor vehicle crashes among Indigenous communities: strategies to inform Canadian initiatives. Canadian journal of public health. 2014;30(4):e296-305.

98. Pidd K, Roche AM. How effective is drug testing as a workplace safety strategy? A systematic review of the evidence. Accident Analysis & Prevention. 2014;71:154-65.

99. Porchia BR, Baldasseroni A, Dellisanti C, Lorini C, Bonaccorsi G. Effectiveness of two interventions in preventing traffic accidents: a systematic review. Ann Ig. 2014;26(1):63-75.

100. Richmond SA, Zhang YJ, Stover A, Howard A, Macarthur C. Prevention of bicycle-related injuries in children and youth: a systematic review of bicycle skills training interventions. Injury prevention. 2014;20(3):191-5.

101. Rothman L, Buliung R, Macarthur C, To T, Howard A. Walking and child pedestrian injury: a systematic review of built environment correlates of safe walking. Injury prevention. 2014;20(1):41-9.

102. Schwebel DC, Barton BK, Shen J, Wells HL, Bogar A, Heath G, et al. Systematic review and meta-analysis of behavioral interventions to improve child pedestrian safety. Journal of pediatric psychology. 2014;39(8):826-45.

103. Unsworth CA, Baker A. Driver rehabilitation: A systematic review of the types and effectiveness of interventions used by occupational therapists to improve on-road fitness-to-drive. Accident Analysis & Prevention. 2014;71:106-14.

104. Walter R Boot CS, Neil Charness. Improving the safety of aging road users: a mini-review. Gerontology. 2014;60(1):90-6.

105. Allan Lyckegaard TH, Inger Marie Bernhoft. Effectiveness of electronic stability control on single-vehicle accidents. Traffic injury prevention. 2015;16(4):380-6.

106. Cairns J, Warren J, Garthwaite K, Greig G, Bambra C. Go slow: an umbrella review of the effects of 20 mph zones and limits on health and health inequalities. Journal of public health. 2015;37(3):515-20.

107. Cohn E, Kakar S, Farrington D. Red Light Camera Interventions for Reducing Traffic Violations and Accidents: A Systematic. 2015.

108. Green RS, Kureshi N, Erdogan M. Legal consequences for alcohol-impaired drivers injured in motor vehicle collisions: A systematic review. Accident Analysis & Prevention. 2015;80:106-16.

109. John S Decker SJS, Benjamin McManus, Shannon M O Wittig, Virginia P Sisiopiku, Despina Stavrinos. The impact of billboards on driver visual behavior: a systematic literature review. Traffic injury prevention. 2015;16:234-39.

110. Mulvaney CA, Smith S, Watson MC, Parkin J, Coupland C, Miller P, et al. Cycling infrastructure for reducing cycling injuries in cyclists. Cochrane database of systematic reviews. 2015;(12).

111. Roshandel S, Zheng Z, Washington S. Impact of real-time traffic characteristics on freeway crash occurrence: Systematic review and meta-analysis. Accident Analysis & Prevention. 2015;79:198-211.

112. Yadav R-P, Kobayashi M. A systematic review: effectiveness of mass media campaigns for reducing alcohol-impaired driving and alcohol-related crashes. BMC public health. 2015;15:1-17.

113. Brieger F, Hagen R, Vetter D, Dormann CF, Storch I. Effectiveness of light-reflecting devices: A systematic reanalysis of animal-vehicle collision data. Accident Analysis & Prevention. 2016;97:242-60.

114. Craig A, Tran Y, Guest R, Gopinath B, Jagnoor J, Bryant RA, et al. Psychological impact of injuries sustained in motor vehicle crashes: systematic review and meta-analysis. BMJ open. 2016;6(9):e011993.

115. Ekmejian R, Sarrami P, Naylor JM, Harris IA. A systematic review on the effectiveness of back protectors for motorcyclists. Scandinavian journal of trauma, resuscitation and emergency medicine. 2016;24:1-6.

116. Salam RA, Arshad A, Das JK, Khan MN, Mahmood W, Freedman SB, et al. Interventions to prevent unintentional injuries among adolescents: a systematic review and meta-analysis. Journal of Adolescent Health. 2016;59(4):S76-S87.

117. Staton C, Vissoci J, Gong E, Toomey N, Wafula R, Abdelgadir J, et al. Road traffic injury prevention initiatives: a systematic review and metasummary of effectiveness in low and middle income countries. PloS one. 2016;11(1):e0144971.

118. Zatoński M, Herbeć A. Are mass media campaigns effective in reducing drinking and driving? Systematic review–an update. Journal of Health Inequalities. 2016;2(1):52-60.

119. Araujo M, Illanes E, Chapman E, Rodrigues E. Effectiveness of interventions to prevent motorcycle injuries: systematic review of the literature. International journal of injury control and safety promotion. 2017;24(3):406-22.

120. Asbridge M, Desapriya E, Ogilvie R, Cartwright J, Mehrnoush V, Ishikawa T, et al. The impact of restricted driver’s licenses on crash risk for older drivers: a systematic review. Transportation Research Part A: Policy and Practice. 2017;97:137-45.

121. Banstola A, Mytton J. Cost-effectiveness of interventions to prevent road traffic injuries in low-and middle-income countries: A literature review. Traffic injury prevention. 2017;18(4):357-62.

122. Chihuri S, Li G. Use of prescription opioids and motor vehicle crashes: a meta analysis. Accident Analysis & Prevention. 2017;109:123-31.

123. Elvik R. Road safety effects of roundabouts: A meta-analysis. Accident Analysis & Prevention. 2017;99:364-71.

124. Irwin C, Iudakhina E, Desbrow B, McCartney D. Effects of acute alcohol consumption on measures of simulated driving: a systematic review and meta-analysis. Accident Analysis & Prevention. 2017;102:248-66.

125. Nazari SSH, Moradi A, Rahmani K. A systematic review of the effect of various interventions on reducing fatigue and sleepiness while driving. Chinese journal of traumatology. 2017;20(5):249-58.

126. Peng Y, Vaidya N, Finnie R, Reynolds J, Dumitru C, Njie G, et al. Universal motorcycle helmet laws to reduce injuries: a community guide systematic review. American journal of preventive medicine. 2017;52(6):820-32.

127. Simmons SM, Caird JK, Steel P. A meta-analysis of in-vehicle and nomadic voice-recognition system interaction and driving performance. Accident Analysis & Prevention. 2017;106:31-43.

128. Theofilatos A, Ziakopoulos A, Papadimitriou E, Yannis G, Diamandouros K. Meta-analysis of the effect of road work zones on crash occurrence. Accident Analysis & Prevention. 2017;108:1-8.

129. Asbridge M, Ogilvie R, Wilson M, Hayden J. The impact of booster seat use on child injury and mortality: Systematic review and meta-analysis of observational studies of booster seat effectiveness. Accident Analysis & Prevention. 2018;119:50-7.

130. Azevedo SB, de Vasconcelos CMR, Leal LP, de Vasconcelos MGL. Educational interventions on prevention of accidents in preschool children: integrative review. 2018.

131. Bonnet E, Lechat L, Ridde V. What interventions are required to reduce road traffic injuries in Africa? A scoping review of the literature. PLoS One. 2018;13(11):e0208195.

132. Clavljo GC, Untlveros CB, Ñopo PC, Escobedo RC, Ramírez EA, Sánchez KH, et al., editors. Politicas e intervenciones para reducir lesiones por accidentes de transito: De la evidencia a la practica/Policies and interventions to reduce injuries due to traffic accidents: From evidence to practice. Anales de la facultad de medicina; 2018: Universidad Nacional Mayor de San Marcos.

133. Hoye A. Recommend or mandate? A systematic review and meta-analysis of the effects of mandatory bicycle helmet legislation. Accident Analysis & Prevention. 2018;120:239-49.

134. Høye A. Bicycle helmets–To wear or not to wear? A meta-analyses of the effects of bicycle helmets on injuries. Accident Analysis & Prevention. 2018;117:85-97.

135. Larouche R, Mammen G, Rowe DA, Faulkner G. Effectiveness of active school transport interventions: a systematic review and update. BMC public health. 2018;18:1-18.

136. Lefio Á, Bachelet VC, Jiménez-Paneque R, Gomolán P, Rivas K. A systematic review of the effectiveness of interventions to reduce motor vehicle crashes and their injuries among the general and working populations. Revista panamericana de salud publica. 2018;42:e60.

137. Saber Azami-Aghdash HS-B, Mahdiyeh Heydari , Ramin Rezapour , Naser Derakhshani Effectiveness of Interventions for Prevention of Road Traffic Injuries in Iran and Some Methodological Issues: A Systematic Review. Bulletin of emergency and trauma. 2018;6(2):90-9.

138. Sanchez-Ramirez DC, Voaklander D. The impact of policies regulating alcohol trading hours and days on specific alcohol-related harms: a systematic review. Injury prevention. 2018;24(1):94-100.

139. Schreier DR, Banks C, Mathis J. Driving simulators in the clinical assessment of fitness to drive in sleepy individuals: A systematic review. Sleep medicine reviews. 2018;38:86-100.

140. Hosseini J, Khorasani-Zavareh D, Abdoli M, Shariatpanahi S, Lotfi S, Nematollahi S. Primary preventive interventions on traffic accidents among males: A review on the literature during 2009-2019 in Iran. Men's Health Journal. 2019;3(1):e11-e.

141. Koohi F, Soori H. Helmet use and its efficacy on preventing motorcycle injuries: A systematic review and meta-analysis. Journal of Mazandaran University of Medical Sciences. 2019;28(168):198-216.

142. Koppel S, Bugeja L, Hua P, Osborne R, Stephens AN, Young KL, et al. Do mindfulness interventions improve road safety? A systematic review. Accident Analysis & Prevention. 2019;123:88-98.

143. Anna Tupetz KF, Duan Zhao 3, Huipeng Liao , Megan Von Isenburg , Elizabeth M Keating , Joao Ricardo Nickenig Vissoci , Catherine A Staton. Prevention of childhood unintentional injuries in low- and middle-income countries: A systematic review. PLoS One. 2020;15(12):e0243464.

144. Azami-Aghdash S. Meta-synthesis of qualitative evidence in road traffic injury prevention: a scoping review of qualitative studies (2000 to 2019). Archives of public health. 2020;78:1-27.

145. Bjørn Olsson HP, Fabian Reitzug , David K Humphreys. Evaluating the impact of penalising the use of mobile phones while driving on road traffic fatalities, serious injuries and mobile phone use: a systematic review. Injury prevention. 2020;26(4):378-85.

146. Cohn EG, Kakar S, Perkins C, Steinbach R, Edwards P. Red light camera interventions for reducing traffic violations and traffic crashes: A systematic review. Campbell systematic reviews. 2020;16(2):e1091.

147. Du RY, LoPresti MA, García RM, Lam S. Primary prevention of road traffic accident–related traumatic brain injuries in younger populations: a systematic review of helmet legislation. Journal of Neurosurgery: Pediatrics. 2020;25(4):361-74.

148. Fell JC, Scolese J, Achoki T, Burks C, Goldberg A, DeJong W. The effectiveness of alternative transportation programs in reducing impaired driving: A literature review and synthesis. Journal of safety research. 2020;75:128-39.

149. Gupta M, Bandyopadhyay S. Regulatory and road engineering interventions for preventing road traffic injuries and fatalities among vulnerable road users in low-and middle-income countries: a systematic review. Frontiers in Sustainable Cities. 2020;2:10.

150. Selveindran SM, Tango T, Khan MM, Simadibrata DM, Hutchinson PJ, Brayne C, et al. Mapping global evidence on strategies and interventions in neurotrauma and road traffic collisions prevention: a scoping. trauma. 2020;37:38.

151. Shafina Shafiq SD, Noor Kutubul Alam Siddiquee , Meghnath Dhimal , Anjani Kumar Jha Existing Laws to Combat Road Traffic Injury in Nepal and Bangladesh: A Review on Cross Country Perspective. Journal of Nepal Health Research Council. 2020;17(4):416-23.

152. Akbari M, Lankarani KB, Tabrizi R, Heydari ST, Vali M, Motevalian SA, et al. The effectiveness of mass media campaigns in increasing the use of seat belts: a systematic review. Traffic injury prevention. 2021;22(7):495-500.

153. Akbari M, Lankarani KB, Tabrizi R, Vali M, Heydari ST, Motevalian SA, et al. The effect of motorcycle safety campaign on helmet use: A systematic review and meta-analysis. IATSS research. 2021;45(4):513-20.

154. Gifty G, Zubair SM, Poobalan A, Sumit K. Effective interventions in road traffic accidents among the young and novice drivers of low and middle-income countries: A scoping review. Clinical epidemiology and global health. 2021;12:100865.

155. Lucci C, Piantini S, Savino G, Pierini M. Motorcycle helmet selection and usage for improved safety: a systematic review on the protective effects of helmet type and fastening. Traffic injury prevention. 2021;22(4):301-6.

156. Luis Miguel Martín-delosReyes PL-C, Laura García-Cuerva , Mario Rivera-Izquierdo , Eladio Jiménez-Mejías , Virginia Martínez-Ruiz. Effect of Periodic Vehicle Inspection on Road Crashes and Injuries: A Systematic Review. International journal of environmental research and public health. 2021;15:6476.

157. Maryam Akbari a KBLa, *, Seyed Taghi Heydari a , Seyed Abbas Motevalian b , Reza Tabrizi c , Mark JMSullman d. Is driver education contributing towards road safety? a systematic review of systematic reviews. Journal of injury and violence research. 2021;13(1):69.

158. Razaghizad A, Windle SB, Gore G, Benedetti A, Ells C, Grad R, et al. Interventions to prevent drugged driving: a systematic review. American journal of preventive medicine. 2021;61(2):267-80.

159. Abdi N, Robertson T, Petrucka P, Crizzle AM. Do motorcycle helmets reduce road traffic injuries, hospitalizations and mortalities in low and lower-middle income countries in Africa? A systematic review and meta-analysis. BMC public health. 2022;22(1):824.

160. Bou-Karroum L, El-Jardali F, Jabbour M, Harb A, Fadlallah R, Hemadi N, et al. Preventing unintentional injuries in school-aged children: a systematic review. Pediatrics. 2022;149(Supplement 6).

161. Fisa R, Musukuma M, Sampa M, Musonda P, Young T. Effects of interventions for preventing road traffic crashes: an overview of systematic reviews. BMC public health. 2022;22(1):513.

162. listed Na. Assessment of road safety intervention implementation in Benin in 2019. Sante Publique. 2022;33(5):763-78.

163. Namatovu S, Balugaba BE, Muni K, Ningwa A, Nsabagwa L, Oporia F, et al. Interventions to reduce pedestrian road traffic injuries: A systematic review of randomized controlled trials, cluster randomized controlled trials, interrupted time-series, and controlled before-after studies. PLoS one. 2022;17(1):e0262681.

164. Rezapur-Shahkolai F, Afshari M, Doosti-Irani A, Bashirian S, Maleki S. Interventions to prevent road traffic injuries among pedestrians: a systematic review. International journal of injury control and safety promotion. 2022;29(4):533-49.

165. Saadati M, Razzaghi A, Rezapour R, Pourebrahim K. Interventions for safety promotion of pedestrians; A scoping review. Journal of Transport & Health. 2022;24:101277.

166. Salar Mohammaddokht MS, Babak Kashefimehr , Saber Azami-Aghdash Assessing and Improving Fitness to Drive in the Elderly People: A Scoping Review of Policies and Guidelines. Iranian journal of public health. 2022;51(11):2304-16.

167. Sarah B Windle PS, José Ignacio Nazif-Munoz , Sam Harper , Arijit Nandi. The Impact of Cannabis Decriminalization and Legalization on Road Safety Outcomes: A Systematic Review. American journal of preventive medicine. 2022;63(6):1037-52.

168. Tello KT, Hughey SM, Porto SC, Hart M, Benson A. Interventions to improve pedestrian and cyclist safety in Latin America: a systematic review and metasummary. Journal of healthy eating and active living. 2022;2(2):45.

169. Urrechaga EM, Kodadek LM, Bugaev N, Bauman ZM, Shah KH, Aziz HA, et al. Full-face motorcycle helmets to reduce injury and death: a systematic review, meta-analysis, and practice management guideline from the Eastern Association for the Surgery of Trauma. The American Journal of Surgery. 2022;224(5):1238-46.

170. Baker CE, Yu X, Patel S, Ghajari M. A review of cyclist head injury, impact characteristics and the implications for helmet assessment methods. Annals of biomedical engineering. 2023;51(5):875-904.

171. Büth CM, Barbour N, Abdel-Aty M. Effectiveness of bicycle helmets and injury prevention: a systematic review of meta-analyses. Scientific reports. 2023;13(1):8540.

172. Gudugbe S, Yeboah DK, Konadu P, Awoonor-Williams R, Clegg-Lamptey JNA, Rahman GA, et al. Approaches to the Effective Prevention of Road Traffic Injuries in Sub-Saharan Africa: A Systematic Review. Open Journal of Social Sciences. 2023;11(2):323-44.

173. Jahangiry L, Eisazadeh S, Khabiri R, Sadeghi-Bazargani H, Bakhtari-Aghdam F, Ponnet K. Health promotion interventions on helmet use: a systematic review and meta-analysis of pre-test and post-test studies. Iranian journal of public health. 2023;52(9):1866.

174. Kamran B Lankarani MA, Alireza Razzaghi, Seyed Taghi Heydari, Mohebat Vali, Reza Tabrizi, Mark JM Sullman. Mass media campaigns to increase the use of bicycle helmets: a systematic review and meta-analysis. Journal of Transport & Health. 2023;30:101616.

175. Morgan CH, Stager LM, Schwebel DC, Shen J. A systematic review and meta-analysis on the efficacy of virtual reality pedestrian interventions to teach children how to cross streets safely. Journal of pediatric psychology. 2023;48(12):1003-20.

176. Nowiński M, Sak J, Fus-Mazurkiewicz L, Mazurkiewicz W, Król D. The role and importance of helmet use in the prevention of traumatic brain injury–a literature review. Polish Journal of Sports Medicine. 2023;39(4):159-69.

177. Peden AE, Cullen P, Bhandari B, Testa L, Wang A, Ma T, et al. A systematic review of the evidence for effectiveness of interventions to address transport and other unintentional injuries among adolescents. Journal of safety research. 2023;85:321-38.

178. Sun W, Abdullah LN, binti Khalid F, binti Sulaiman PS. Intelligent Analysis of Vehicle Accidents to Detect Road Safety: A Systematic Literature Review. 2023;13(11).

179. Wu F, Zhang Z, Han Z. How do cognitive interventions impact driver aggressiveness in China?—a driving simulator study. Journal of Advanced Transportation. 2023;2023(1):7300548.

180. Olsson N, Lidestam B, Thorslund B. Effect of train-driving simulator practice in the European rail traffic management system: an experimental study. Transportation research record. 2023;2677(5):694-706.

181. Huang Y, Chen F, Song M, Pan X, You K. Effect evaluation of traffic guidance in urban underground road diverging and merging areas: A simulator study. Accident Analysis & Prevention. 2023;186:107036.

182. De Vos B, Cuenen A, Ross V, Dirix H, Brijs K, Brijs T. The effectiveness of an intelligent speed assistance system with real-time speeding interventions for truck drivers: A Belgian simulator study. Sustainability. 2023;15(6):5226.

183. Amini RE, Al Haddad C, Batabyal D, Gkena I, De Vos B, Cuenen A, et al. Driver distraction and in-vehicle interventions: A driving simulator study on visual attention and driving performance. Accident Analysis & Prevention. 2023;191:107195.

184. Zangi N, Srour-Zreik R, Ridel D, Chassidim H, Borowsky A. Driver distraction and its effects on partially automated driving performance: A driving simulator study among young-experienced drivers. Accident Analysis & Prevention. 2022;166:106565.

185. Roy A, Hossain M, Muromachi Y. A deep reinforcement learning-based intelligent intervention framework for real-time proactive road safety management. Accident Analysis & Prevention. 2022;165:106512.

186. Rahman S, Mohan M, editors. Reduction of Vehicular Emission at Urban Road Junctions Through Traffic Interventions. International Conference on Transportation Infrastructure Projects: Conception to Execution; 2022: Springer.

187. Pešić D, Pešić D, Trifunović A, Čičević S. Application of logistic regression model to assess the impact of smartwatch on improving road traffic safety: A driving simulator study. Mathematics. 2022;10(9):1403.

188. Özkan Ö. The effect of road safety advertisements on speeding behavior over time: A simulator study. 2022.

189. Li X, Guo Z, Li Y. Driver operational level identification of driving risk and graded time-based alarm under near-crash conditions: A driving simulator study. Accident Analysis & Prevention. 2022;166:106544.

190. Kwon J-H, Kim J, Kim S, Cho G-H. Pedestrians safety perception and crossing behaviors in narrow urban streets: An experimental study using immersive virtual reality technology. Accident Analysis & Prevention. 2022;174:106757.

191. Hang J, Yan X, Li X, Duan K, Yang J, Xue Q. An improved automated braking system for rear-end collisions: A study based on a driving simulator experiment. Journal of safety research. 2022;80:416-27.

192. Eichberger A, Kraut M, Koglbauer IV. Improved perception of motorcycles by simulator-based driving education. Sustainability. 2022;14(9):5283.

193. Zhao X, Chen Y, Li H, Ma J, Li J. A study of the compliance level of connected vehicle warning information in a fog warning system based on a driving simulation. Transportation research part F: traffic psychology and behaviour. 2021;76:215-37.

194. Weigl K, Schartmüller C, Wintersberger P, Steinhauser M, Riener A. The influence of experienced severe road traffic accidents on take-over reactions and non-driving-related tasks in an automated driving simulator study. Accident analysis & prevention. 2021;162:106408.

195. Tekeş B, Özkan T. The effect of implementation intention on speeding and acceleration overtime: A simulator study. Transportation research part F: traffic psychology and behaviour. 2021;83:323-32.

196. Mas Cuesta L, Baltruschat S, Cándido Ortiz A, Maldonado López A, Verdejo Lucas C, Catena Verdejo E, et al. Repeat Traffic Offenders Improve Their Performance in Risky Driving Situations and Have Fewer Accidents Following a Mindfulness-Based Intervention. 2021.

197. Dimech-Betancourt B, Ponsford JL, Charlton JL, Ross PE, Gooden JR, Stolwyk RJ. Investigating feasibility and preliminary efficacy of a simulator-based driving intervention for people with acquired brain injury: A randomised controlled pilot study. Clinical rehabilitation. 2021;35(9):1277-89.

198. Baltruschat S, Mas-Cuesta L, Cándido A, Maldonado A, Verdejo-Lucas C, Catena-Verdejo E, et al. Repeat traffic offenders improve their performance in risky driving situations and have fewer accidents following a mindfulness-based intervention. Frontiers in psychology. 2021;11:567278.

199. Purnomo H, Setiawan D, editors. Effect of listening to Quran recitation on workload and driving performance: A car simulator study. IOP Conference Series: Materials Science and Engineering; 2020: IOP Publishing.

200. Lu Q, Tettamanti T, Hörcher D, Varga I. The impact of autonomous vehicles on urban traffic network capacity: an experimental analysis by microscopic traffic simulation. Transportation Letters. 2020;12(8):540-9.

201. Ka E, Kim D-G, Hong J, Lee C. Implementing Surrogate Safety Measures in Driving Simulator and Evaluating the Safety Effects of Simulator‐Based Training on Risky Driving Behaviors. Journal of advanced transportation. 2020;2020(1):7525721.

202. Bhardwaj A, Lu Y, Pan S, Sarter N, Gillespie B, editors. The effects of driver coupling and automation impedance on emergency steering interventions. 2020 IEEE International Conference on Systems, Man, and Cybernetics (SMC); 2020: IEEE.

203. Zimasa T, Jamson S, Henson B. The influence of driver’s mood on car following and glance behaviour: Using cognitive load as an intervention. Transportation research part F: traffic psychology and behaviour. 2019;66:87-100.

204. Zhao X, Xu W, Ma J, Gao Y. The “PNE” driving simulator-based training model founded on the theory of planned behavior. Cognition, Technology & Work. 2019;21:287-300.

205. Uzondu CC. The influence of road safety culture on driver behaviour: a study of Nigerian drivers: University of Leeds; 2019.

206. Urlings JH, Roelofs E, Cuenen A, Brijs K, Brijs T, Jongen EM. Development of single-session driving simulator-based and computer-based training for at-risk older drivers. Educational Gerontology. 2019;45(4):283-96.

207. Reinolsmann N, Alhajyaseen W, Brijs T, Pirdavani A, Hussain Q, Brijs K. Investigating the impact of dynamic merge control strategies on driving behavior on rural and urban expressways–A driving simulator study. Transportation research part F: traffic psychology and behaviour. 2019;65:469-84.

208. Ono S, Sasaki H, Kumon H, Fuwamoto Y, Kondo S, Narumi T, et al. Improvement of driver active interventions during automated driving by displaying trajectory pointers—A driving simulator study. Traffic injury prevention. 2019;20(sup1):S152-S6.

209. Muttart JW, Dinakar S, Fisher DL, Garrison TM, Samuel S. Evaluation of a training intervention to improve novice drivers’ hazard mitigation when approaching left turn scenarios. Transportation research record. 2019;2673(12):474-84.

210. Krishnan A, Samuel S, Yamani Y, Romoser MR, Fisher DL. Effectiveness of a strategic hazard anticipation training intervention in high risk scenarios. Transportation research part F: traffic psychology and behaviour. 2019;67:43-56.

211. Kazemzadehazad S, Monajjem S, Larue GS, King MJ. Evaluating new treatments for improving driver performance on combined horizontal and crest vertical curves on two-lane rural roads: A driving simulator study. Transportation research part F: traffic psychology and behaviour. 2019;62:727-39.

212. Domenichini L, Branzi V, Smorti M. Influence of drivers’ psychological risk profiles on the effectiveness of traffic calming measures. Accident Analysis & Prevention. 2019;123:243-55.

213. Chen T, Sze N, Bai L. Safety of professional drivers in an ageing society–A driving simulator study. Transportation research part F: traffic psychology and behaviour. 2019;67:101-12.

214. Chen F, Peng H, Ma X, Liang J, Hao W, Pan X. Examining the safety of trucks under crosswind at bridge-tunnel section: A driving simulator study. Tunnelling and Underground Space Technology. 2019;92:103034.

215. Banerjee S, Jeihani M, Morris D, editors. Impact of Work Zone Signage on Driver Speeding Behavior: A Driving Simulator Study. Transportation Research Board 98th Annual Meeting; 2019.

216. Arkonac SE, Brumby DP, Smith T, Babu HVR, editors. In-car distractions and automated driving: a preliminary simulator study. Proceedings of the 11th International Conference on Automotive User Interfaces and Interactive Vehicular Applications: adjunct Proceedings; 2019.

217. Ābele L, Haustein S, Martinussen LM, Møller M. Improving drivers’ hazard perception in pedestrian-related situations based on a short simulator-based intervention. Transportation research part F: traffic psychology and behaviour. 2019;62:1-10.

218. Zhao X, Ding H, Lin Z, Ma J, Rong J. Effects of longitudinal speed reduction markings on left-turn direct connectors. Accident Analysis & Prevention. 2018;115:41-52.

219. Yang L, Li X, Guan W, Zhang HM, Fan L. Effect of traffic density on drivers’ lane change and overtaking maneuvers in freeway situation—A driving simulator–based study. Traffic injury prevention. 2018;19(6):594-600.

220. Yamani Y, Bıçaksız P, Palmer DB, Hatfield N, Samuel S. Evaluation of the effectiveness of a gaze-based training intervention on latent hazard anticipation skills for young drivers: A driving simulator study. Safety. 2018;4(2):18.

221. Winkler S, Kazazi J, Vollrath M. Practice makes better–Learning effects of driving with a multi-stage collision warning. Accident Analysis & Prevention. 2018;117:398-409.

222. Tejero P, Insa B, Roca J. Increasing the default interletter spacing of words can help drivers to read traffic signs at longer distances. Accident Analysis & Prevention. 2018;117:298-303.

223. Sawula E, Polgar J, Porter MM, Gagnon S, Weaver B, Nakagawa S, et al. The combined effects of on-road and simulator training with feedback on older drivers' on-road performance: Evidence from a randomized controlled trial. Traffic injury prevention. 2018;19(3):241-9.

224. Nowosielski RJ, Trick LM, Toxopeus R. Good distractions: Testing the effects of listening to an audiobook on driving performance in simple and complex road environments. Accident Analysis & Prevention. 2018;111:202-9.

225. Li G, Wang F, Otte D, Cai Z, Simms C. Have pedestrian subsystem tests improved passenger car front shape? Accident Analysis & Prevention. 2018;115:143-50.

226. Lee JY, Lee JD, Bärgman J, Lee J, Reimer B. How safe is tuning a radio?: using the radio tuning task as a benchmark for distracted driving. Accident Analysis & Prevention. 2018;110:29-37.

227. Heikoop DD, de Winter JC, van Arem B, Stanton NA. Effects of mental demands on situation awareness during platooning: A driving simulator study. Transportation research part F: traffic psychology and behaviour. 2018;58:193-209.

228. Hatfield J, Williamson A, Kehoe EJ, Lemon J, Arguel A, Prabhakharan P, et al. The effects of training impulse control on simulated driving. Accident Analysis & Prevention. 2018;119:1-15.

229. Hajiseyedjavadi F, Zhang T, Agrawal R, Knodler M, Fisher D, Samuel S. Effectiveness of visual warnings on young drivers hazard anticipation and hazard mitigation abilities. Accident Analysis & Prevention. 2018;116:41-52.

230. Farahmand B, Boroujerdian AM. Effect of road geometry on driver fatigue in monotonous environments: A simulator study. Transportation research part F: traffic psychology and behaviour. 2018;58:640-51.

231. Cafiso S, D’Agostino C, Kieć M, Bak R. Safety assessment of passing relief lanes using microsimulation-based conflicts analysis. Accident Analysis & Prevention. 2018;116:94-102.

232. Alvaro PK, Burnett NM, Kennedy GA, Min WYX, McMahon M, Barnes M, et al. Driver education: Enhancing knowledge of sleep, fatigue and risky behaviour to improve decision making in young drivers. Accident Analysis & Prevention. 2018;112:77-83.

233. Wan J, Wu C, Zhang Y, Houston RJ, Chen CW, Chanawangsa P. Drinking and driving behavior at stop signs and red lights. Accident Analysis & Prevention. 2017;104:10-7.

234. Schwarz F, Fastenmeier W. Augmented reality warnings in vehicles: Effects of modality and specificity on effectiveness. Accident Analysis & Prevention. 2017;101:55-66.

235. Sander U. Opportunities and limitations for intersection collision intervention—A study of real world ‘left turn across path’accidents. Accident Analysis & Prevention. 2017;99:342-55.

236. Rahman MM, Strawderman L, Garrison T, Eakin D, Williams CC. Work zone sign design for increased driver compliance and worker safety. Accident Analysis & Prevention. 2017;106:67-75.

237. Rahman MM, Lesch MF, Horrey WJ, Strawderman L. Assessing the utility of TAM, TPB, and UTAUT for advanced driver assistance systems. Accident Analysis & Prevention. 2017;108:361-73.

238. Naujoks F, Purucker C, Wiedemann K, Neukum A, Wolter S, Steiger R. Driving performance at lateral system limits during partially automated driving. Accident Analysis & Prevention. 2017;108:147-62.

239. Mole CD, Wilkie RM. Looking forward to safer HGVs: The impact of mirrors on driver reaction times. Accident Analysis & Prevention. 2017;107:173-85.

240. Molan AM, Hummer JE. Safety analysis of the new synchronized and milwaukee B interchanges in comparison to existing designs. Accident Analysis & Prevention. 2017;109:29-35.

241. Mecheri S, Rosey F, Lobjois R. The effects of lane width, shoulder width, and road cross-sectional reallocation on drivers’ behavioral adaptations. Accident Analysis & Prevention. 2017;104:65-73.

242. Maillot P, Dommes A, Dang N-T, Vienne F. Training the elderly in pedestrian safety: Transfer effect between two virtual reality simulation devices. Accident Analysis & Prevention. 2017;99:161-70.

243. Li Y, Wang H, Wang W, Xing L, Liu S, Wei X. Evaluation of the impacts of cooperative adaptive cruise control on reducing rear-end collision risks on freeways. Accident Analysis & Prevention. 2017;98:87-95.

244. Li Y, Li Z, Wang H, Wang W, Xing L. Evaluating the safety impact of adaptive cruise control in traffic oscillations on freeways. Accident Analysis & Prevention. 2017;104:137-45.

245. Li G, Yang J, Simms C. Safer passenger car front shapes for pedestrians: A computational approach to reduce overall pedestrian injury risk in realistic impact scenarios. Accident Analysis & Prevention. 2017;100:97-110.

246. Jeong E, Oh C. Evaluating the effectiveness of active vehicle safety systems. Accident Analysis & Prevention. 2017;100:85-96.

247. Cœugnet S, Dommes A, Panëels S, Chevalier A, Vienne F, Dang N-T, et al. A vibrotactile wristband to help older pedestrians make safer street-crossing decisions. Accident Analysis & Prevention. 2017;109:1-9.

248. Classen S, Winter S, Monahan M, Yarney A, Link Lutz A, Platek K, et al. Driving intervention for returning combat veterans: Interim analysis of a randomized controlled trial. OTJR: occupation, participation and health. 2017;37(2):62-71.

249. Choudhary P, Velaga NR. Mobile phone use during driving: Effects on speed and effectiveness of driver compensatory behaviour. Accident Analysis & Prevention. 2017;106:370-8.

250. Chai C, Wong YD, Wang X. Safety evaluation of driver cognitive failures and driving errors on right-turn filtering movement at signalized road intersections based on Fuzzy Cellular Automata (FCA) model. Accident Analysis & Prevention. 2017;104:156-64.

251. Centofanti SA, Dorrian J, Hilditch CJ, Banks S. Do night naps impact driving performance and daytime recovery sleep? Accident Analysis & Prevention. 2017;99:416-21.

252. Zafian TM, Samuel S, Coppola J, O’Neill EG, Romoser MR, Fisher DL, editors. On-road effectiveness of a tablet-based teen driver training intervention. Proceedings of the Human Factors and Ergonomics Society Annual Meeting; 2016: SAGE Publications Sage CA: Los Angeles, CA.

253. Papadakaki M, Tzamalouka G, Gnardellis C, Lajunen TJ, Chliaoutakis J. Driving performance while using a mobile phone: A simulation study of Greek professional drivers. Transportation research part F: traffic psychology and behaviour. 2016;38:164-70.

254. Naujoks F, Kiesel A, Neukum A. Cooperative warning systems: The impact of false and unnecessary alarms on drivers’ compliance. Accident Analysis & Prevention. 2016;97:162-75.

255. Hamid M, Samuel S, Borowsky A, Horrey WJ, Fisher DL. Evaluation of training interventions to mitigate effects of fatigue and sleepiness on driving performance. Transportation Research Record. 2016;2584(1):30-8.

256. Freydier C, Berthelon C, Bastien-Toniazzo M. Does early training improve driving skills of young novice French drivers? Accident Analysis & Prevention. 2016;96:228-36.

257. Fahlstedt M, Halldin P, Kleiven S. The protective effect of a helmet in three bicycle accidents—A finite element study. Accident Analysis & Prevention. 2016;91:135-43.

258. Devos H, Morgan JC, Onyeamaechi A, Bogle CA, Holton K, Kruse J, et al. Use of a driving simulator to improve on‐road driving performance and cognition in persons with P arkinson's disease: A pilot study. Australian occupational therapy journal. 2016;63(6):408-14.

259. Ariën C. The effects in distance and time of traffic calming measures near road transitions and discontinuities by means of driving simulator research. 2016.

260. Zhao X, Wu Y, Rong J, Ma J. The effect of chevron alignment signs on driver performance on horizontal curves with different roadway geometries. Accident Analysis & Prevention. 2015;75:226-35.

261. Zhao X, Li J, Ding H, Zhang G, Rong J. A generic approach for examining the effectiveness of traffic control devices in school zones. Accident Analysis & Prevention. 2015;82:134-42.

262. Wang X, Wang T, Tarko A, Tremont PJ. The influence of combined alignments on lateral acceleration on mountainous freeways: A driving simulator study. Accident Analysis & Prevention. 2015;76:110-7.

263. Marciano H, Norman J. Overt vs. covert speed cameras in combination with delayed vs. immediate feedback to the offender. Accident Analysis & Prevention. 2015;79:231-40.

264. Larue GS, Kim I, Rakotonirainy A, Haworth NL, Ferreira L. Driver’s behavioural changes with new intelligent transport system interventions at railway level crossings—A driving simulator study. Accident Analysis & Prevention. 2015;81:74-85.

265. Krasnova O, Molesworth B, Williamson A, editors. The effect of cognitive-based training interventions on driver speed management behavior: A driving simulator study. Proceedings of the human factors and ergonomics society annual meeting; 2015: SAGE Publications Sage CA: Los Angeles, CA.

266. Dijksterhuis C, Lewis-Evans B, Jelijs B, de Waard D, Brookhuis K, Tucha O. The impact of immediate or delayed feedback on driving behaviour in a simulated Pay-As-You-Drive system. Accident Analysis & Prevention. 2015;75:93-104.

267. Crocetta G, Piantini S, Pierini M, Simms C. The influence of vehicle front-end design on pedestrian ground impact. Accident Analysis & Prevention. 2015;79:56-69.

268. Zhang X, Zhao X, Du H, Ma J, Rong J. Effect of different breath alcohol concentrations on driving performance in horizontal curves. Accident Analysis & Prevention. 2014;72:401-10.

269. Saurin TA, Wachs P, Righi AW, Henriqson E. The design of scenario-based training from the resilience engineering perspective: A study with grid electricians. Accident Analysis & Prevention. 2014;68:30-41.

270. Sager B, Yanko MR, Spalek TM, Froc DJ, Bernstein DM, Dastur FN. Motorcyclist's lane position as a factor in right-of-way violation collisions: A driving simulator study. Accident Analysis & Prevention. 2014;72:325-9.

271. Rosenbloom T, Eldror E. Effectiveness evaluation of simulative workshops for newly licensed drivers. Accident Analysis & Prevention. 2014;63:30-6.

272. Nygårdhs S, Lundkvist S-O, Andersson J, Dahlbäck N. The effect of different delineator post configurations on driver speed in night-time traffic: A driving simulator study. Accident Analysis & Prevention. 2014;72:341-50.

273. Kunc R, Omerović S, Ambrož M, Prebil I. Comparative study of European tunnel emergency-stop-area-wall protection measures. Accident Analysis & Prevention. 2014;63:9-21.

274. Casutt G, Theill N, Martin M, Keller M, Jäncke L. The drive-wise project: driving simulator training increases real driving performance in healthy older drivers. Frontiers in aging neuroscience. 2014;6:85.

275. Zhao G, Wu C. Effectiveness and acceptance of the intelligent speeding prediction system (ISPS). Accident Analysis & Prevention. 2013;52:19-28.

276. Merat N, Jamson AH. The effect of three low-cost engineering treatments on driver fatigue: A driving simulator study. Accident Analysis & Prevention. 2013;50:8-15.

277. Mennemeyer ST, Owsley C, McGwin Jr G. Reducing older driver motor vehicle collisions via earlier cataract surgery. Accident Analysis & Prevention. 2013;61:203-11.

278. Kim I, Larue G, Ferreira L, Tavassoli Hojati A, Rakotonirainy A, editors. Evaluating ITS interventions at railway level crossings using a driving simulator. Australasian Transport Research Forum 2013 Proceedings; 2013: Australasian Transport Research Forum.

279. Hughes GM, Rudin-Brown CM, Young KL. A simulator study of the effects of singing on driving performance. Accident Analysis & Prevention. 2013;50:787-92.

280. Habtemichael FG, de Picado-Santos L. The impact of high-risk drivers and benefits of limiting their driving degree of freedom. Accident Analysis & Prevention. 2013;60:305-15.

281. Guo Z, Wan H, Zhao Y, Wang H, Li Z. Driving simulation study on speed-change lanes of the multi-lane freeway interchange. Procedia-Social and Behavioral Sciences. 2013;96:60-9.

282. Ghajari M, Peldschus S, Galvanetto U, Iannucci L. Effects of the presence of the body in helmet oblique impacts. Accident Analysis & Prevention. 2013;50:263-71.

283. Cale MH, Gellert A, Katz N, Sommer W. Can minor changes in the environment lower accident risk at level crossings? Results from a driving simulator-based paradigm. Journal of Transportation Safety & Security. 2013;5(4):344-60.

284. Ariën C, Jongen EM, Brijs K, Brijs T, Daniels S, Wets G. A simulator study on the impact of traffic calming measures in urban areas on driving behavior and workload. Accident Analysis & Prevention. 2013;61:43-53.

285. Ünal AB, Steg L, Epstude K. The influence of music on mental effort and driving performance. Accident Analysis & Prevention. 2012;48:271-8.

286. Pascale A, Nicoli M, Deflorio F, Dalla Chiara B, Spagnolini U. Wireless sensor networks for traffic management and road safety. IET Intelligent Transport Systems. 2012;6(1):67-77.

287. Kircher K, Ahlstrom C. The impact of tunnel design and lighting on the performance of attentive and visually distracted drivers. Accident Analysis & Prevention. 2012;47:153-61.

288. Huth V, Biral F, Martín Ó, Lot R. Comparison of two warning concepts of an intelligent Curve Warning system for motorcyclists in a simulator study. Accident Analysis & Prevention. 2012;44(1):118-25.

289. Edquist J, Rudin-Brown CM, Lenné MG. The effects of on-street parking and road environment visual complexity on travel speed and reaction time. Accident Analysis & Prevention. 2012;45:759-65.

290. Dommes A, Cavallo V. Can simulator-based training improve street-crossing safety for elderly pedestrians? Transportation research part F: traffic psychology and behaviour. 2012;15(2):206-18.

291. Carpentier A. Training hazard perception of young novice drivers-a driving simulator study. 2012.

292. Auberlet J-M, Rosey F, Anceaux F, Aubin S, Briand P, Pacaux M-P, et al. The impact of perceptual treatments on driver's behavior: From driving simulator studies to field tests—First results. Accident Analysis & Prevention. 2012;45:91-8.

293. Schmidt EA, Schrauf M, Simon M, Buchner A, Kincses WE. The short-term effect of verbally assessing drivers’ state on vigilance indices during monotonous daytime driving. Transportation research part F: traffic psychology and behaviour. 2011;14(3):251-60.

294. Schleicher S, Gelau C. The influence of Cruise Control and Adaptive Cruise Control on driving behaviour–A driving simulator study. Accident Analysis & Prevention. 2011;43(3):1134-9.

295. Kapoor T, Altenhof W, Snowdon A, Howard A, Rasico J, Zhu F, et al. A numerical investigation into the effect of CRS misuse on the injury potential of children in frontal and side impact crashes. Accident Analysis & Prevention. 2011;43(4):1438-50.

296. Ben-Bassat T, Shinar D. Effect of shoulder width, guardrail and roadway geometry on driver perception and behavior. Accident Analysis & Prevention. 2011;43(6):2142-52.

297. Bella F, Russo R. A collision warning system for rear-end collision: a driving simulator study. Procedia-social and behavioral sciences. 2011;20:676-86.

298. Atchley P, Chan M. Potential benefits and costs of concurrent task engagement to maintain vigilance: A driving simulator investigation. Human factors. 2011;53(1):3-12.

299. Wang Y, Zhang W, Salvendy G. Effects of a simulation-based training intervention on novice drivers' hazard handling performance. Traffic injury prevention. 2010;11(1):16-24.

300. Inkeri H. Fatigue while driving in a car simulator: Effects on vigilance performance and autonomic skin conductance. 2010.

301. Fullerton M, Leonhardt A, Assenmacher S, Baur M, Busch F, Beltrán C, et al. Simulation study on improving traffic safety and traffic flow in the vicinity of a motorway accident through vehicle-to-vehicle communication. 2010.

302. Daniels S, Vanrie J, Dreesen A, Brijs T. Additional road markings as an indication of speed limits: results of a field experiment and a driving simulator study. Accident Analysis & Prevention. 2010;42(3):953-60.

303. Crundall D, Andrews B, Van Loon E, Chapman P. Commentary training improves responsiveness to hazards in a driving simulator. Accident Analysis & Prevention. 2010;42(6):2117-24.

304. Schmidt EA, Schrauf M, Simon M, Fritzsche M, Buchner A, Kincses WE. Drivers’ misjudgement of vigilance state during prolonged monotonous daytime driving. Accident Analysis & Prevention. 2009;41(5):1087-93.

305. Mackenzie J, Anderson R. The potential effects of electronic stability control interventions on rural road crashes in Australia: simulation of real world crashes. Austroads; 2009.

306. Jamson AH, Merat N, editors. Can low cost road engineering measures combat driver fatigue? A driving simulator investigation. Driving Assessment Conference; 2009: University of Iowa.

307. Isler RB, Starkey NJ, Williamson AR. Video-based road commentary training improves hazard perception of young drivers in a dual task. Accident Analysis & Prevention. 2009;41(3):445-52.

308. Devos H, Akinwuntan AE, Nieuwboer A, Tant M, Truijen S, De Wit L, et al. Comparison of the effect of two driving retraining programs on on-road performance after stroke. Neurorehabilitation and neural repair. 2009;23(7):699-705.

309. Vrignon J, Rakotonirainy A, Gruyer D, Saint Pierre G. Impact of subjective factors on driver vigilance: a driving simulator study. Driver Behaviour & Training. 2008.

310. Ting P-H, Hwang J-R, Doong J-L, Jeng M-C. Driver fatigue and highway driving: A simulator study. Physiology & behavior. 2008;94(3):448-53.

311. Pizza F, Contardi S, Ferlisi M, Mondini S, Cirignotta F. Daytime driving simulation performance and sleepiness in obstructive sleep apnoea patients. Accident Analysis & Prevention. 2008;40(2):602-9.

312. Freund B, Colgrove LA, Petrakos D, McLeod R. In my car the brake is on the right: Pedal errors among older drivers. Accident Analysis & Prevention. 2008;40(1):403-9.

313. de Lapparent M. Willingness to use safety belt and levels of injury in car accidents. Accident Analysis & Prevention. 2008;40(3):1023-32.

314. Chang S-H, Lin C-Y, Fung C-P, Hwang J-R, Doong J-L. Driving performance assessment: Effects of traffic accident location and alarm content. Accident Analysis & Prevention. 2008;40(5):1637-43.

315. Wallis TS, Horswill MS. Using fuzzy signal detection theory to determine why experienced and trained drivers respond faster than novices in a hazard perception test. Accident Analysis & Prevention. 2007;39(6):1177-85.

316. Howard ME, Jackson ML, Kennedy GA, Swann P, Barnes M, Pierce RJ. The interactive effects of extended wakefulness and low-dose alcohol on simulated driving and vigilance. Sleep. 2007;30(10):1334-40.

317. Yuhara N, Tajima J. Multi-driver agent-based traffic simulation systems for evaluating the effects of advanced driver assistance systems on road traffic accidents. Cognition, Technology & Work. 2006;8:283-300.

318. Desai AV, Haque MA. Vigilance monitoring for operator safety: A simulation study on highway driving. Journal of safety research. 2006;37(2):139-47.

319. Orth M, Duchna H, Leidag M, Widdig W, Rasche K, Bauer T, et al. Driving simulator and neuropsychological testing in OSAS before and under CPAP therapy. European Respiratory Journal. 2005;26(5):898-903.

320. Liu Y, Ozguner O, Ekici E, editors. Performance evaluation of intersection warning system using a vehicle traffic and wireless simulator. IEEE Proceedings Intelligent Vehicles Symposium, 2005; 2005: IEEE.

321. Akinwuntan AE, De Weerdt W, Feys H, Pauwels J, Baten G, Arno P, et al. Effect of simulator training on driving after stroke: a randomized controlled trial. Neurology. 2005;65(6):843-50.

322. Turkington P, Sircar M, Saralaya D, Elliott M. Time course of changes in driving simulator performance with and without treatment in patients with sleep apnoea hypopnoea syndrome. Thorax. 2004;59(1):56-9.

323. Charlton SG. Perceptual and attentional effects on drivers’ speed selection at curves. Accident Analysis & Prevention. 2004;36(5):877-84.

324. Thiffault P, Bergeron J. Monotony of road environment and driver fatigue: a simulator study. Accident Analysis & Prevention. 2003;35(3):381-91.

325. Roenker DL, Cissell GM, Ball KK, Wadley VG, Edwards JD. Speed-of-processing and driving simulator training result in improved driving performance. Human factors. 2003;45(2):218-33.

326. Duma SM, Crandall JR, Rudd RW, Kent RW. Small female head and neck interaction with a deploying side airbag. Accident Analysis & Prevention. 2003;35(5):811-6.

327. Orth M, Leidag M, Kotterba S, Widdig W, De Zeeuw J, Walther J, et al. Estimation of accident risk in obstructive sleep apnea syndrome (OSAS) by driving simulation. Pneumologie (Stuttgart, Germany). 2002;56(1):13-8.

328. Macchi MM, Boulos Z, Ranney T, Simmons L, Campbell SS. Effects of an afternoon nap on nighttime alertness and performance in long-haul drivers. Accident Analysis & Prevention. 2002;34(6):825-34.

329. Coeckelbergh TR, Brouwer WH, Cornelissen FW, Van Wolffelaar P, Kooijman AC. The effect of visual field defects on driving performance: a driving simulator study. Archives of ophthalmology. 2002;120(11):1509-16.

330. Hack M, Choi S, Vijayapalan P, Davies R, Stradling J. Comparison of the effects of sleep deprivation, alcohol and obstructive sleep apnoea (OSA) on simulated steering performance. Respiratory medicine. 2001;95(7):594-601.

331. George CF. Reduction in motor vehicle collisions following treatment of sleep apnoea with nasal CPAP. Thorax. 2001;56(7):508-12.

332. Weiler JM, Bloomfield JR, Woodworth GG, Grant AR, Layton TA, Brown TL, et al. Effects of fexofenadine, diphenhydramine, and alcohol on driving performance: a randomized, placebo-controlled trial in the Iowa driving simulator. Annals of Internal Medicine. 2000;132(5):354-63.

333. Findley L, Smith C, Hooper J, Dineen M, Suratt PM. Treatment with nasal CPAP decreases automobile accidents in patients with sleep apnea. American journal of respiratory and critical care medicine. 2000;161(3):857-9.

334. Verwey WB, Zaidel DM. Preventing drowsiness accidents by an alertness maintenance device. Accident Analysis & Prevention. 1999;31(3):199-211.

335. Touran A, Brackstone MA, McDonald M. A collision model for safety evaluation of autonomous intelligent cruise control. Accident Analysis & Prevention. 1999;31(5):567-78.

336. Jørgensen F, Wentzel-Larsen T. Optimal use of warning signs in traffic. Accident Analysis & Prevention. 1999;31(6):729-38.

337. Awane T. Integrating simulators in motorcycle safety education. IATSS research. 1999;23(HS-042 882).

338. McKnight AS, McKnight AJ, Tippetts AS. The effect of lane line width and contrast upon lanekeeping. Accident Analysis & Prevention. 1998;30(5):617-24.

339. Onken R, Feraric J. Adaptation to the driver as part of a driver monitoring and warning system. Accident Analysis & Prevention. 1997;29(4):507-13.

340. Mohan D, Kajzer J, Bawa-Bhalla K, Chawla A. Impact modelling studies for a threewheeled scooter taxi. Accident Analysis & Prevention. 1997;29(2):161-70.

341. Krieger J, Meslier N, Lebrun T, Levy P, Phillip-Joet F, Sailly J-C, et al. Accidents in obstructive sleep apnea patients treated with nasal continuous positive airway pressure: a prospective study. Chest. 1997;112(6):1561-6.

342. Gitelman V, Hakkert A-S. The evaluation of road-rail crossing safety with limited accident statistics. Accident Analysis & Prevention. 1997;29(2):171-9.

343. Chira-Chavala T, Yoo S. Potential safety benefits of intelligent cruise control systems. Accident Analysis & Prevention. 1994;26(2):135-46.

344. McKnight AJ, McKnight AS. The effect of cellular phone use upon driver attention. Accident Analysis & Prevention. 1993;25(3):259-65.

345. Suratt P, Findley L. Effect of nasal CPAP treatment on automobile driving simulator performance and on self-reported automobile accidents in subjects with sleep apnea. Am Rev Respir Dis. 1992;145(A169).

346. Alpert M, Ancoli-Israel S, Kripke D, Mason W, Espiritu R. Sleep-disordered breathing and car accidents in the elderly. Sleep Research. 1992;167.

347. Nelson T, Nilsson T. Comparing headphone and speaker effects on simulated driving: Accident Analysis and Prevention, 1990, 22.6, 523–529, 17 refs. Applied Ergonomics. 1991;22(6):421.

348. Lövsund P, Hedin A, Törnros J. Effects on driving performance of visual field defects: a driving simulator study. Accident Analysis & Prevention. 1991;23(4):331-42.

349. Haraldsson P-O, Carenfelt C, Laurell H, Tornros J. Driving vigilance simulator test. Acta oto-laryngologica. 1990;110(1-2):136-40.

350. Gawron VJ, Ranney TA. The effects of spot treatments on performance in a driving simulator under sober and alcohol-dosed conditions. Accident Analysis & Prevention. 1990;22(3):263-79.

351. Renaud L, Suissa S. Evaluation of the efficacy of simulation games in traffic safety education of kindergarten children. American Journal of Public Health. 1989;79(3):307-9.

352. Young DS, Lee DN. Training children in road crossing skills using a roadside simulation. Accident Analysis & Prevention. 1987;19(5):327-41.

353. Dingus TA, Hardee HL, Wierwille WW. Development of models for on-board detection of driver impairment. Accident Analysis & Prevention. 1987;19(4):271-83.

354. Veney JE, Kaiser DL. Development of a computer simulation of highway accident prevention and treatment. Accident Analysis & Prevention. 1976;8(4):279-91.

355. Shahdah UE, Na S, Hu JW. Assessing the Impact of Increasing Tractor-Trailer Speed Limit on the Safety and Mobility of Three-Lane Highways in Egypt. Applied Sciences. 2022;12(24):12702.

356. Liu B, Mehrara Molan A, Pande A, Howard J, Alexander S, Luo Z. Microscopic Traffic Simulation as a Decision support system for road diet and tactical urbanism strategies. Sustainability. 2021;13(14):8076.

357. Liu B, Shams A, Howard J, Alexander S, Hughes A, Pande A. Assessing Complete Street Strategies Using Microscopic Traffic Simulation Models. Mineta Transportation Institute, 2020.

358. Al-Ahmadi HM, Jamal A, Reza I, Assi KJ, Ahmed SA. Using microscopic simulation-based analysis to model driving behavior: a case study of Khobar-Dammam in Saudi Arabia. Sustainability. 2019;11(11):3018.

359. Domenichini L, Branzi V, Meocci M. Virtual testing of speed reduction schemes on urban collector roads. Accident Analysis & Prevention. 2018;110:38-51.

360. Helmer T, Wang L, Kompass K, Kates R, editors. Safety performance assessment of assisted and automated driving by virtual experiments: Stochastic microscopic traffic simulation as knowledge synthesis. 2015 IEEE 18th International Conference on Intelligent Transportation Systems; 2015: IEEE.

361. POORJAFARI V, YUE WL. Investigating the Impacts of Fixed-Time Ramp Metering through Microsimulation. Journal of the Eastern Asia Society for Transportation Studies. 2013;10:1824-35.

362. Isler RB, Starkey NJ, editors. Driver education and training as evidence-based road safety interventions. Wellington, NZ: Australasian Road Safety Research Policy Education Conference; 2012.

363. McDougall A, Brown J, Beck B, Bilston LE, editors. The effect of varied seat belt anchorage locations on booster seat sash guide effectiveness. 22nd International Technical Conference on the Enhanced Safety of Vehicles (ESV), Washington, DC; 2011.

364. Mahmud F. Using Micro-Simulation to Evaluate Transit Signal Priority for LRT Operations in A Downtown Environment: Old Dominion University; 2010.

365. Jamson S, Lai F, Jamson H. Driving simulators for robust comparisons: A case study evaluating road safety engineering treatments. Accident Analysis & Prevention. 2010;42(3):961-71.

366. Al Momani M. A Comparison of Traffic Flow Performance of Roundabouts and Signalized Intersections Using MITSIMLab: Master’s Thesis, Near East University, Graduate School of Applied Sciences …; 2009.

367. Viti F, Hoogendoorn SP, van Zuylen HJ, Wilmink IR, van Arem B, editors. Speed and acceleration distributions at a traffic signal analyzed from microscopic real and simulated data. 2008 11th international ieee conference on intelligent transportation systems; 2008: IEEE.

368. Abdel-Aty M, Pande A, Lee C, Gayah V, Santos CD. Crash risk assessment using intelligent transportation systems data and real-time intervention strategies to improve safety on freeways. Journal of Intelligent Transportation Systems. 2007;11(3):107-20.

369. Abdel-Aty M, Dhindsa A. Evaluation of ITS alternatives targeting crash mitigation on freeways using micro-simulation. Applications of Advanced Technology in Transportation2006. p. 485-91.

370. Dhindsa A. Evaluating ramp metering and variable speed limits to reduce crash potential on congested freeways using micro-simulation. 2005.

371. Davol AP. Modeling of traffic signal control and transit signal priority strategies in a microscopic simulation laboratory: Massachusetts institute of technology; 2001.

372. McGill SM, Callaghan JP. Impact forces following the unexpected removal of a chair while sitting. Accident Analysis & Prevention. 1999;31(1-2):85-9.

373. Racicot BM, Wogalter MS. Effects of a video warning sign and social modeling on behavioral compliance. Accident Analysis & Prevention. 1995;27(1):57-64.

374. Mahalel D, Zaidel D, Klein T. Driver's decision process on termination of the green light. Accident Analysis & Prevention. 1985;17(5):373-80.

375. Schmidt-Clausen H-J. The Visibility Distance of a Car-driver in Driving Situation1982.

376. Williams AF, Wells JK, Foss RD. The North Carolina Governor's Highway Safety Initiative: Initial Results from" Booze it and Lose It": Insurance Institute for Highway Safety; 1995.

377. Li H, Zhang Q. Traffic Organization Optimization of Wuluo Road Intersections in Wuhan. CICTP 2012: Multimodal Transportation Systems—Convenient, Safe, Cost-Effective, Efficient2012. p. 592-8.

378. Tay R. Do speed cameras improve road safety? Traffic and Transportation Studies (2000)2000. p. 44-51.

379. Agustsson L, editor Danish experiences with speed zones/variable speed limits. International Conference: Traffic Safety on Three ContinentsPTRC Education and Research Services Limited; 2001.

380. Agustsson L, Berggrein B, editors. Increased safety for cyclists in the town of Randers. Velo City'97: redescubrir la bicicleta-estrategias para una nueva movilidad (Barcelona, 15-19 septiembre 1997); 1997.

381. Arlow A, Duffy C, McDermid J, editors. Safety specification of the active traffic management control system for english motorways. The First Institution of Engineering and Technology International Conference on System Safety, 2006; 2006: IET.

382. Badiger A, Shapur C, Hongal R, Betageri S, Kumar N, editors. SysteMatic and automatic road traffic junction. 2016 International Conference on Electrical, Electronics, and Optimization Techniques (ICEEOT); 2016: IEEE.

383. Bailey J, editor Community alcohol action drink driving programmes in new zealand. Proceedings International Council on Alcohol, Drugs and Traffic Safety Conference; 1993: International Council on Alcohol, Drugs and Traffic Safety.

384. Balters S, Bernstein M, Paredes PE, editors. On-road stress analysis for in-car interventions during the commute. Extended Abstracts of the 2019 CHI Conference on Human Factors in Computing Systems; 2019.

385. Balters S, Landay JA, Paredes PE, editors. On-road guided slow breathing interventions for car commuters. Extended Abstracts of the 2019 CHI Conference on Human Factors in Computing Systems; 2019.

386. Beirness DJ, Foss RD, Mercer B, editors. Roadside breathtesting surveys to assess the impact of an enhanced DWI enforcement campaign in British Columbia. PROCEEDINGS OF THE 14TH INTERNATIONAL CONFERENCE OF ALCOHOL, DRUGS AND TRAFFIC-T'97, HELD ANNECY, FRANCE, 21-26 SEPTEMBER 1997, VOL 2; 1997.

387. Bouchard J, Dussault C, Simard R, Gendreau M, Lemire A, editors. The Quebec graduated licensing system for novice drivers: a two-year evaluation of the 1997 reform. Proceedings International Council on Alcohol, Drugs and Traffic Safety Conference; 2000: International Council on Alcohol, Drugs and Traffic Safety.

388. Brilon W, Blanke H, editors. Area-wide traffic calming measures and their effects on traffic safety in residential areas. LIVING AND MOVING IN CITIES PROCEEDINGS OF THE CONGRESS, PARIS, JANUARY 29-31, 1990; 1990.

389. Cameron MH, Strang P, Vulcan AP, editors. Evaluation of random breath testing in Victoria, Australia. Proceedings International Council on Alcohol, Drugs and Traffic Safety Conference; 1981: International Council on Alcohol, Drugs and Traffic Safety.

390. Castle S, Woods B, Davis J, Romero I, Flint S, Sewell C, editors. Outcome of a statewide sobriety checkpoint program. PROCEEDINGS OF THE 40TH ANNUAL CONFERENCE OF THE ASSOCIATION FOR THE ADVANCEMENT OF AUTOMOTIVE MEDICINE, VANCOUVER, BRITISH COLUMBIA, CANADA, OCTOBER 7-9, 1996; 1996.

391. Champness P, Sheehan M, Folkman L, editors. Time and distance halo effects of an overtly deployed mobile speed camera. 2005 Australasian Road Safety Research, Policing and Education Conference Proceedings; 2005: Research Coordination Advisory Group (RCAG) and the Australian Traffic ….

392. Cliff IDM, editor Effective Road Policing in Rural Areas: An Integrated Approach. ROAD SAFETY RESEARCH, POLICING AND EDUCATION CONFERENCE, 2003, SYDNEY, NEW SOUTH WALES, AUSTRALIA; 2003.

393. Crowther G, editor Engaging with motorcyclists: UK Police and the BikeSafe road safety programme. Australasian Road Safety Research Policing Education Conference, 2005, Wellington, New Zealand; 2005.

394. Daniels S, Nuyts E, Wets G, editors. Converting intersections to roundabouts: effects on accidents with bicyclists. Choice for Sustainable Development Pre-Proceedings of the 23rd PIARRC World Road CongressWorld Road Association (PIARC); 2007: Citeseer.

395. Davies R, editor Evaluation of" shared path rules" sign. SAFE CYCLING, CONFERENCE, 2000, BRISBANE, QUEENSLAND, AUSTRALIA; 2001.

396. Derby N, Hurst P, editors. The effects of random stopping in New Zealand. ALCOHOL, DRUGS AND TRAFFIC SAFETY T86; 1987.

397. Diamantopoulou K, Cameron MH, editors. The effects of random breath testing operations and drink-driving publicity on alcohol-related crashes in rural areas. Proceedings International Council on Alcohol, Drugs and Traffic Safety Conference; 2000: International Council on Alcohol, Drugs and Traffic Safety.

398. Gale A, Cairney P, Catchpole J, editors. Patterns of perceptual failures at intersections of arterial roads and local streets. Proceedings of the Fifth International Conference on Vision in Vehicles Elsevier, Glasgow; 1996.

399. Hamelynck P, editor Urban traffic safety strategies in the Netherlands. PROCEEDINGS OF ROAD SAFETY FOR CENTRAL AND EASTERN EUROPE A POLICY SEMINAR, HELD BUDAPEST, HUNGARY, OCTOBER 17-21, 1994; 1994.

400. Hart M, editor Effects on accidents, eliminating throughtraffic of cars in city areas. Proceedings (127–134) of Seminar on Short-term and Area-wide Evaluation of Safety Measures, Amsterdam, The Netherlands, April; 1982.

401. Hell T, Lob G, editors. Typical injury patterns of motorcyclists in different crash types: Effectiveness and improvements of countermeasures. Proceedings: Association for the Advancement of Automotive Medicine Annual Conference; 1993: Association for the Advancement of Automotive Medicine.

402. Homel R, editor Deterring the drinking driver: a theoretical model of the process. Proceedings, 10th International Conference on Alcohol, Drugs and Traffic Safety Amsterdam; 1986.

403. Homel R, McKay P, Henstridge J, editors. The impact on accidents of random breath testing in New South Wales: 1982-1992. Proceedings International Council on Alcohol, Drugs and Traffic Safety Conference; 1995: International Council on Alcohol, Drugs and Traffic Safety.

404. Jayatilleke AU, Marasinghe CA, Nakahara S, Nandasara S, Jayatilleke A, Jimba M, editors. Web based injury surveillance system (WBISS) for road traffic injuries. 7th IEEE International Conference on Computer and Information Technology (CIT 2007); 2007: IEEE.

405. Johannessen S, editor NEW STRATEGIES AND METHODS FOR SPEED MANAGEMENT IN NORWAY. SPEED MANAGEMENT STRATEGIES AND IMPLEMENTATION-PLANNING, EVALUATION, BEHAVIOURAL, LEGAL AND INSTITUTIONAL ISSUES-PROCEEDINGS AND ABSTRACTS OF 15TH ICTCT WORKSHOP HELD BRNO, CZECH REPUBLIC, OCTOBER 2002; 2002.

406. Kearns I, Goldsmith H, editors. The impact on traffic crashes of the introduction of random breath testing in New South Wales. Australian Road Research Board Conference Proc; 1984.

407. Khalid A, editor Towards a smarter solution for reducing road traffic accidents in dubai. 2019 International Conference on Computational Intelligence and Knowledge Economy (ICCIKE); 2019: IEEE.

408. Kubitzki J, editor Driving behaviour and personality in methadon patients. Proceedings International Council on Alcohol, Drugs and Traffic Safety Conference; 1997: International Council on Alcohol, Drugs and Traffic Safety.

409. Kumagai M, editor Assessment of the US bicycle reflector standard. Proceedings of the Seventh International Conference of European Consumer Safety Association (ECOSA) on Product Safety Research; 1999.

410. Lenné M, Dietze C, Rumbold G, Cvetkovski S, Redman J, Triggs T, editors. Comparison of the effects of methadone, LAAM, and buprenorphine on simulated driving performance. Proceedings of T2000–15th Conference on Alcohol, Drugs, and Traffic Safety, Held; 2000.

411. Li X, Mo H, Zhu F, editors. Interventions of traffic flow for intersection based on interval type-2 fuzzy sets. Proceedings 2014 International Conference on Informative and Cybernetics for Computational Social Systems (ICCSS); 2014: IEEE.

412. Macbeth AG, Underwood S, editors. Dunedin Cycle Lanes Safety Audit. New Zealand cycling conference; 2001.

413. Mara M, Davies R, Frith W, editors. Evaluation of the effect of compulsory breath testing and speed cameras in New Zealand. COMBINED 18TH ARRB TRANSPORT RESEARCH CONFERENCE AND TRANSIT NEW ZEALAND LAND TRANSPORT SYMPOSIUM, 2-6 SEPTEMBER 1996, CHRISTCHURCH, NEW ZEALAND PART 5; 1996.

414. Millot M, Méditerranée C, Hiron FB, editors. Does modern urban road layout improve road safety: which assessment? Proceedings of European Transport Conference, Leeuwenhorst, The Netherlands Association for European Transport and Contributors; 2008.

415. Motoki M, Yamazaki S, editors. A study on effective motorcycle rider education. Proceedings of the International Motorcycle Safety Conference; 1990.

416. Nafi NS, Khan JY, editors. A VANET based intelligent road traffic signalling system. Australasian Telecommunication Networks and Applications Conference (ATNAC) 2012; 2012: IEEE.

417. Panou M, Bekiaris E, editors. A new concept on the integration of driving simulators in driver training-the train-all approach. Proceedings of the Road Safety on Four Continents Conference; 2007: Conference Sponsor.

418. Pratama B, Christanto J, Hadyantama MT, Muis A, editors. Adaptive traffic lights through traffic density calculation on road pattern. 2018 international conference on applied science and technology (iCAST); 2018: IEEE.

419. Radalj T, editor Traffic safety effects of advanced warning flashing lights at intersections on high-speed roads, Perth metropolitan area. Road Safety Research, Policing and Education Conference; 2003.

420. Rahim A, Adnan M, Sulaiman N, Nazaruddin A, Zulqarnain F, editors. Safety implication of vehicles exceeding operating speed at different number of access in two lane carriageway. AIP Conference Proceedings; 2018: AIP Publishing.

421. Regan M, Deery H, Triggs T, editors. A technique for enhancing risk perception in novice car drivers (pp 51-55). the proceedings of the Road Safety Research, Policing and Education Conference Wellington, New Zealand: Land Transport Safety Authority and New Zealand Police; 1998.

422. Reveruzzi B, Buckley L, Dingli K, Chapman RL, Sheehan MC, editors. School-based first aid training and the implications for traffic injury prevention: a randomised controlled trial. International Traffic Medicine Association 23rd World Congress; 2013.

423. Rowden P, Watson B, Haworth N, editors. What can riders tell us about motorcycle rider training? A view from the other side of the fence. 2007 Australasian Road Safety Research, Policing and Education Conference; 2007: The Meeting Planners.

424. Scholtz J, Antonishek B, Young J, editors. Operator interventions in autonomous off-road driving: effects of terrain. 2004 IEEE international conference on systems, man and cybernetics (IEEE Cat No 04CH37583); 2004: IEEE.

425. Soderstrom CA, Scottino MA, Joyce JJ, Burch C, Ho SM, Kerns TJ, editors. Police referral of drivers to the Maryland Motor Vehicle Admininstration’s medical advisory board. Annals of Advances in Automotive Medicine/Annual Scientific Conference; 2009: Association for the Advancement of Automotive Medicine.

426. Sweedler BM, editor Strategies for dealing with the persistent drinking driver. Proceedings of the 13th International Conference on Alcohol, Drugs and Traffic Safety, Adelaide; 1995.

427. Thom DR, Hurt H, editors. Basilar skull fractures in fatal motorcycle crashes. Proceedings: Association for the Advancement of Automotive Medicine Annual Conference; 1993: Association for the Advancement of Automotive Medicine.

428. Thomson J, Mavrolefterou K, editors. Assessing the effectiveness of random breath testing. Australian Road Research Board Conference Proc; 1984.

429. Tollazzi T, editor Fifteen-year experience with roundabouts and measures of assuring traffic safe roundabouts in Slovenia. Proceedings of the Road Safety on Four Continents Conference; 2007: Conference Sponsor.

430. Trifunović A, Čičević S, Žunjić A, Mitrović S, Dragović M, editors. New pedagogical approaches in digital media-enhanced traffic safety training for children. Proceedings of the 18th Int Conf of the series Man & Working Environment 50 Years of Higher Education Science and Research of the Occupational Safety Engineering; 2018: University of Niš, Faculty of Occupational Safety.

431. Tuan VA, Shimizu T, editors. Towards development and evaluation of the motorcycle drivers re-education program in vietnam: Modeling of motorcycle driver’s undesired behaviors. 11th World Conference on Transport ResearchWorld Conference on Transport Research Society; 2007.

432. Vingilis ER, Chung L, Adlaf EM, editors. An evaluation of a prevention programme for drinking-driving called reduce impaired driving in Etobicoke (RIDE). Proceedings International Council on Alcohol, Drugs and Traffic Safety Conference; 1981: International Council on Alcohol, Drugs and Traffic Safety.

433. Xie S, Wu X, Lin J, editors. Traffic coordination optimization based on high density road network. Fifth International Conference on Traffic Engineering and Transportation System (ICTETS 2021); 2021: SPIE.

434. Amann G, Nogueira P. PW 1212 Changing mind-sets: what can be done in portugal, after policies implementation and huge reduction of road traffic injuries! : BMJ Publishing Group Ltd; 2018.

435. Kongdee T. PW 1919 Model development for road traffic injuries prevention and reduction by community participation, ban nai aoa community, moo 7, karaket sub district, chian yai, nakhon si thammarat, thailand. BMJ Publishing Group Ltd; 2018.

436. Maung NS. PW 2421 School-based road safety education in myanmar. Poster sessionRoad safety: BMJ Publishing Group Ltd; 2018.

437. Mojarro FR, Solorzano EH, Monreal MdlLA. PA 16-2-1073 Design and implementation of an initiative to reduce driving speed as a risk factor for road traffic injuries (RTI). BMJ Publishing Group Ltd; 2018.

438. Pirunsuntorn P, Thanalad W, Bamrung A. PW 2337 The study of the policy effects caused by the investigation report on road traffic injuries: a case study of the area in khon kaen province, thailand. BMJ Publishing Group Ltd; 2018.

439. Abd el-Shafy I, Savino J, Christopherson NA, Prince JM. Reduction of pediatric pedestrian hazardous road conditions in a school drop-off zone using video review. Journal of trauma and acute care surgery. 2017;83(5S):S227-S32.

440. Anderson A. Reliability in electromagnetic systems: the role of electrical contact resistance in maintaining automobile speed control system integrity. IET Conference Proceedings. 2007.

441. Boyes H, Luck A. A security-minded approach to vehicle automation, road infrastructure technology, and connectivity. 10th IET System Safety and Cyber-Security Conference

2015.

442. Brannolte U. Evaluation and comparison of traffic safety on high standard rural roads. VTI Rapport. 1990;(351A).

443. Carsten O. European research on ISA: Where are we now and what remains to be done. ICTCT, Nagoya. 2002.

444. Chapman R, Buckley L, Sheehan M, Shochet I. A process evaluation of a comprehensive school based injury prevention programme. Injury Prevention. 2010;16(Suppl 1):A91-A.

445. Cooper PJ. Estimating overinvolvement of seat belt nonwearers in crashes and the effect of lap/shoulder restraint use on different crash severity consequences. Accident Analysis & Prevention. 1994;26(2):263-75.

446. Decina LE, Staplin L. Retrospective evaluation of alternative vision screening criteria for older and younger drivers. Accident Analysis & Prevention. 1993;25(3):267-75.

447. Goodno M, McNeil N, Parks J, Dock S. Evaluation of innovative bicycle facilities in Washington, DC: Pennsylvania avenue median lanes and 15th street cycle track. Transportation research record. 2013;2387(1):139-48.

448. Helander CJ. Intervention strategies for accident-involved drivers: An experimental evaluation of current California policy and alternatives. Journal of Safety Research. 1984;15(1):23-40.

449. Hirsch L, Hosking J, van der Werf B, Mackie H, Hawley G, Wilson N, et al. Te Ara Mua–Future Streets: Road User Behaviour Outcomes. Journal of Transport & Health. 2019;14:100635.

450. Kearns I, Vazey B, Carseldine D, Arthurson R. An overview of the random breath testing trial in New South Wales. Alcohol, drugs and traffic safety. 1987:429-32.

451. Pendergrast RA, Ashworth CS, DuRant RH, Litaker M. Correlates of children's bicycle helmet use and short-term failure of school-level interventions. Pediatrics. 1992;90(3):354-8.

452. Pérez K, Santamariña-Rubio E. Are Barcelona’s Advanced Zones for Motorcycles Effective? Journal of Transport & Health. 2017;5:S47.

453. Poswayo A, Witte J, Guerrero A. PA 06-3-2810 Quantifying the effectiveness of a childhood road traffic injury prevention programme in dar es salaam. Injury Prevention. 2018;24(Suppl 2):A13.

454. Sung J, Mizenko K, Pierce R. P12 Law Enforcement Involvement and Traffic Safety Effectiveness: Findings from the National Highway Traffic Safety Administration׳ s (NHTSA)׳ s 2013 Countermeasures That Work. Journal of Transport & Health. 2015;2(2):S69-S70.

455. Tom Brimson B, Australia CEM, Anderson R. Fixed red light and speed cameras in Canberra: Evaluating a new digital technology. 2002.

456. Towliat M. Experiments regarding safety measures for pedestrian & cyclist in interactions with cars on main roads in built-up areas. Lund: Lund University. 2005.

457. Vincent E. A Critical Appraisal of Road Lighting and Accident Studies. CIVIL ENGINEERING WORKING PAPER. 1981;(Monograph).

458. Wegman F, Goldenbeld C. Speed management: enforcement and new technologies. 2006.

459. Werner J. DUTCH VARIABLE SPEED PILOT TEST EMPLOYS PHOTO SPEED ENFORCEMENT. Newsletter of the ITS Cooperative Deployment Network [online]. 2002.

460. Wolfe A. Evaluation of the special alcohol enforcement/education project in Oakland County, Michigan. Alcohol, Drugs, and Traffic Safety; edited by S Kaye and GW Meier NHTSA, Department of Transportation, Washington, DC. 1985:1575-96.

461. Zheng X, Fu X. Reflections on legislation about construction of integrated transportation system. 5th Advanced Forum on Transportation of China (AFTC 2009). 2009.

462. Ngoc AM, Minh CC, Nhu NT, Nishiuchi H, Huynh N. Influence of the human development index, motorcycle growth and policy intervention on road traffic fatalities–A case study of Vietnam. International journal of transportation science and technology. 2023;12(4):925-36.

463. Boniface S. Assessment of the Effectiveness of Interventions used in preventing Road Traffic Accidents in Tanzania: A case study of Kigoma Ujiji: The Open University of Tanzania; 2023.

464. Marcano M, Tango F, Sarabia J, Chiesa S, Pérez J, Díaz S. Can shared control improve overtaking performance? Combining human and automation strengths for a safer maneuver. Sensors. 2022;22(23):9093.

465. Cafiso S, Di Graziano A, Giuffrè T, Pappalardo G, Severino A. Managed lane as strategy for traffic flow and safety: A case study of Catania ring road. Sustainability. 2022;14(5):2915.

466. Macmillan A, Mackie H, Hosking J, Witten K, Smith M, Field A, et al. Controlled before-after intervention study of suburb-wide street changes to increase walking and cycling: Te Ara Mua-Future Streets study design. BMC public health. 2018;18:1-13.

467. Jeffreys I, Graves G, Roth M. Evaluation of eco-driving training for vehicle fuel use and emission reduction: A case study in Australia. Transportation Research Part D: Transport and Environment. 2018;60:85-91.

468. Barin EN, McLaughlin CM, Farag MW, Jensen AR, Upperman JS, Arbogast H. Heads up, phones down: A pedestrian safety intervention on distracted crosswalk behavior. Journal of community health. 2018;43:810-5.

469. Arbogast H, Patao M, Demeter N, Bachman S, Devietti E, Upperman JS, et al. The effectiveness of installing a speed hump in reducing motor vehicle accidents involving pedestrians under the age of 21. Journal of Transport & Health. 2018;8:30-4.

470. Newnam S, Oxley J. A program in safety management for the occupational driver: Conceptual development and implementation case study. Safety science. 2016;84:238-44.

471. Hammond J, Cherrett T, Waterson B. Making in‐class skills training more effective: The scope for interactive videos to complement the delivery of practical pedestrian training. British Journal of Educational Technology. 2015;46(6):1344-53.

472. Bachman SL, Arbogast H, Ruiz P, Farag M, Demeter NE, Upperman JS, et al. A school–hospital partnership increases knowledge of pedestrian and motor vehicle safety. 2015;40:1057-64.

473. Tyrone Adey B, Lethanh N, Hartmann A, Viti F. Evaluation of intervention strategies for a road link in the Netherlands. Built environment project and asset management. 2014;4(2):180-98.

474. Allen PA, Lien M-C, Ruthruff E, Voss A. Multitasking and aging: do older adults benefit from performing a highly practiced task? Experimental aging research. 2014;40(3):280-307.

475. Koestner AL. ThinkFirst for teens: Finding an injury-prevention approach for teenagers. Journal of Trauma Nursing| JTN. 2012;19(4):227-31.

476. Esperato A, Bishai D, Hyder AA. Projecting the health and economic impact of road safety initiatives: a case study of a multi-country project. Traffic injury prevention. 2012;13(sup1):82-9.

477. Dommes A, Cavallo V, Vienne F, Aillerie I. Age-related differences in street-crossing safety before and after training of older pedestrians. Accident Analysis & Prevention. 2012;44(1):42-7.

478. Wegman F, Weijermars W, editors. Ten years sustainable safety in the Netherlands; an assessment. TRB 90th Annual Meeting, Washington DC; 2011: Transportation Research Board (TRB).

479. Zehnder D, Meuli M, Landolt MA. Effectiveness of a single-session early psychological intervention for children after road traffic accidents: A randomised controlled trial. Child and adolescent psychiatry and mental health. 2010;4:1-10.

480. Philbrook JK, Franke-Wilson NA. The effectiveness of a peer lead smart driving campaign on high school students’ driving habits. Journal of Trauma and Acute Care Surgery. 2009;67(1):S67-S9.

481. Guanche-Garcell H, Suárez Enríquez T, Gutiérrez García F, Martínez Quesada C, Peña Sandoval R, Sánchez Villalobos J. Impact of a drink-driving detection program to prevent traffic accidents (Villa Clara Province, Cuba). Gaceta Sanitaria. 2008;22(4):344-7.

482. Forsyth AJ. Banning glassware from nightclubs in Glasgow (Scotland): observed impacts, compliance and patron's views. Alcohol & Alcoholism. 2008;43(1):111-7.

483. Fesperman CE, Evenson KR, Rodríguez DA, Salvesen D. A comparative case study on active transport to and from school. Preventing chronic disease. 2008;5(2).

484. Chianti Terri Allabaugh SM, Glenn Carlson, Kuanwong Watcharotone. Using Trauma Nurses Talk Tough Presentation With Pretest and Posttest Evaluation of Knowledge and Behavior Changes. Journal of trauma nursing. 2008;15(3):102-11.

485. Treno AJ, Gruenewald PJ, Lee JP, Remer LG. The Sacramento Neighborhood Alcohol Prevention Project: outcomes from a community prevention trial. Journal of studies on alcohol and drugs. 2007;68(2):197-207.

486. Mulvaney CA, Kendrick D, Watson MC, Coupland CA. Increasing child pedestrian and cyclist visibility: cluster randomised controlled trial. Journal of Epidemiology & Community Health. 2006;60(4):311-5.

487. König S. Evaluation of the effects of rebuilt bicycle paths at intersections on arterial streets in Lund: a case study. 2006.

488. Chen K-T, Chen C-T, Mardini S, Tsay P-K, Chen Y-R. Frontal sinus fractures: a treatment algorithm and assessment of outcomes based on 78 clinical cases. Plastic and Reconstructive Surgery. 2006;118(2):457-68.

489. Gunarta S, Kerr G. Speed impacts of mobile speed cameras in Christchurch. Road & Transport Research. 2005;14(2):16.

490. Cooper D, Atkins F, Gillen D. Measuring the impact of passenger restrictions on new teenage drivers. Accident Analysis & Prevention. 2005;37(1):19-23.

491. Tester JM, Rutherford GW, Wald Z, Rutherford MW. A matched case–control study evaluating the effectiveness of speed humps in reducing child pedestrian injuries. American journal of public health. 2004;94(4):646-50.

492. Sayer JR, Mefford ML. High visibility safety apparel and nighttime conspicuity of pedestrians in work zones. Journal of Safety Research. 2004;35(5):537-46.

493. Homel R, Carvolth R, Hauritz M, McIlwain G, Teague R. Making licensed venues safer for patrons: what environmental factors should be the focus of interventions? Drug and Alcohol review. 2004;23(1):19-29.

494. Hitosugi M, Shigeta A, Takatsu A, Yokoyama T, Tokudome S. Analysis of fatal injuries to motorcyclists by helmet type. The American journal of forensic medicine and pathology. 2004;25(2):125-8.

495. Rimiller J, Ivan J, Garrick N. Estimating benefits from specific highway safety improvements: Phase III: Safety benefits from left turn treatment. Connecticut Cooperative Highway Research Program, Project. 2003:97-1.

496. Maguire M, Nettleton H, Rix A, Raybould S. Reducing alcohol-related violence and disorder: an evaluation of the'TASC'project: Home Office London; 2003.

497. White M, Walker J. Update and overview of research on the effectiveness of the Victorian Transport Accident Commission's road safety television advertising campaigns. 2002.

498. Stradling S, Campbell M, editors. The Effects of Safety Cameras on Drivers. ROSPA 67th Road Safety Congress; 2002: Citeseer.

499. Licata M, Gillham K, Campbell E. Health promotion practices of restaurants and cafes in Australia: changes from 1997 to 2000 using an annual telemarketing intervention. Health Promotion International. 2002;17(3):255-62.

500. Ulmer RG, Ferguson SA, Williams AF, Preusser DF. Teenage crash reduction associated with delayed licensure in Connecticut. Journal of Safety Research. 2001;32(1):31-41.

501. Phillips S, Todman J. Pedestrian skills training for children with learning difficulties. International journal of rehabilitation research. 1999;22(3):237-8.

502. Boots K, Midford R. ‘Pick-a-Skipper': an evaluation of a designated driver program to prevent alcohol-related injury in a regional Australian city. Health Promotion International. 1999;14(4):337-45.

503. Cairney P. The conspicuity of bicycle lighting under on-road conditions. Vision in vehicles. 1998;6:185-90.

504. Weiss A, Freels S. The effects of aggressive policing: The Dayton traffic enforcement experiment. American Journal of Police. 1996;15(3):45-64.

505. Konrad CJ, Fieber TS, Schuepfer GK, Gerber HR. Are fractures of the base of the skull influenced by the mass of the protective helmet? A retrospective study in fatally injured motorcyclists. Journal of Trauma and Acute Care Surgery. 1996;41(5):854-8.

506. Ferguson SA, Leaf WA, Williams AF, Preusser DF. Differences in young driver crash involvement in states with varying licensure practices. Accident Analysis & Prevention. 1996;28(2):171-80.

507. Jones B. The effectiveness of provisional licensing in Oregon: an analysis of traffic safety benefits. Journal of Safety Research. 1994;25(1):33-46.

508. Benekohal RF, Resende PT, Zhao W. Temporal speed reduction effects of drone radar in work zones. Transportation Research Record. 1993;1409:32-41.

509. YOUNGMAN J, editor THE VICTORIAN MYTH: AN EXAMINATION OF THE EFFECTIVENESS OF DRINK DRIVING COUNTERMEASURES. ROAD TRAFFIC SAFETY SEMINAR: 14-16 SEPTEMBER; 1988.

510. Verschuur W, NOORDZIJ P. RANDOM BREATH TESTING ON A SMALL SCALE--ROAD USER BEHAVIOR. THEORY AND RESEARCH. PAPERS PRESENTED AT THE 2ND INTERNATIONAL CONFERENCE ON ROAD SAFETY HELD IN GRONINGEN, NETHERLANDS, AUGUST 1987. Publication of: VAN GORCUM & COMP BV. 1988.

511. Hagge RA, Marsh WC. An evaluation of the traffic safety impact of provisional licensing. California Department of Motor Vehicles, Sacramento, California. 1988.

512. Harvey AC, Durbin J. The effects of seat belt legislation on British road casualties: A case study in structural time series modelling. Journal of the Royal Statistical Society: Series A (General). 1986;149(3):187-210.

513. Beith BH, Sanders MS, Peay JM. Using retroreflective material to enhance the conspicuity of coal miners. Human Factors. 1982;24(6):727-35.

514. Blomberg RD, Leaf WA, Jacobs HH. Detection and recognition of pedestrians at night. Transportation Research Circular. 1981;229:17-21.

515. Vingilis E, Salutin L. A prevention programme for drinking driving. Accident Analysis & Prevention. 1980;12(4):267-74.

516. Hoffman G. The effectiveness of the helmet for the motorcyclist (machine under 50 cc), and the importance of alcoholemia in traffic accidents. Journal of traffic medicine. 1977;5(2):36-7.

517. Hazlett RD, Allen MJ. The ability to see a pedestrian at night: The effects of clothing, reflectorization and driver intoxication. Optometry and Vision Science. 1968;45(4):246-58.

518. Abraham B. Application of intervention analysis to a road fatality series in Ontario. Journal of Forecasting. 1987;6(3):211-9.

519. Adnan M, Ali MS, Qadir A, Sheeraz K, editors. Increasing effectiveness of road safety interventions-An operational model for developing countries. First International Forum of Traffic Safety; 2011.

520. Afukaar FK. Speed control in developing countries: issues, challenges and opportunities in reducing road traffic injuries. Injury control and safety promotion. 2003;10(1-2):77-81.

521. Al Turki YA. How can Saudi Arabia use the Decade of Action for Road Safety to catalyse road traffic injury prevention policy and interventions? International journal of injury control and safety promotion. 2014;21(4):397-402.

522. Andersson A-L, Dahlbäck L-O, Bunketorp O. Psychosocial aspects of road traffic trauma–benefits of an early intervention? Injury. 2005;36(8):917-26.

523. Andrade C, Telles B, Sercheli M, Kawano N, Soares R, Vicente A, et al. Road design intervention based on traffic accident dynamics: a forensic intelligence approach. WIT Transactions on the Built Environment. 2015;146:569-78.

524. Bakhtari Aghdam F, Sadeghi-Bazargani H, Azami-Aghdash S, Esmaeili A, Panahi H, Khazaee-Pool M, et al. Developing a national road traffic safety education program in Iran. BMC public health. 2020;20:1-13.

525. Banfield J. Innovative strategies to reduce traffic related injuries and deaths in youth. Journal of Local and Global Heath Science. 2015;2015(2):72.

526. Berhanu Y, Alemayehu E, Schröder D. Examining Car Accident Prediction Techniques and Road Traffic Congestion: A Comparative Analysis of Road Safety and Prevention of World Challenges in Low‐Income and High‐Income Countries. Journal of advanced transportation. 2023;2023(1):6643412.

527. Bishai D, Asiimwe B, Abbas S, Hyder AA, Bazeyo W. Cost-effectiveness of traffic enforcement: case study from Uganda. Injury Prevention. 2008;14(4):223-7.

528. Bjørnskau T, Elvik R. Can road traffic law enforcement permanently reduce the number of accidents? Accident Analysis & Prevention. 1992;24(5):507-20.

529. Bouraima MB, Kiptum CK, Ndiema KM, Qiu Y, Tanackov I. Prioritization road safety strategies towards zero road traffic injury using ordinal priority approach. Operational research in engineering sciences: theory and applications. 2022;5(2):206-21.

530. Buitelaar E, Van der Heijden R, Argiolu R. Managing traffic by privatization of road capacity: A property rights approach. Transport Reviews. 2007;27(6):699-713.

531. Carbonell EJ. Social intervention in traffic safety. Traffic Psychology Today: Springer; 2001. p. 219-39.

532. Cobiac LJ, Mizdrak A, Wilson N. Cost-effectiveness of raising alcohol excise taxes to reduce the injury burden of road traffic crashes. Injury prevention. 2019;25(5):421-7.

533. Ditsuwan V, Veerman JL, Bertram M, Vos T. Cost-effectiveness of interventions for reducing road traffic injuries related to driving under the influence of alcohol. Value in health. 2013;16(1):23-30.

534. Elvik R. Evaluations of road accident blackspot treatment: a case of the iron law of evaluation studies? Accident Analysis & Prevention. 1997;29(2):191-9.

535. Elvik R. Analytic choices in road safety evaluation: Exploring second-best approaches. Accident Analysis & Prevention. 2012;45:173-9.

536. Fanai S, Mohammadnezhad M. The perception of public transport drivers (PTDs) on preventing road traffic injury (RTIs) in Vanuatu: a qualitative study. International journal of qualitative studies on health and well-being. 2022;17(1):2047253.

537. Fanai S, Mohammadnezhad M, Salusalu M. Perception of law enforcement officers on preventing road traffic injury in Vanuatu: a qualitative study. Frontiers in public health. 2021;9:759654.

538. Faulks I, editor Addressing issues of driver distraction in traffic offender management. Australasian College of Road Safety Conference; 2012.

539. Feng Z, Zhan J, Ma C, Lei Y, Liu J, Zhang W, et al. Is cognitive intervention or forgiveness intervention more effective for the reduction of driving anger in Chinese bus drivers? Transportation research part F: traffic psychology and behaviour. 2018;55:101-13.

540. Fildes B, Keall M, Thomas P, Parkkari K, Pennisi L, Tingvall C. Evaluation of the benefits of vehicle safety technology: The MUNDS study. Accident Analysis & Prevention. 2013;55:274-81.

541. Fwa T, Chu L, Tan K. Rational procedure for determination of rut depth intervention level in network-level pavement management. Transportation Research Record. 2016;2589(1):59-67.

542. González EL, Vásquez RAD, Yacelga AL, Aldas MR, editors. Road Education and GIS Technology as keys to the reduction of traffic accidents. 2023 18th Iberian Conference on Information Systems and Technologies (CISTI); 2023: IEEE.

543. Gururaj G, editor Behavior and road safety: a multidimensional issue—implications for road safety programmes in developing countries. Transportation, Traffic Safety and Health—Human Behavior: Fourth International Conference, Tokyo, Japan, 1998; 2000: Springer.

544. Harris GT, Olukoga I. A cost benefit analysis of an enhanced seat belt enforcement program in South Africa. Injury prevention. 2005;11(2):102-5.

545. Huicho L, Adam T, Rosales E, Paca-Palao A, López L, Luna D, et al. Evaluation of interventions on road traffic injuries in Peru: a qualitative approach. BMC public health. 2012;12:1-13.

546. Ingirige B, Alshamsi MEIK. TRAFFIC ACCIDENTS PREVENTION INTERVENTIONS FOR ABU DHABI ROADS. Police Thought. 2020;29(114).

547. Jiang K, Wang Y, Feng Z, Cui J, Huang Z, Yu Z, et al. Research on intervention methods for children’s street-crossing behaviour: Application and expansion of the theory of “behaviour spectrums”. Accident Analysis & Prevention. 2021;152:105979.

548. Johnston JJ, Hendricks SA, Fike JM. Effectiveness of behavioral safety belt interventions. Accident Analysis & Prevention. 1994;26(3):315-23.

549. Jonah BA, Wilson RJ. Improving the effectiveness of drinking-driving enforcement through increased efficiency. Accident Analysis & Prevention. 1983;15(6):463-81.

550. Juillard C, Labinjo M, Kobusingye O, Hyder AA. Socioeconomic impact of road traffic injuries in West Africa: exploratory data from Nigeria. Injury prevention. 2010;16(6):389-92.

551. Kamarudin MKA, Abd Wahab N, Umar R, Saudi ASM, Saad MHM, Rosdi NRN, et al. Road traffic accident in Malaysia: trends, selected underlying, determinants and status intervention. Int J Eng Technol. 2018;7(4.34):112.

552. Kessi M, Sokoni D, Kitambi M, Katopola D, Fedjo G, Kileo N, et al. Reducing deaths and injury from road traffic crashes through multisectoral collaboration. The Pan African Medical Journal. 2023;45(Suppl 1).

553. Khorasani-Zavareh D, Mohammadi R, Khankeh HR, Laflamme L, Bikmoradi A, Haglund BJ. The requirements and challenges in preventing of road traffic injury in Iran. A qualitative study. BMC public health. 2009;9:1-9.

554. Kunkel E. Driver improvement courses for drinking-drivers reconsidered. Accident Analysis & Prevention. 1983;15(6):429-39.

555. Lewis I, Watson B, White KM. The Step approach to Message Design and Testing (SatMDT): A conceptual framework to guide the development and evaluation of persuasive health messages. Accident Analysis & Prevention. 2016;97:309-14.

556. Liban CB, Vingilis ER, Blefgen H. The Canadian drinking-driving countermeasure experience. Accident Analysis & Prevention. 1987;19(3):159-81.

557. Littleton S, Hughes D, Gopinath B, Robinson B, Poustie S, Smith P, et al. The health status of people claiming compensation for musculoskeletal injuries following road traffic crashes is not altered by an early intervention programme: a comparative study. Injury. 2014;45(9):1493-9.

558. Lukumay GG, Outwater AH, Mkoka DA, Ndile ML, Saveman B-I. Traffic police officers’ experience of post-crash care to road traffic injury victims: a qualitative study in Tanzania”. BMC emergency medicine. 2019;19:1-11.

559. M. Selveindran S, Samarutilake GD, Vera DS, Brayne C, Hill C, Kolias A, et al. Prevention of road traffic collisions and associated neurotrauma in Colombia: An exploratory qualitative study. Plos one. 2021;16(3):e0249004.

560. Mann RE, Vingilis ER, Leigh G, Anglin L, Blefgen H. School-based programmes for the prevention of drinking and driving: Issues and results. Accident Analysis & Prevention. 1986;18(4):325-37.

561. Mazaheri M, Rezai-Rad M, Pelarak F. Strategies to reduce road traffic injuries among motorcyclists in Dezful, Iran: stressing on legal and environmental factors. Journal of injury and violence research. 2022;14(1):89.

562. McKenna FP. The perceived legitimacy of intervention: A key feature for road safety. Improving traffic safety culture in the United States: The journey forward. 2007:165-75.

563. Mendivil J, García-Altés A, Pérez K, Marí-Dell'Olmo M, Tobías A. Speed cameras in an urban setting: a cost–benefit analysis. Injury prevention. 2012;18(2):75-80.

564. Michon JA. Traffic education for young pedestrians: An introduction. Accident Analysis & Prevention. 1981;13(3):163-7.

565. Mohajer B, Azmin M, Mohebi F, Ahmadi N, Farzadfar F. Low-Quality Domestic Automobiles Continue to Threaten Lives in Iran: Economic Instability as the Potential Contributor. Archives of Iranian Medicine (AIM). 2020;23(11).

566. MORGADO J, NEVES J, editors. AN INTEGRATED METHODOLOGY FOR PLANNING ROAD PAVEMENT MAINTENANCE AND REHABILITATION INTERVENTIONS WITHIN HIGH-TRAFFIC CONTEX. Proceedings of the 13th WCTR, World Conference on Transport Research; 2013.

567. Morrison DS, Thomson H, Petticrew M. Evaluation of the health effects of a neighbourhood traffic calming scheme. Journal of Epidemiology & Community Health. 2004;58(10):837-40.

568. Nakahara S, Ichikawa M, Wakai S. Magazine information on safety belt use for pregnant women and young children. Accident Analysis & Prevention. 2007;39(2):356-63.

569. Ogunwolu L, Sosimi A, Jagun O, Onyedikam C. Optimal routing for automated emergency vehicle response for incident intervention in a traffic network. Journal of Applied Sciences and Environmental Management. 2018;22(12):1941-6.

570. Osayomi T. Regional determinants of road traffic accidents in Nigeria: identifying risk areas in need of intervention. African geographical review. 2013;32(1):88-99.

571. Papakitsos EC, Korakidi G, Vamvakeros X, Papavasileiou C, Giannopoulos S, Mavrakis A. A systemic intervention of traffic education focused on road safety. International Journal of Education Humanities and Social Science. 2020;3(2):182-94.

572. Peltzer K, Mashego T. Perceptions of road traffic injury causes and interventions in the Limpopo Province, South Africa: implications for prevention. Acta Criminologica: African Journal of Criminology & Victimology. 2003;16(2):30-42.

573. Qiao Y, Dawson AR, Parry T, Flintsch GW. Evaluating the effects of climate change on road maintenance intervention strategies and Life-Cycle Costs. Transportation Research Part D: Transport and Environment. 2015;41:492-503.

574. Ralaidovy AH, Bachani AM, Lauer JA, Lai T, Chisholm D. Cost-effectiveness of strategies to prevent road traffic injuries in eastern sub-Saharan Africa and Southeast Asia: new results from WHO-CHOICE. Cost effectiveness and resource allocation. 2018;16:1-10.

575. Rosenfeld M, Seferiadis A, Gunnarsson R. Active involvement and intervention in patients exposed to whiplash trauma in automobile crashes reduces costs: a randomized, controlled clinical trial and health economic evaluation. Spine. 2006;31(16):1799-804.

576. Ross HL, Klette H, McCleary R. Liberalization and rationalization of drunk-driving laws in Scandinavia. Accident Analysis & Prevention. 1984;16(5-6):471-87.

577. Santos J, Ferreira A. Pavement design optimization considering costs and preventive interventions. Journal of Transportation Engineering. 2012;138(7):911-23.

578. Scuffham PA. Cost-effectiveness analyses for injury prevention initiatives in low-and middle-income countries. Injury prevention. 2008;14(4):217-9.

579. Sieber M, Siedersberger K-H, Siegel A, Färber B, editors. Automatic emergency steering with distracted drivers: Effects of intervention design. 2015 IEEE 18th international conference on intelligent transportation systems; 2015: IEEE.

580. Skoczyński P. Analysis of solutions improving safety of cyclists in the road traffic. Applied Sciences. 2021;11(9):3771.

581. Stojanová H, Blašková V. Cost benefit study of a safety campaign’s impact on road safety. Accident Analysis & Prevention. 2018;117:205-15.

582. Tarko AP, Li M, Romero M, Thomaz J. A Systematic Approach to Identifying Traffic Safety Needs and Intervention Programs for Indiana. 2014.

583. TOLEDO WHRE. A functional perspective on social marketing: insights from Israel's bicycle helmet campaign. Journal of health communication. 1997;2(3):145-56.

584. Treviño-Siller S, Alvarez F, Flores J, Cruz A, Lozano FJ, editors. Integral strategy to educate Mexican youngsters in road traffic accident prevention. ICERI2010 Proceedings; 2010: IATED.

585. Vahidnia F, Walsh J. Cost-effectiveness of traffic safety interventions in the United States. 2002.

586. Wang X, Leckie C, Xie H, Vaithianathan T, editors. Discovering the impact of urban traffic interventions using contrast mining on vehicle trajectory data. Advances in Knowledge Discovery and Data Mining: 19th Pacific-Asia Conference, PAKDD 2015, Ho Chi Minh City, Vietnam, May 19-22, 2015, Proceedings, Part I 19; 2015: Springer.

587. Wentz R, Roberts I, Bunn F, Edwards P, Kwan I, Lefebvre C. Identifying controlled evaluation studies of road safety interventions: searching for needles in a haystack. Elsevier; 2001. p. 267-76.

588. Wesson HK, Boikhutso N, Hyder AA, Bertram M, Hofman KJ. Informing road traffic intervention choices in South Africa: the role of economic evaluations. Global health action. 2016;9(1):30728.

589. Yahia HA, Ismail A, Albrka SI, Ladin MA, Almselati AS. Implementing specific interventions to reduce road traffic accidents. 2014.

590. Yang B-M, Kim J. Road traffic accidents and policy interventions in Korea. Injury control and safety promotion. 2003;10(1-2):89-94.

591. Zia H, Durdin P, Harris D. An automated process of identifying high-risk roads for speed management intervention. Journal of the Australasian College of Road Safety. 2016;27(4):43-8.
