# Supplementary material for: Effectiveness of interventions for preventing road traffic injuries: A systematic review in low-, middle- and high-income countries
Source: PLoS One. 2024 Dec 5;19(12):e0312428. doi: 10.1371/journal.pone.0312428 (PMC11620428; doi:10.1371/journal.pone.0312428)
Supplement: S3 Table — (DOCX) [file pone.0312428.s007.docx]

| **S3 Table. Risk of bias findings** | | | | | | | |
| --- | --- | --- | --- | --- | --- | --- | --- |
| **Risk of Bias** | **Total**  **(N= 852)** | **Study Designs** | | | | | |
|  |  | Before and after  (n= 473) | Case-control study  (n= 27) | Cohort study  (n= 36) | Cross-sectional study  (n= 133) | Randomized controlled trial  (n= 40) | Others  (n= 143) |
| **Low** | 716 | 412 | 26 | 31 | 111 | 26 | 110 |
| **Moderate** | 115 | 49 | 0 | 5 | 19 | 13 | 29 |
| **Height** | 21 | 12 | 1 | 0 | 3 | 1 | 4 |
