# Supplementary material for: Effectiveness of interventions for preventing road traffic injuries: A systematic review in low-, middle- and high-income countries
Source: PLoS One. 2024 Dec 5;19(12):e0312428. doi: 10.1371/journal.pone.0312428 (PMC11620428; doi:10.1371/journal.pone.0312428)
Supplement: S4 Table — (DOCX) [file pone.0312428.s008.docx]

| **S4 Table. Relationship between prevention type and intervention outcomes (Chi Square Test)** | | | |
| --- | --- | --- | --- |
| **Prevention Types** | **Total (N= 852)** | **Outcomes** | |
|  |  | **Effective**  **(n= 695)** | **non-effective**  **(n= 157)** |
| **Education** | 120 (14.1%) | 97 (14.0%) | 23 (14.6%) |
| **Law enforcement** | 145 (17.0%) | 116 (16.7%) | 29 (18.5%) |
| **Legislation** | 225 (26.4%) | 181 (26.0%) | 44 (28.0%) |
| **Multi intervention** | 48 (5.6%) | 37 (5.3%) | 11 (7.0%) |
| **Road safety** | 138 (16.2%) | 116 (16.7%) | 22 (14.0%) |
| **Social marketing** | 42 (4.9%) | 33 (4.7%) | 9 (5.7%) |
| **Traffic user safety** | 19 (2.2%) | 18 (2.6%) | 1 (0.6%) |
| **Vehicle safety** | 115 (13.5%) | 97 (14.0%) | 18 (11.5%) |
