# Supplementary material for: Effectiveness of interventions for preventing road traffic injuries: A systematic review in low-, middle- and high-income countries
Source: PLoS One. 2024 Dec 5;19(12):e0312428. doi: 10.1371/journal.pone.0312428 (PMC11620428; doi:10.1371/journal.pone.0312428)
Supplement: S5 Table — (DOCX) [file pone.0312428.s009.docx]

| **S5 Table. Relationship between level income and intervention outcomes (Chi Square Test)** | | | |
| --- | --- | --- | --- |
| **Level Incomes** | **Total (N= 852)** | **Outcomes** | |
|  |  | **Effective**  **(n= 695)** | **Non-effective**  **(n= 157)** |
| **High income** | 713 (83.8%) | 581 (83.6%) | 132 (84.6%) |
| **Law enforcement** | 8 (0.9%) | 7 (1.0%) | 1 (0.6%) |
| **Low income** | 42 (4.9%) | 40 (5.8%) | 2 (1.3%) |
| **multi intervention** | 88 (10.3%) | 67 (9.6%) | 21 (13.5%) |
