# Supplementary material for: Effectiveness of interventions for preventing road traffic injuries: A systematic review in low-, middle- and high-income countries
Source: PLoS One. 2024 Dec 5;19(12):e0312428. doi: 10.1371/journal.pone.0312428 (PMC11620428; doi:10.1371/journal.pone.0312428)
Supplement: S6 Table — (DOCX) [file pone.0312428.s010.docx]

| **S6 Table. Relationship between target population and intervention outcomes (Chi Square Test)** | | | |
| --- | --- | --- | --- |
| **Target Population** | **Total (N= 852)** | **Outcomes** | |
|  |  | **Effective**  **(n= 695)** | **Non-effective**  **(n= 157)** |
| All road users | 182 (21.4%) | 147 (21.2%) | 35 (22.3%) |
| Bicyclists | 61 (7.2%) | 50 (7.2%) | 11 (7.0%) |
| Drivers | 391 (45.9%) | 313 (45.0%) | 78 (49.7%) |
| Motorcyclists | 54 (6.3%) | 48 (6.9%) | 6 (3.8%) |
| Multi-targeted population | 73 (8.6%) | 67 (9.6%) | 6 (3.8%) |
| Passengers | 51 (6.0%) | 39 (5.6%) | 12 (7.6%) |
| Pedestrians | 40 (4.7%) | 31 (4.5%) | 9 (5.7%) |
