# Supplementary material for: Effectiveness of interventions for preventing road traffic injuries: A systematic review in low-, middle- and high-income countries
Source: PLoS One. 2024 Dec 5;19(12):e0312428. doi: 10.1371/journal.pone.0312428 (PMC11620428; doi:10.1371/journal.pone.0312428)
Supplement: S7 Table — (DOCX) [file pone.0312428.s011.docx]

| **S7 Table. Relationship between Level of implementation and intervention outcomes (Chi Square Test)** | | | |
| --- | --- | --- | --- |
| **Level of implementation** | **Total (N= 852)** | **Outcomes** | |
|  |  | **Effective**  **(n= 695)** | **Non-effective**  **(n= 157)** |
| International | 29 (3.4%) | 23 (3.3%) | 6 (3.8) |
| National | 360 (42.3%) | 288 (41.4%) | 72 (45.9%) |
| Subnational | 463 (54.3%) | 384 (55.3%) | 79 (50.3%) |

.
