# Supplementary material for: Effectiveness of interventions for preventing road traffic injuries: A systematic review in low-, middle- and high-income countries
Source: PLoS One. 2024 Dec 5;19(12):e0312428. doi: 10.1371/journal.pone.0312428 (PMC11620428; doi:10.1371/journal.pone.0312428)
Supplement: S8 Table — (DOCX) [file pone.0312428.s012.docx]

| **S8 Table. Relationship between year of the study and intervention outcomes (Chi Square Test)** | | | |
| --- | --- | --- | --- |
| **Year of the study** | **Total (N= 852)** | **Outcomes** | |
|  |  | **Effective**  **(n= 695)** | **Non-effective**  **(n= 157)** |
| **<1999 year** | 244 (28.6%) | 194 (27.9%) | 50 (31.8%) |
| **2000-2010 year** | 218 (25.6%) | 176 (25.3%) | 42 (26.8%) |
| **2011-2020 year** | 306 (35.9%) | 251 (36.1%) | 55 (35.0%) |
| **>2020 year** | 84 (9.9%) | 74 (10.6%) | 10 (6.4%) |
