# Supplementary material for: Effectiveness of interventions for preventing road traffic injuries: A systematic review in low-, middle- and high-income countries
Source: PLoS One. 2024 Dec 5;19(12):e0312428. doi: 10.1371/journal.pone.0312428 (PMC11620428; doi:10.1371/journal.pone.0312428)
Supplement: S9 Table — (DOCX) [file pone.0312428.s013.docx]

| **S9 Table. Relationship between year of the study and intervention type (Chi Square Test)** | | | | | | | | | | |
| --- | --- | --- | --- | --- | --- | --- | --- | --- | --- | --- |
| **Year** | **Total**  **(N= 852)** | **Intervention Types** | | | | | | | | |
|  |  | **Education**  **(n= 120)** | **Law enforcement**  **(n= 145)** | **Legislation**  **(n= 225)** | | **Multi intervention**  **(n= 48)** | **Road safety**  **(n= 138)** | **Social marketing**  **(n= 42)** | **Traffic user safety**  **(n= 19)** | **Vehicle safety**  **(n= 115)** |
| **<1999** | 244  (28.6%) | 25  (20.8%) | 37  (25.5%) | 99  (44.0%) | | 18  (37.5%) | 14  (10.1%) | 13  (31.0%) | 3  (15.8%) | 35  (30.4%) |
| **2000-2010** | 218  (25.6%) | 21  (17.5%) | 53  (36.6%) | | 54  (24.0%) | 16  (33.3%) | 26  (18.8%) | 11  (26.2%) | 5  (26.3%) | 32  (27.8%) |
| **2011-2020** | 306  (35.9%) | 55  (45.8%) | 37  (25.5%) | | 63  (28.0%) | 8  (16.7%) | 82  (59.4%) | 14  (33.3%) | 7  (36.8%) | 40  (34.8%) |
| **>2020** | 84  (9.9%) | 19  (15.8%) | 18  (12.4%) | | 9  (4.0%) | 6  (12.5%) | 16  (11.6%) | 4  (9.5%) | 4  (21.1%) | 8  (7.0%) |
