# Supplementary material for: Effectiveness of interventions for preventing road traffic injuries: A systematic review in low-, middle- and high-income countries
Source: PLoS One. 2024 Dec 5;19(12):e0312428. doi: 10.1371/journal.pone.0312428 (PMC11620428; doi:10.1371/journal.pone.0312428)
Supplement: Attachment — Submitted filename: Review comments_RTI.docx [file pone.0312428.s015.docx]

**Review comments to the author:**

**Title: Effectiveness of Interventions for Preventing Road Traffic Injuries: A Systematic**

**Review in Low-, Middle- and High-income Countries**

Dear author,
Congratulation for an insightful systematic review article. Whilst I don't have any major issue with this article, but I have the following concerns regarding the paper:

- Overall comment:

For systematic review, analysis of the included studies in different angle was well observed in this study, which is the strength of the study. The tables were made logically and well described.

- On page 2 in abstract part, please add some search engine name in the methods part.
- The objective could be modified like, to assess the effectiveness of interventions which were designed to reduce road traffic injuries (RTIs) in low-, middle-, and high-income countries.
- In the article, more recent studies can be included for systematic review (up to 2021-2024 there were 2 studies included only).
- The methodology mentioned qualitative meta-analysis, however, the study seemed more like narrative synthesis , the data analysis section (from line 189 onwards) needs more clarification for readers.
- There was some typo in the article. (like safety, traffic in figure 4) , please make necessary corrections
