# Supplementary material for: Effectiveness of interventions for preventing road traffic injuries: A systematic review in low-, middle- and high-income countries
Source: PLoS One. 2024 Dec 5;19(12):e0312428. doi: 10.1371/journal.pone.0312428 (PMC11620428; doi:10.1371/journal.pone.0312428)
Supplement: Attachment — Submitted filename: Response to Reviewers.docx [file pone.0312428.s016.docx]

**Manuscript ID**: PONE-D-24-16957

**Title**: Effectiveness of Interventions for Preventing Road Traffic Injuries: A Systematic Review in Low-, Middle- and High-income Countries

Dear Dr. Alharbi.,

We sincerely appreciate the time and effort that the reviewers and the editor have invested in evaluating our manuscript. Thank you very much for your thorough review and constructive feedback, which have undoubtedly contributed to the improvement of our work. We have carefully considered all the comments and suggestions, and have made the necessary revisions to address each point raised. We hope that these revisions meet the reviewers’ expectations and believe that they have enhanced the quality of the manuscript. We are grateful for the insightful feedback and remain open to any further suggestions.

Best regards,

Kamran B Lankarani M.D.

Distinguished Professor of Medicine

Shiraz University of Medical Sciences

IR Iran

**…………………………………..**

**Reviewers' comments:**

**Reviewer #1:**

The article focuses on a pilot study conducted in Lebanon, which may limit the generalizability of the findings to other countries or regions with different agricultural practices, literacy rates, and cultural contexts.

1- The article does not provide details on the sample size used for the pilot study. A larger sample may be needed to ensure the robustness of the validation process and the reliability of the questionnaire.

2- The article does not provide detailed information on the demographic characteristics of the participating farmers, such as age, education level, farming experience, and socioeconomic status. These factors may influence the understanding and practices related to pesticide labels.

3-The item reduction and development of the questionnaire relied on the evaluation and recommendations of a panel of five experts. This process could be subject to individual biases and perspectives, potentially influencing the content and structure of the final questionnaire.

4-The article does not mention any cross-validation of the questionnaire with a separate sample or population. Cross-validation would help assess the stability and generalizability of the questionnaire's psychometric properties.

5- The article focuses on the development and validation of the questionnaire, but does not provide insights into the practical application of the tool in real-world settings or its impact on improving farmers' understanding and practices related to pesticide labels.

6- The article does not specify the time frame of the data collection, which may limit the relevance of the findings if the study was conducted some time ago and agricultural practices or labeling regulations have since changed.

7- The article does not compare the newly developed questionnaire to other existing tools for assessing pesticide label comprehension and practices, which could help establish its added value or unique features.

**Response**:

Thank you very much for your thorough review and evaluation of our manuscript. We greatly appreciate the feedback provided, as it is invaluable in enhancing the quality of scientific publications. However, it seems that the comments provided pertain to a study focused on the development and validation of a questionnaire related to farmers' understanding of pesticide labels. Our article, titled "Effectiveness of Interventions for Preventing Road Traffic Injuries: A Systematic Review in Low-, Middle- and High-income Countries," is a systematic review that focuses on evaluating interventions for preventing road traffic injuries. It appears that the comments may not be directly related to our manuscript. We would kindly request that the comments be reassessed in relation to the content of our article. This would help us ensure that we address relevant feedback and improve our manuscript effectively. Thank you again for your time and consideration.

**Reviewer #2:**

Overall the analysis was done in multi-dimensional angle and info graph were well presented. Few typos were found in the background and figure caption. My other comments were uploaded as an attachment.

**Response:**

Thank you very much for your positive feedback on our manuscript. We appreciate your acknowledgment of the multi-dimensional analysis and the presentation of the info graphics. Your keen eye in identifying a few typos in the background and figure captions is much appreciated. We will carefully review and correct these typos to improve the overall quality of the manuscript. Additionally, we will thoroughly go through the other comments you have provided in the attached file and address them accordingly. Thank you again for your valuable insights and constructive feedback.

Dear author,
Congratulation for an insightful systematic review article. Whilst I don't have any major issue with this article, but I have the following concerns regarding the paper:

- Overall comment:

For systematic review, analysis of the included studies in different angle was well observed in this study, which is the strength of the study. The tables were made logically and well described.

**Response:**

Thank you very much for your positive feedback and for your kind words regarding our systematic review article. We are pleased to hear that you found the analysis of the included studies from different angles to be a strength of our work, and that the tables were logically constructed and well described. We appreciate your recognition of these aspects, and we are committed to ensuring that the final manuscript reflects the high standards expected. Should you have any further suggestions or concerns, we would be more than happy to address them. Thank you again for your valuable feedback.

- On page 2 in abstract part, please add some search engine name in the methods part.

**Response:**

Thank you for your valuable point. We appreciate your suggestion regarding the methods section in the abstract. We added the names of the search engines used during our systematic review in abstract section. Thank you again for your insightful comments.

- The objective could be modified like, to assess the effectiveness of interventions which were designed to reduce road traffic injuries (RTIs) in low-, middle-, and high-income countries.

**Response:**

Thank you for your thoughtful suggestion regarding the modification of the objective. We agree that the proposed wording enhances the clarity and precision of our objective statement. We revised the objective in the introduction section of the manuscript to reflect this improved phrasing: "to assess the effectiveness of interventions designed to reduce road traffic injuries (RTIs) in low-, middle-, and high-income countries".

- In the article, more recent studies can be included for systematic review (up to 2021-2024 there were 2 studies included only).

**Response:**

Thank you for your observation regarding the inclusion of more recent studies in the systematic review. We would like to clarify that our search was conducted up to December 2023, and we have included all relevant studies published up to that date. In fact, for the year 2023 alone, we have included 15 studies in our review. The details regarding the publication years of the included studies are clearly presented in Figure 2 of the manuscript. We appreciate your attention to this matter and hope this clarification addresses your concern.

- The methodology mentioned qualitative meta-analysis, however, the study seemed more like narrative synthesis, and the data analysis section (from line 189 onwards) needs more clarification for readers.

**Response:**

Thank you for your valuable feedback regarding the methodology section of our manuscript. After carefully considering your comment, we recognized that the term "qualitative meta-analysis" may not have accurately reflected the approach we used. Consequently, we have revised the manuscript to describe our methodology as a "systematic review approach" with a focus on narrative synthesis. We appreciate your insight, which has led to this important clarification in our manuscript. Thank you again for your thoughtful review.

- There was some typo in the article. (like safety, traffic in figure 4), please make necessary corrections

**Response:**

Thank you for bringing the typographical errors to our attention. We have reviewed the manuscript, including Figure 4, and have corrected the typos (such as "safety" and "traffic"). We appreciate your careful review and attention to detail, which helps us improve the quality of our work. Thank you again for your valuable point.

**Reviewer #3:**

This paper attempts to provide a review of past studies investigating the effectiveness of interventions for preventing traffic injuries in low, middle and high income countries. The following issues need to be clarified before this reviewer has a favourable recommendation.

**Response:**

Thank you for your feedback and for taking the time to review our manuscript. We appreciate your acknowledgment of our attempt to provide a comprehensive review of past studies on the effectiveness of interventions for preventing traffic injuries across different income countries. We understand that there are several issues that need to be clarified to meet your expectations. We are committed to addressing each of the specific concerns you have raised and making the necessary revisions to improve the manuscript. Below, we have provided detailed responses to each of your comments.

1. The review is not specific to children injuries. The mention of children injuries in BACKGROUND of Abstract is not needed. Please consider removing it.

**Response:**

Based on this comment, we removed it. Thank you for helping us improve the clarity and consistency of our manuscript.

2. The focuses of this review are broad. While drink driving and restraint use have been the focuses of traffic intervention points in developed countries, other issues such as infrastructures, or perhaps helmet use, might be the focus in developing countries. Comparing the effectiveness of intervention points among developing/developed countries is probably not proper.

**Response:**

Thank you for your thoughtful feedback on the scope of our review. We acknowledge that the focus areas of traffic interventions can vary significantly between developed and developing countries, with different priorities such as drink driving and restraint use in developed countries versus infrastructure improvements and helmet use in developing countries. We agree that directly comparing the effectiveness of interventions across these diverse contexts may not be entirely appropriate due to the differing challenges and intervention strategies. To address this concern, we refined our analysis and discussion sections to clearly distinguish between the interventions focuses in different income settings, rather than attempting to directly compare them. This adjustment could helped provide a more nuanced understanding of the effectiveness of interventions within their specific contexts. It should also be mentioned the situation in low and middle income countries are not homogenous. While remote areas are suffering from weak infrastructures and unsafe vehicles, in the same countries there are well developed regions especially in larger cities in which the problems of driving under influence of ethanol as well as restraint have great impact. However, based on this comment, explanations were added to the discussion. We appreciate your guidance on this matter and believe that these revisions will enhance the relevance and accuracy of our review.

3. Please clarify why a traditional meta-analysis was not conducted.

**Response:**

Thank you for your thoughtful comment regarding the decision not to conduct a traditional meta-analysis. We fully understand the importance of clarity in methodological choices. "In our study, we opted not to perform a traditional meta-analysis due to significant methodological heterogeneity among the selected studies. Specifically, the included studies varied greatly in terms of their design, analysis methods, and the way they reported findings. This level of heterogeneity made it impractical to combine the results in a single meta-analysis without potentially compromising the validity of the conclusions. Instead, we chose a narrative synthesis approach, which allowed us to systematically aggregate and categorize the quantitative findings into themes aligned with our study objectives. This approach provided a structured way to identify common patterns and assess the overall impact of various interventions on road traffic injuries." We hope this explanation clarifies our methodological choices. We have also included this reasoning in the manuscript to ensure that it is clear to all readers. Thank you again for your valuable feedback.

**Journal Requirements:**

1. When submitting your revision, we need you to address these additional requirements.

Please ensure that your manuscript meets PLOS ONE's style requirements, including those for file naming. The PLOS ONE style templates can be found at
https://journals.plos.org/plosone/s/file?id=wjVg/PLOSOne_formatting_sample_main_body.pdf and
https://journals.plos.org/plosone/s/file?id=ba62/PLOSOne_formatting_sample_title_authors_affiliations.pdf.

**Response**:

Thank you for your guidance regarding the PLOS ONE style requirements. We ensured that our revised manuscript fully adheres to the PLOS ONE formatting guidelines, including the appropriate file naming conventions. We carefully reviewed and adjusted our submission according to the style templates provided in the links you shared. We appreciate your attention to detail and your assistance in preparing our manuscript for submission. Thank you again for your support.

2. We note that the grant information you provided in the ‘Funding Information’ and ‘Financial Disclosure’ sections do not match.

When you resubmit, please ensure that you provide the correct grant numbers for the awards you received for your study in the ‘Funding Information’ section.

**Response**:

Thank you for bringing this to our attention. We apologize for any inconsistencies in the grant information provided in the ‘Funding Information’ and ‘Financial Disclosure’ sections. We carefully reviewed and corrected these sections to ensure that the grant numbers and funding details are accurate and consistent across both sections. We appreciate your careful reviewed and guidance, and we made sure these issues are resolved in our resubmission. Thank you again for your assistance.

3. Thank you for stating the following financial disclosure:
 [This study is funded by Shiraz University of Medical Sciences and the Iran National Science Foundation (INSF) (Grant No. 1396-01-104-16210 and Approval Number Ethics IR.SUMS.REC.1397.458)].  
Please state what role the funders took in the study.  If the funders had no role, please state: ""The funders had no role in study design, data collection and analysis, decision to publish, or preparation of the manuscript."

If this statement is not correct you must amend it as needed.

Please include this amended Role of Funder statement in your cover letter; we will change the online submission form on your behalf.

**Response**:

Thank you for your guidance regarding the financial disclosure statement. I would like to confirm that the funders of this study, Shiraz University of Medical Sciences and the Iran National Science Foundation (INSF), had no role in study design, data collection and analysis, decision to publish, or preparation of the manuscript. Please feel free to update the online submission form with the following statement: "The funders had no role in study design, data collection and analysis, decision to publish, or preparation of the manuscript." Thank you for your assistance in this matter

4. Thank you for stating the following in the Acknowledgments Section of your manuscript:
[The authors would like to thank the Shiraz University of Medical Sciences and the Iran National Science Foundation (INSF) for financial support. (Grant No. 1396-01-104-16210 and Approval Number Ethics IR.SUMS.REC.1397.458). This article was derived from M. Akbari's thesis for the fulfillment of obtaining a Ph.D. degree.]
We note that you have provided funding information that is not currently declared in your Funding Statement. However, funding information should not appear in the Acknowledgments section or other areas of your manuscript. We will only publish funding information present in the Funding Statement section of the online submission form.
Please remove any funding-related text from the manuscript and let us know how you would like to update your Funding Statement. Currently, your Funding Statement reads as follows:
 [This study is funded by Shiraz University of Medical Sciences and the Iran National Science Foundation (INSF) (Grant No. 1396-01-104-16210 and Approval Number Ethics IR.SUMS.REC.1397.458)]

Please include your amended statements within your cover letter; we will change the online submission form on your behalf.

**Response**:

Thank you for your guidance regarding the proper placement of funding information. I have removed the funding-related text from the Acknowledgments section of the manuscript as requested. The funding information, including the grant numbers, is now only present in the Funding Statement section. The updated Funding Statement reads as follows:

"This study is funded by Shiraz University of Medical Sciences and the Iran National Science Foundation (INSF) (Grant No. 1396-01-104-16210 and Approval Number Ethics IR.SUMS.REC.1397.458)."

Please update the online submission form accordingly. Thank you for your assistance in ensuring that our manuscript meets the journal's guidelines.

5. In the online submission form, you indicated that [Data will be made available on reasonable request.].
All PLOS journals now require all data underlying the findings described in their manuscript to be freely available to other researchers, either 1. In a public repository, 2. Within the manuscript itself, or 3. Uploaded as supplementary information.
This policy applies to all data except where public deposition would breach compliance with the protocol approved by your research ethics board. If your data cannot be made publicly available for ethical or legal reasons (e.g., public availability would compromise patient privacy), please explain your reasons on resubmission and your exemption request will be escalated for approval.

**Response**:

Thank you for your guidance regarding the data availability requirements. We have prepared the data underlying the findings described in our manuscript and upload them as supplementary information with our resubmission. This ensures that the data will be freely available to other researchers, in compliance with PLOS ONE's data sharing policy. Please let us know if there are any further steps, we should take to ensure that our submission meets all necessary requirements. Thank you for your support.

6. Please include captions for your Supporting Information files at the end of your manuscript, and update any in-text citations to match accordingly. Please see our Supporting Information guidelines for more information: http://journals.plos.org/plosone/s/supporting-information.

**Response**:

Thank you for your comment. We have included captions for our Supporting Information files at the end of the manuscript as requested. Additionally, we have reviewed and updated all in-text citations to ensure they match the Supporting Information files accordingly.

7. Please review your reference list to ensure that it is complete and correct. If you have cited papers that have been retracted, please include the rationale for doing so in the manuscript text, or remove these references and replace them with relevant current references. Any changes to the reference list should be mentioned in the rebuttal letter that accompanies your revised manuscript. If you need to cite a retracted article, indicate the article’s retracted status in the References list and also include a citation and full reference for the retraction notice.

**Response**:

Thank you for your guidance regarding the review of our reference list. We have thoroughly reviewed our references using EndNote and confirmed that none of the cited papers have been retracted. Thank you again for your assistance.
