# Supplementary material for: Effectiveness of interventions for preventing road traffic injuries: A systematic review in low-, middle- and high-income countries
Source: PLoS One. 2024 Dec 5;19(12):e0312428. doi: 10.1371/journal.pone.0312428 (PMC11620428; doi:10.1371/journal.pone.0312428)
Supplement: Peer review history

# Peer review: Decision Letter 0

**DOI:** 10.1371/journal.pone.0312428.r001

**Authors:** Rayan Jafnan Alharbi

18 Jul 2024

PONE-D-24-16957Effectiveness of Interventions for Preventing Road Traffic Injuries: A Systematic Review in Low-, Middle- and High-income CountriesPLOS ONE

Dear Dr. Bagheri Lankarani,

Thank you for submitting your manuscript to PLOS ONE. After careful consideration, we feel that it has merit but does not fully meet PLOS ONE’s publication criteria as it currently stands. Therefore, we invite you to submit a revised version of the manuscript that addresses the points raised during the review process.

Please submit your revised manuscript by Aug 31 2024 11:59PM. If you will need more time than this to complete your revisions, please reply to this message or contact the journal office at plosone@plos.org. When you're ready to submit your revision, log on to https://www.editorialmanager.com/pone/ and select the 'Submissions Needing Revision' folder to locate your manuscript file.

Please include the following items when submitting your revised manuscript:

- A rebuttal letter that responds to each point raised by the academic editor and reviewer(s). You should upload this letter as a separate file labeled 'Response to Reviewers'.
- A marked-up copy of your manuscript that highlights changes made to the original version. You should upload this as a separate file labeled 'Revised Manuscript with Track Changes'.
- An unmarked version of your revised paper without tracked changes. You should upload this as a separate file labeled 'Manuscript'.

If you would like to make changes to your financial disclosure, please include your updated statement in your cover letter. Guidelines for resubmitting your figure files are available below the reviewer comments at the end of this letter.

If applicable, we recommend that you deposit your laboratory protocols in protocols.io to enhance the reproducibility of your results. Protocols.io assigns your protocol its own identifier (DOI) so that it can be cited independently in the future. For instructions see: https://journals.plos.org/plosone/s/submission-guidelines#loc-laboratory-protocols. Additionally, PLOS ONE offers an option for publishing peer-reviewed Lab Protocol articles, which describe protocols hosted on protocols.io. Read more information on sharing protocols at https://plos.org/protocols?utm_medium=editorial-email&utm_source=authorletters&utm_campaign=protocols.

We look forward to receiving your revised manuscript.

Kind regards,

Rayan Jafnan Alharbi, Ph.D

Academic Editor

PLOS ONE

Journal Requirements:

1. When submitting your revision, we need you to address these additional requirements.

Please ensure that your manuscript meets PLOS ONE's style requirements, including those for file naming. The PLOS ONE style templates can be found at

https://journals.plos.org/plosone/s/file?id=wjVg/PLOSOne_formatting_sample_main_body.pdf and

https://journals.plos.org/plosone/s/file?id=ba62/PLOSOne_formatting_sample_title_authors_affiliations.pdf.

2. We note that the grant information you provided in the ‘Funding Information’ and ‘Financial Disclosure’ sections do not match.

When you resubmit, please ensure that you provide the correct grant numbers for the awards you received for your study in the ‘Funding Information’ section.

3. Thank you for stating the following financial disclosure:

 [This study is funded by Shiraz University of Medical Sciences and the Iran National Science Foundation (INSF) (Grant No. 1396-01-104-16210 and Approval Number Ethics IR.SUMS.REC.1397.458)].  

Please state what role the funders took in the study.  If the funders had no role, please state: ""The funders had no role in study design, data collection and analysis, decision to publish, or preparation of the manuscript.""

If this statement is not correct you must amend it as needed.

Please include this amended Role of Funder statement in your cover letter; we will change the online submission form on your behalf.

4. Thank you for stating the following in the Acknowledgments Section of your manuscript:

[The authors would like to thank the Shiraz University of Medical Sciences and the Iran National Science Foundation (INSF) for financial support. (Grant No. 1396-01-104-16210 and Approval Number Ethics IR.SUMS.REC.1397.458). This article was derived from M. Akbari's thesis for the fulfillment of obtaining a Ph.D. degree.]

We note that you have provided funding information that is not currently declared in your Funding Statement. However, funding information should not appear in the Acknowledgments section or other areas of your manuscript. We will only publish funding information present in the Funding Statement section of the online submission form.

Please remove any funding-related text from the manuscript and let us know how you would like to update your Funding Statement. Currently, your Funding Statement reads as follows:

 [This study is funded by Shiraz University of Medical Sciences and the Iran National Science Foundation (INSF) (Grant No. 1396-01-104-16210 and Approval Number Ethics IR.SUMS.REC.1397.458)]

Please include your amended statements within your cover letter; we will change the online submission form on your behalf.

5. In the online submission form, you indicated that [Data will be made available on reasonable request.].

All PLOS journals now require all data underlying the findings described in their manuscript to be freely available to other researchers, either 1. In a public repository, 2. Within the manuscript itself, or 3. Uploaded as supplementary information.

This policy applies to all data except where public deposition would breach compliance with the protocol approved by your research ethics board. If your data cannot be made publicly available for ethical or legal reasons (e.g., public availability would compromise patient privacy), please explain your reasons on resubmission and your exemption request will be escalated for approval.

6. Please include captions for your Supporting Information files at the end of your manuscript, and update any in-text citations to match accordingly. Please see our Supporting Information guidelines for more information: http://journals.plos.org/plosone/s/supporting-information.

7. Please review your reference list to ensure that it is complete and correct. If you have cited papers that have been retracted, please include the rationale for doing so in the manuscript text, or remove these references and replace them with relevant current references. Any changes to the reference list should be mentioned in the rebuttal letter that accompanies your revised manuscript. If you need to cite a retracted article, indicate the article’s retracted status in the References list and also include a citation and full reference for the retraction notice.

[Note: HTML markup is below. Please do not edit.]

Reviewers' comments:

Reviewer's Responses to Questions

**Comments to the Author**

1. Is the manuscript technically sound, and do the data support the conclusions?

The manuscript must describe a technically sound piece of scientific research with data that supports the conclusions. Experiments must have been conducted rigorously, with appropriate controls, replication, and sample sizes. The conclusions must be drawn appropriately based on the data presented. 

Reviewer #1: Yes

Reviewer #2: Yes

Reviewer #3: Yes

**********

2. Has the statistical analysis been performed appropriately and rigorously? 

Reviewer #1: Yes

Reviewer #2: Yes

Reviewer #3: Yes

**********

3. Have the authors made all data underlying the findings in their manuscript fully available?

The PLOS Data policy requires authors to make all data underlying the findings described in their manuscript fully available without restriction, with rare exception (please refer to the Data Availability Statement in the manuscript PDF file). The data should be provided as part of the manuscript or its supporting information, or deposited to a public repository. For example, in addition to summary statistics, the data points behind means, medians and variance measures should be available. If there are restrictions on publicly sharing data—e.g. participant privacy or use of data from a third party—those must be specified.

Reviewer #1: Yes

Reviewer #2: Yes

Reviewer #3: Yes

**********

4. Is the manuscript presented in an intelligible fashion and written in standard English?

PLOS ONE does not copyedit accepted manuscripts, so the language in submitted articles must be clear, correct, and unambiguous. Any typographical or grammatical errors should be corrected at revision, so please note any specific errors here.

Reviewer #1: Yes

Reviewer #2: Yes

Reviewer #3: Yes

**********

5. Review Comments to the Author

Please use the space provided to explain your answers to the questions above. You may also include additional comments for the author, including concerns about dual publication, research ethics, or publication ethics. (Please upload your review as an attachment if it exceeds 20,000 characters)

Reviewer #1: The article focuses on a pilot study conducted in Lebanon, which may limit the generalizability of the findings to other countries or regions with different agricultural practices, literacy rates, and cultural contexts.

1- The article does not provide details on the sample size used for the pilot study. A larger sample may be needed to ensure the robustness of the validation process and the reliability of the questionnaire.

2- The article does not provide detailed information on the demographic characteristics of the participating farmers, such as age, education level, farming experience, and socioeconomic status. These factors may influence the understanding and practices related to pesticide labels.

3-The item reduction and development of the questionnaire relied on the evaluation and recommendations of a panel of five experts. This process could be subject to individual biases and perspectives, potentially influencing the content and structure of the final questionnaire.

4-The article does not mention any cross-validation of the questionnaire with a separate sample or population. Cross-validation would help assess the stability and generalizability of the questionnaire's psychometric properties.

5- The article focuses on the development and validation of the questionnaire, but does not provide insights into the practical application of the tool in real-world settings or its impact on improving farmers' understanding and practices related to pesticide labels.

6- The article does not specify the time frame of the data collection, which may limit the relevance of the findings if the study was conducted some time ago and agricultural practices or labeling regulations have since changed.

7- The article does not compare the newly developed questionnaire to other existing tools for assessing pesticide label comprehension and practices, which could help establish its added value or unique features.

Reviewer #2: Overall the analysis was done in multi-dimensional angle and info graph were well presented. Few typos were found in the background and figure caption. My other comments were uploaded as an attachment.

Reviewer #3: This paper attempts to provide a review of past studies investigating the effectiveness of interventions for preventing traffic injuries in low, middle and high income countries. The following issues need to be clarified before this reviewer has a favourable recommendation.

1. The review is not specific to children injuries. The mention of children injuries in BACKGROUND of Abstract is not needed. Please consider removing it.

2. The focuses of this review are broad. While drink driving and restraint use have been the focuses of traffic intervention points in developed countries, other issues such as infrastructures, or perhaps helmet use, might be the focus in developing countries. Comparing the effectiveness of intervention points among developing/developed countries is probably not proper.

3. Please clarify why a traditional meta analysis was not conducted.

**********

6. PLOS authors have the option to publish the peer review history of their article (what does this mean?). If published, this will include your full peer review and any attached files.

If you choose “no”, your identity will remain anonymous but your review may still be made public.

**Do you want your identity to be public for this peer review?** For information about this choice, including consent withdrawal, please see our Privacy Policy.

Reviewer #1: No

Reviewer #2: No

Reviewer #3: No

**********

[NOTE: If reviewer comments were submitted as an attachment file, they will be attached to this email and accessible via the submission site. Please log into your account, locate the manuscript record, and check for the action link "View Attachments". If this link does not appear, there are no attachment files.]

While revising your submission, please upload your figure files to the Preflight Analysis and Conversion Engine (PACE) digital diagnostic tool, https://pacev2.apexcovantage.com/. PACE helps ensure that figures meet PLOS requirements. To use PACE, you must first register as a user. Registration is free. Then, login and navigate to the UPLOAD tab, where you will find detailed instructions on how to use the tool. If you encounter any issues or have any questions when using PACE, please email PLOS at figures@plos.org. Please note that Supporting Information files do not need this step.

Attachment Submitted filename: Review comments_RTI.docx

# Author comment: Author response to Decision Letter 0

**DOI:** 10.1371/journal.pone.0312428.r002

3 Sep 2024

Manuscript ID: PONE-D-24-16957

Title: Effectiveness of Interventions for Preventing Road Traffic Injuries: A Systematic Review in Low-, Middle- and High-income Countries

Dear Dr. Alharbi., 

We sincerely appreciate the time and effort that the reviewers and the editor have invested in evaluating our manuscript. Thank you very much for your thorough review and constructive feedback, which have undoubtedly contributed to the improvement of our work. We have carefully considered all the comments and suggestions, and have made the necessary revisions to address each point raised. We hope that these revisions meet the reviewers’ expectations and believe that they have enhanced the quality of the manuscript. We are grateful for the insightful feedback and remain open to any further suggestions.

Best regards, 

Kamran B Lankarani M.D.

Distinguished Professor of Medicine

Shiraz University of Medical Sciences

IR Iran 

…………………………………..

Reviewers' comments:

Reviewer #1: 

The article focuses on a pilot study conducted in Lebanon, which may limit the generalizability of the findings to other countries or regions with different agricultural practices, literacy rates, and cultural contexts.

1- The article does not provide details on the sample size used for the pilot study. A larger sample may be needed to ensure the robustness of the validation process and the reliability of the questionnaire.

2- The article does not provide detailed information on the demographic characteristics of the participating farmers, such as age, education level, farming experience, and socioeconomic status. These factors may influence the understanding and practices related to pesticide labels.

3-The item reduction and development of the questionnaire relied on the evaluation and recommendations of a panel of five experts. This process could be subject to individual biases and perspectives, potentially influencing the content and structure of the final questionnaire.

4-The article does not mention any cross-validation of the questionnaire with a separate sample or population. Cross-validation would help assess the stability and generalizability of the questionnaire's psychometric properties. 

5- The article focuses on the development and validation of the questionnaire, but does not provide insights into the practical application of the tool in real-world settings or its impact on improving farmers' understanding and practices related to pesticide labels.

6- The article does not specify the time frame of the data collection, which may limit the relevance of the findings if the study was conducted some time ago and agricultural practices or labeling regulations have since changed.

7- The article does not compare the newly developed questionnaire to other existing tools for assessing pesticide label comprehension and practices, which could help establish its added value or unique features.

Response: 

Thank you very much for your thorough review and evaluation of our manuscript. We greatly appreciate the feedback provided, as it is invaluable in enhancing the quality of scientific publications. However, it seems that the comments provided pertain to a study focused on the development and validation of a questionnaire related to farmers' understanding of pesticide labels. Our article, titled "Effectiveness of Interventions for Preventing Road Traffic Injuries: A Systematic Review in Low-, Middle- and High-income Countries," is a systematic review that focuses on evaluating interventions for preventing road traffic injuries. It appears that the comments may not be directly related to our manuscript. We would kindly request that the comments be reassessed in relation to the content of our article. This would help us ensure that we address relevant feedback and improve our manuscript effectively. Thank you again for your time and consideration.

Reviewer #2: 

Overall the analysis was done in multi-dimensional angle and info graph were well presented. Few typos were found in the background and figure caption. My other comments were uploaded as an attachment.

Response: 

Thank you very much for your positive feedback on our manuscript. We appreciate your acknowledgment of the multi-dimensional analysis and the presentation of the info graphics. Your keen eye in identifying a few typos in the background and figure captions is much appreciated. We will carefully review and correct these typos to improve the overall quality of the manuscript. Additionally, we will thoroughly go through the other comments you have provided in the attached file and address them accordingly. Thank you again for your valuable insights and constructive feedback.

Dear author,

Congratulation for an insightful systematic review article. Whilst I don't have any major issue with this article, but I have the following concerns regarding the paper:

• Overall comment:

For systematic review, analysis of the included studies in different angle was well observed in this study, which is the strength of the study. The tables were made logically and well described. 

Response: 

Thank you very much for your positive feedback and for your kind words regarding our systematic review article. We are pleased to hear that you found the analysis of the included studies from different angles to be a strength of our work, and that the tables were logically constructed and well described. We appreciate your recognition of these aspects, and we are committed to ensuring that the final manuscript reflects the high standards expected. Should you have any further suggestions or concerns, we would be more than happy to address them. Thank you again for your valuable feedback.

• On page 2 in abstract part, please add some search engine name in the methods part.

Response: 

Thank you for your valuable point. We appreciate your suggestion regarding the methods section in the abstract. We added the names of the search engines used during our systematic review in abstract section. Thank you again for your insightful comments.

• The objective could be modified like, to assess the effectiveness of interventions which were designed to reduce road traffic injuries (RTIs) in low-, middle-, and high-income countries. 

Response: 

Thank you for your thoughtful suggestion regarding the modification of the objective. We agree that the proposed wording enhances the clarity and precision of our objective statement. We revised the objective in the introduction section of the manuscript to reflect this improved phrasing: "to assess the effectiveness of interventions designed to reduce road traffic injuries (RTIs) in low-, middle-, and high-income countries". 

• In the article, more recent studies can be included for systematic review (up to 2021-2024 there were 2 studies included only). 

Response: 

Thank you for your observation regarding the inclusion of more recent studies in the systematic review. We would like to clarify that our search was conducted up to December 2023, and we have included all relevant studies published up to that date. In fact, for the year 2023 alone, we have included 15 studies in our review. The details regarding the publication years of the included studies are clearly presented in Figure 2 of the manuscript. We appreciate your attention to this matter and hope this clarification addresses your concern.

• The methodology mentioned qualitative meta-analysis, however, the study seemed more like narrative synthesis, and the data analysis section (from line 189 onwards) needs more clarification for readers.

Response: 

Thank you for your valuable feedback regarding the methodology section of our manuscript. After carefully considering your comment, we recognized that the term "qualitative meta-analysis" may not have accurately reflected the approach we used. Consequently, we have revised the manuscript to describe our methodology as a "systematic review approach" with a focus on narrative synthesis. We appreciate your insight, which has led to this important clarification in our manuscript. Thank you again for your thoughtful review.

• There was some typo in the article. (like safety, traffic in figure 4), please make necessary corrections

Response: 

Thank you for bringing the typographical errors to our attention. We have reviewed the manuscript, including Figure 4, and have corrected the typos (such as "safety" and "traffic"). We appreciate your careful review and attention to detail, which helps us improve the quality of our work. Thank you again for your valuable point.

Reviewer #3: 

This paper attempts to provide a review of past studies investigating the effectiveness of interventions for preventing traffic injuries in low, middle and high income countries. The following issues need to be clarified before this reviewer has a favourable recommendation.

Response: 

Thank you for your feedback and for taking the time to review our manuscript. We appreciate your acknowledgment of our attempt to provide a comprehensive review of past studies on the effectiveness of interventions for preventing traffic injuries across different income countries. We understand that there are several issues that need to be clarified to meet your expectations. We are committed to addressing each of the specific concerns you have raised and making the necessary revisions to improve the manuscript. Below, we have provided detailed responses to each of your comments. 

1. The review is not specific to children injuries. The mention of children injuries in BACKGROUND of Abstract is not needed. Please consider removing it.

Response: 

Based on this comment, we removed it. Thank you for helping us improve the clarity and consistency of our manuscript.

2. The focuses of this review are broad. While drink driving and restraint use have been the focuses of traffic intervention points in developed countries, other issues such as infrastructures, or perhaps helmet use, might be the focus in developing countries. Comparing the effectiveness of intervention points among developing/developed countries is probably not proper.

Response: 

Thank you for your thoughtful feedback on the scope of our review. We acknowledge that the focus areas of traffic interventions can vary significantly between developed and developing countries, with different priorities such as drink driving and restraint use in developed countries versus infrastructure improvements and helmet use in developing countries. We agree that directly comparing the effectiveness of interventions across these diverse contexts may not be entirely appropriate due to the differing challenges and intervention strategies. To address this concern, we refined our analysis and discussion sections to clearly distinguish between the interventions focuses in different income settings, rather than attempting to directly compare them. This adjustment could helped provide a more nuanced understanding of the effectiveness of interventions within their specific contexts. It should also be mentioned the situation in low and middle income countries are not homogenous. While remote areas are suffering from weak infrastructures and unsafe vehicles, in the same countries there are well developed regions especially in larger cities in which the problems of driving under influence of ethanol as well as restraint have great impact. However, based on this comment, explanations were added to the discussion. We appreciate your guidance on this matter and believe that these revisions will enhance the relevance and accuracy of our review.

3. Please clarify why a traditional meta-analysis was not conducted.

Response:

 Thank you for your thoughtful comment regarding the decision not to conduct a traditional meta-analysis. We fully understand the importance of clarity in methodological choices. "In our study, we opted not to perform a traditional meta-analysis due to significant methodological heterogeneity among the selected studies. Specifically, the included studies varied greatly in terms of their design, analysis methods, and the way they reported findings. This level of heterogeneity made it impractical to combine the results in a single meta-analysis without potentially compromising the validity of the conclusions. Instead, we chose a narrative synthesis approach, which allowed us to systematically aggregate and categorize the quantitative findings into themes aligned with our study objectives. This approach provided a structured way to identify common patterns and assess the overall impact of various interventions on road traffic injuries." We hope this explanation clarifies our methodological choices. We have also included this reasoning in the manuscript to ensure that it is clear to all readers. Thank you again for your valuable feedback.

Journal Requirements:

1. When submitting your revision, we need you to address these additional requirements.

Please ensure that your manuscript meets PLOS ONE's style requirements, including those for file naming. The PLOS ONE style templates can be found at

https://journals.plos.org/plosone/s/file?id=wjVg/PLOSOne_formatting_sample_main_body.pdf and

https://journals.plos.org/plosone/s/file?id=ba62/PLOSOne_formatting_sample_title_authors_affiliations.pdf.

Response: 

Thank you for your guidance regarding the PLOS ONE style requirements. We ensured that our revised manuscript fully adheres to the PLOS ONE formatting guidelines, including the appropriate file naming conventions. We carefully reviewed and adjusted our submission according to the style templates provided in the links you shared. We appreciate your attention to detail and your assistance in preparing our manuscript for submission. Thank you again for your support.

2. We note that the grant information you provided in the ‘Funding Information’ and ‘Financial Disclosure’ sections do not match.

When you resubmit, please ensure that you provide the correct grant numbers for the awards you received for your study in the ‘Funding Information’ section.

Response: 

Thank you for bringing this to our attention. We apologize for any inconsistencies in the grant information provided in the ‘Funding Information’ and ‘Financial Disclosure’ sections. We carefully reviewed and corrected these sections to ensure that the grant numbers and funding details are accurate and consistent across both sections. We appreciate your careful reviewed and guidance, and we made sure these issues are resolved in our resubmission. Thank you again for your assistance.

3. Thank you for stating the following financial disclosure:

 [This study is funded by Shiraz University of Medical Sciences and the Iran National Science Foundation (INSF) (Grant No. 1396-01-104-16210 and Approval Number Ethics IR.SUMS.REC.1397.458)]. 

Please state what role the funders took in the study. If the funders had no role, please state: ""The funders had no role in study design, data collection and analysis, decision to publish, or preparation of the manuscript."

 If this statement is not correct you must amend it as needed.

Please include this amended Role of Funder statement in your cover letter; we will change the online submission form on your behalf.

Response: 

Thank you for your guidance regarding the financial disclosure statement. I would like to confirm that the funders of this study, Shiraz University of Medical Sciences and the Iran National Science Foundation (INSF), had no role in study design, data collection and analysis, decision to publish, or preparation of the manuscript. Please feel free to update the online submission form with the following statement: "The funders had no role in study design, data collection and analysis, decision to publish, or preparation of the manuscript." Thank you for your assistance in this matter

4. Thank you for stating the following in the Acknowledgments Section of your manuscript:

[The authors would like to thank the Shiraz University of Medical Sciences and the Iran National Science Foundation (INSF) for financial support. (Grant No. 1396-01-104-16210 and Approval Number Ethics IR.SUMS.REC.1397.458). This article was derived from M. Akbari's thesis for the fulfillment of obtaining a Ph.D. degree.]

We note that you have provided funding information that is not c

Attachment Submitted filename: Response to Reviewers.docx

# Peer review: Decision Letter 1

**DOI:** 10.1371/journal.pone.0312428.r003

**Authors:** Rayan Jafnan Alharbi

8 Oct 2024

Effectiveness of Interventions for Preventing Road Traffic Injuries: A Systematic Review in Low-, Middle- and High-income Countries

PONE-D-24-16957R1

Dear Dr. Lankarani,

We’re pleased to inform you that your manuscript has been judged scientifically suitable for publication and will be formally accepted for publication once it meets all outstanding technical requirements.

Within one week, you’ll receive an e-mail detailing the required amendments. When these have been addressed, you’ll receive a formal acceptance letter and your manuscript will be scheduled for publication.

An invoice will be generated when your article is formally accepted. Please note, if your institution has a publishing partnership with PLOS and your article meets the relevant criteria, all or part of your publication costs will be covered. Please make sure your user information is up-to-date by logging into Editorial Manager at Editorial Manager® and clicking the ‘Update My Information' link at the top of the page. If you have any questions relating to publication charges, please contact our Author Billing department directly at authorbilling@plos.org.

If your institution or institutions have a press office, please notify them about your upcoming paper to help maximize its impact. If they’ll be preparing press materials, please inform our press team as soon as possible -- no later than 48 hours after receiving the formal acceptance. Your manuscript will remain under strict press embargo until 2 pm Eastern Time on the date of publication. For more information, please contact onepress@plos.org.

Kind regards,

Rayan Jafnan Alharbi, Ph.D

Academic Editor

PLOS ONE

Additional Editor Comments (optional):

Reviewers' comments:

Reviewer's Responses to Questions

**Comments to the Author**

1. If the authors have adequately addressed your comments raised in a previous round of review and you feel that this manuscript is now acceptable for publication, you may indicate that here to bypass the “Comments to the Author” section, enter your conflict of interest statement in the “Confidential to Editor” section, and submit your "Accept" recommendation.

Reviewer #2: All comments have been addressed

**********

2. Is the manuscript technically sound, and do the data support the conclusions?

The manuscript must describe a technically sound piece of scientific research with data that supports the conclusions. Experiments must have been conducted rigorously, with appropriate controls, replication, and sample sizes. The conclusions must be drawn appropriately based on the data presented. 

Reviewer #2: Yes

**********

3. Has the statistical analysis been performed appropriately and rigorously? 

Reviewer #2: Yes

**********

4. Have the authors made all data underlying the findings in their manuscript fully available?

The PLOS Data policy requires authors to make all data underlying the findings described in their manuscript fully available without restriction, with rare exception (please refer to the Data Availability Statement in the manuscript PDF file). The data should be provided as part of the manuscript or its supporting information, or deposited to a public repository. For example, in addition to summary statistics, the data points behind means, medians and variance measures should be available. If there are restrictions on publicly sharing data—e.g. participant privacy or use of data from a third party—those must be specified.

Reviewer #2: Yes

**********

5. Is the manuscript presented in an intelligible fashion and written in standard English?

PLOS ONE does not copyedit accepted manuscripts, so the language in submitted articles must be clear, correct, and unambiguous. Any typographical or grammatical errors should be corrected at revision, so please note any specific errors here.

Reviewer #2: Yes

**********

6. Review Comments to the Author

Please use the space provided to explain your answers to the questions above. You may also include additional comments for the author, including concerns about dual publication, research ethics, or publication ethics. (Please upload your review as an attachment if it exceeds 20,000 characters)

Reviewer #2: All comments are accurately addressed. the objectives were revised, search engines names were added and other feedback was also incorporated. Best wishes to author.

**********

7. PLOS authors have the option to publish the peer review history of their article (what does this mean?). If published, this will include your full peer review and any attached files.

If you choose “no”, your identity will remain anonymous but your review may still be made public.

**Do you want your identity to be public for this peer review?** For information about this choice, including consent withdrawal, please see our Privacy Policy.

Reviewer #2: **Yes: **Nawshin Torsha

**********

Attachment Submitted filename: Authors Response To Reviewer Comments.pdf

# Editor report: Acceptance letter

**DOI:** 10.1371/journal.pone.0312428.r004

**Authors:** Rayan Jafnan Alharbi

25 Nov 2024

PONE-D-24-16957R1 

PLOS ONE

Dear Dr. Bagheri Lankarani, 

I'm pleased to inform you that your manuscript has been deemed suitable for publication in PLOS ONE. Congratulations! Your manuscript is now being handed over to our production team.

At this stage, our production department will prepare your paper for publication. This includes ensuring the following:

* All references, tables, and figures are properly cited

* All relevant supporting information is included in the manuscript submission,

* There are no issues that prevent the paper from being properly typeset

If revisions are needed, the production department will contact you directly to resolve them. If no revisions are needed, you will receive an email when the publication date has been set. At this time, we do not offer pre-publication proofs to authors during production of the accepted work. Please keep in mind that we are working through a large volume of accepted articles, so please give us a few weeks to review your paper and let you know the next and final steps. 

Lastly, if your institution or institutions have a press office, please let them know about your upcoming paper now to help maximize its impact. If they'll be preparing press materials, please inform our press team within the next 48 hours. Your manuscript will remain under strict press embargo until 2 pm Eastern Time on the date of publication. For more information, please contact onepress@plos.org.

If we can help with anything else, please email us at customercare@plos.org.

Thank you for submitting your work to PLOS ONE and supporting open access. 

Kind regards, 

PLOS ONE Editorial Office Staff

on behalf of

Dr. Rayan Jafnan Alharbi 

Academic Editor

PLOS ONE
